# *E*-Selective Manganese-Catalyzed Semihydrogenation of Alkynes with H_2_ Directly Employed or In Situ-Generated

**DOI:** 10.1021/acscatal.1c06022

**Published:** 2022-01-31

**Authors:** Ronald A. Farrar-Tobar, Stefan Weber, Zita Csendes, Antonio Ammaturo, Sarah Fleissner, Helmuth Hoffmann, Luis F. Veiros, Karl Kirchner

**Affiliations:** † Institute of Applied Synthetic Chemistry, Vienna University of Technology, Getreidemarkt 9, Vienna A-1060, Austria; ‡ Centro de Química Estrutural and Departamento de Engenharia Química, Instituto Superior Técnico, Universidade de Lisboa, Av Rovisco Pais, Lisboa 1049-001, Portugal

**Keywords:** manganese, alkyl complex, alkynes, semihydrogenation, bisphosphine, DFT calculations, alcoholysis

## Abstract

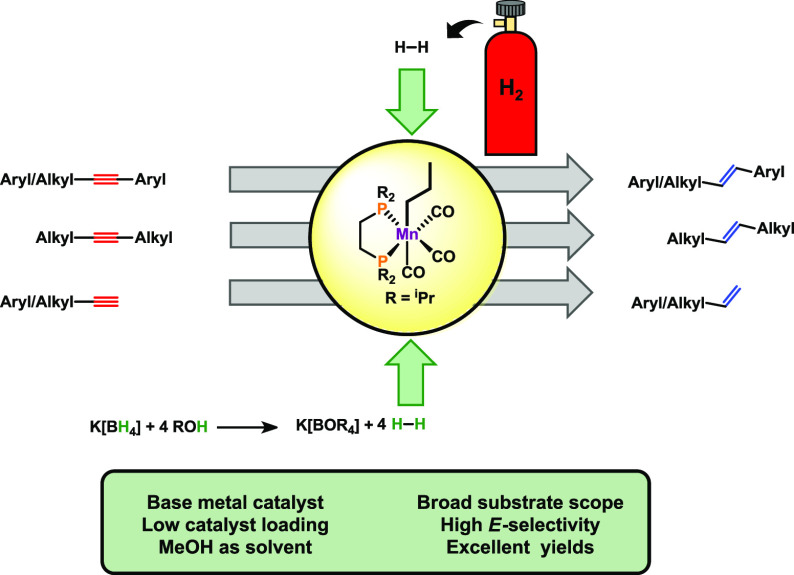

Selective semihydrogenation of alkynes with the Mn(I) alkyl catalyst *fac*-[Mn(dippe)(CO)_3_(CH_2_CH_2_CH_3_)] (dippe = 1,2-bis(di-*iso*-propylphosphino)ethane) as a precatalyst is described. The required hydrogen gas is either directly employed or in situ-generated upon alcoholysis of KBH_4_ with methanol. A series of aryl-aryl, aryl-alkyl, alkyl-alkyl, and terminal alkynes was readily hydrogenated to yield *E-*alkenes in good to excellent isolated yields. The reaction proceeds at 60 °C for directly employed hydrogen or at 60–90 °C with in situ-generated hydrogen and catalyst loadings of 0.5–2 mol %. The implemented protocol tolerates a variety of electron-donating and electron-withdrawing functional groups, including halides, phenols, nitriles, unprotected amines, and heterocycles. The reaction can be upscaled to the gram scale. Mechanistic investigations, including deuterium-labeling studies and density functional theory (DFT) calculations, were undertaken to provide a reasonable reaction mechanism, showing that initially formed *Z-*isomer undergoes fast isomerization to afford the thermodynamically more stable *E-*isomer.

## Introduction

The selective semihydrogenation of alkynes plays a crucial role in bulk industry, fine chemistry, and chemical research.^1^ Examples of relevant applications are found in the hydrocarbon refinery for petrol industry,^1c,2^ the commercial synthetic routes for resveratrol or vitamin A.^1a^ Thus far, traditional systems for the selective formation of *Z*-, *E*-, or terminal alkenes consist of Pd/C,^3^ Lindlar catalyst,^4^ Birch reduction,^5^ Raney nickel,^6^ or Wilkinson catalyst.^7^ Because of limitations regarding selectivity, activity, amount of produced waste, or catalyst price, the study and improvement of these protocols has attracted numerous research groups in academia and industry.^1a,1b,8^ Thus, high activity and excellent selectivity-tailoring is being achieved by combining fine-tuned ligands with noble metals such as Ru,^9^ Rh,^10^ Pd,^11^ and Ir.^12^ During the last decade, the concerns with regard to green chemistry principles have been grown rapidly,^13^ and the development of catalysts based on earth-abundant metals for organic transformations has become very important.^14^ In recent years, several reports on selective alkyne semihydrogenations using catalysts based on Cr,^15^ Mn,^16^ Fe,^17^ Co,^18^ Ni,^19^ and Cu^20^ are described.

**Scheme 1:**
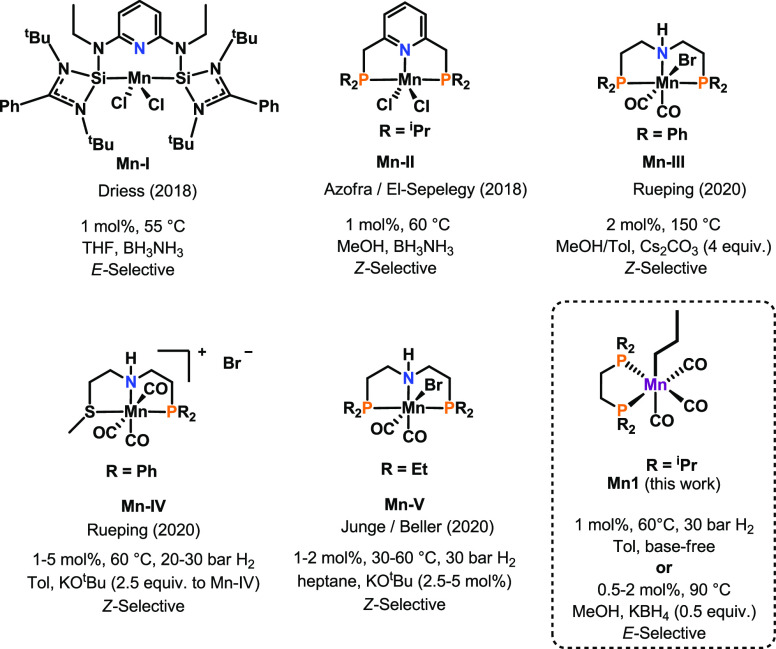
Overview of Manganese-Based Catalysts for the Semihydrogenation of Alkynes

As manganese is concerned, the use of pincer-type ligands and bifunctional catalysis^21^ is crucial in the field of semi(transfer) hydrogenation of alkynes. An overview of manganese-based catalysts for the semihydrogenation and transfer semihydrogenation of alkynes is depicted in Scheme 1. Within this context, ammonia-borane was used as the hydrogen donor to achieve *E*-^16a^ or Z-selective^16b^ semireduction of alkynes. Remarkably, Rueping and coworkers employed methanol as the hydrogen source under forcing conditions (150 °C, 4 equiv Cs_2_CO_3_), yielding *Z*-olefines.^16c^ Furthermore, PNS-^16d^ or PNP-based^16e^ systems were recently employed as hydrogenation catalysts for the *Z*-selective hydrogenation of alkynes upon the addition of KO^t^Bu as the activator.

We have recently reported on the application of the Mn(I) alkyl complex *fac*-[Mn(dippe)(CO)_3_(CH_2_CH_2_CH_3_)] (dippe = 1,2-bis(di-*iso*-propylphosphino)ethane) (**Mn1**) as precatalyst for the hydrogenation of ketones,^22^ nitriles,^23^ alkenes,^24^ CO_2_,^25^ the hydroboration and diboration of alkenes and alkynes,^26^ the dehydrogenative silylation of alkenes,^27^ and the dimerization and cross-coupling of terminal alkynes.^28^ Inspired by these results, we wondered if **Mn1** is also active in the semihydrogenation of alkynes. Herein, we report on the use of **Mn1** as precatalysts for the *E*-selective semihydrogenation of alkynes via two protocols: directly employed hydrogen gas or in situ-generated by the alcoholysis of KBH_4_. In case of generated H_2_, we take advantage of the fact that the BH_4_^–^ anion undergoes fast alcoholysis to generate hydrogen gas,^29^ which is required for the hydrogenation of alkynes without the need of high-pressure equipment.

## Results and Discussion

First, the direct semihydrogenation of alkynes was explored. Upon optimization reactions (for details, see the Supporting Information, Table S1), the efficiency of **Mn1** for the *E*-selective semihydrogenation of various alkynes was examined (Table 1). To our delight, high reactivity and selectivity toward *E*-olefines was observed for unsubstituted or *p*-halide substituted diphenylacetylenes (Table 1, **1b**, **6b–8b**). Although high reactivity and excellent selectivity were observed for substrates with electron-withdrawing groups in the *para*-position, overhydrogenation of 33 and 25% was observed for substrates **4b** and **5b**, respectively. Substrates with electron-donating groups (Table 1, **2b**, and **3b**) gave lower yields and selectivity. Interestingly, only traces of the product were detected in the presence of the strongly coordinating pyridine moiety. In case of aliphatic substrates, high conversions were achieved, thereby leading to moderate *E*/*Z* ratios.

**Table 1:**
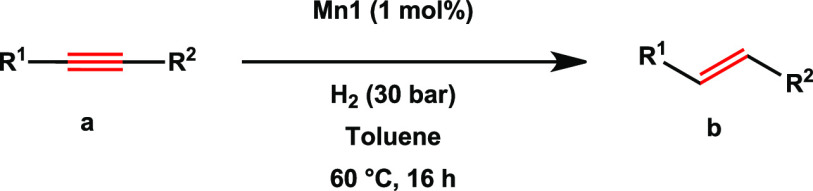

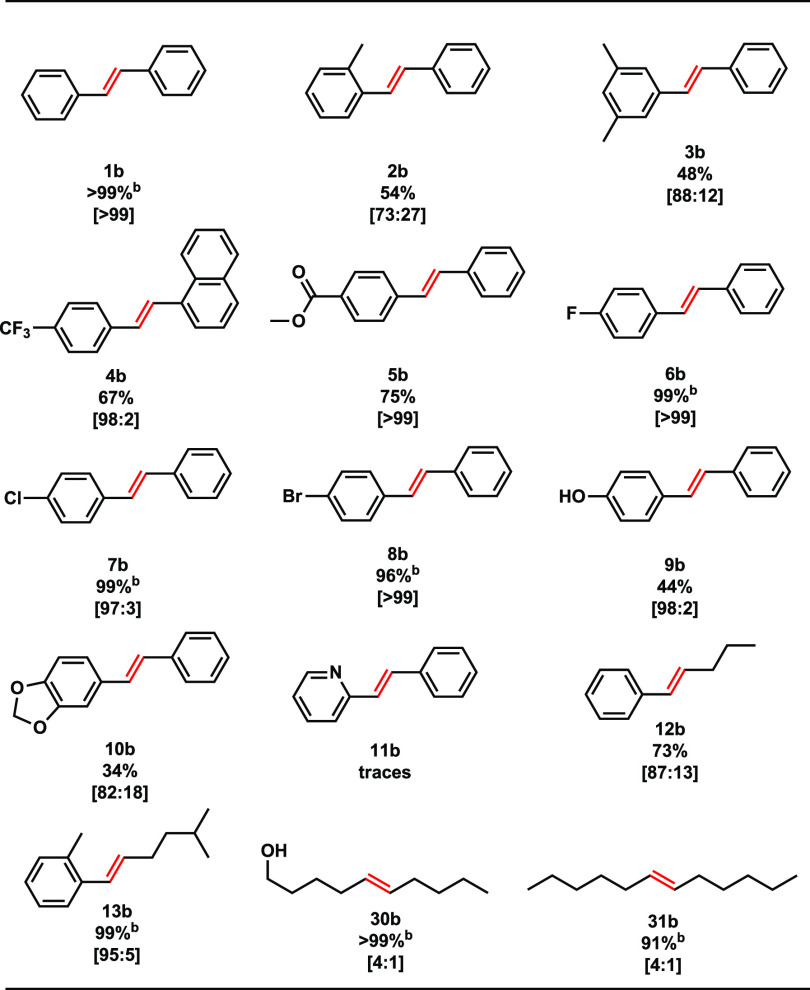
**Mn1**-Catalyzed Semihydrogenation of Alkynes with H_2_^a^

a Reaction conditions: alkyne (0.7 mmol, 1 equiv), **Mn1** (1 mol %), toluene (3.5 mL), 60 °C, 16 h, yield determined by GC using *n*-dodecane as the internal standard. The *E*/*Z* ratio given in square brackets and determined by ^1^H-NMR.

b Isolated yields.

In order to minimize overhydrogenation, we envisioned a system, which is capable of generating hydrogen in situ. This would allow fine-dosing of hydrogen gas in the reaction vessel, which is not achievable in classical hydrogenation reactions, and would therefore suppress alkane formation. Furthermore, in situ-generated H_2_ can be applied in simple reaction setups and does not require the use of high-pressure equipment. Because borohydrides are known to undergo rapid alcoholysis under the release of hydrogen gas, we decided to employ KBH_4_ as the hydrogen donor in combination with alcohols.

In order to establish the best reaction conditions for the semihydrogenation of alkynes, diphenyl acetylene (**1a**) was chosen as the model. When **1a** was treated with 1 mol % of **Mn1** at 60 °C with MeOH and 0.5 equiv of KBH_4_, *E*-stilbene (**1b**) was obtained in 99% isolated yield (Table 2, entry 1). Only traces of *Z*-stilbene (**1c**) were observed, and overhydrogenation did not take place. Encouraged by this finding, temperature, catalyst loading, and solvents were screened (Table 2). With catalyst loadings of 1.0 and 0.5 mol %, an almost quantitative formation of **1b** was achieved (Table 2, entries 1 and 2). Further lowering the catalyst loading to 0.3 and 0.1 mol % resulted in 73 and 1% yield, respectively (Table 2, entries 3–4). Decreasing the reaction temperature from 60 to 45 °C and 35 °C afforded **1b** in 97 and 90% yield (Table 2, entries 5–6). The performance of MeOH was compared to EtOH and *i*PrOH, leading to lower yields (Table 2, entries 7–8). It is worth mentioning that the typical byproducts from the dehydrogenation of EtOH and *i*PrOH such as AcOEt and acetone were not observed, and thus, a transfer hydrogenation process can be ruled out. Instead, the corresponding signals of [B(OMe)_4_]^−^ as a result of quantitative alcoholysis of KBH_4_ with MeOH were detected by ^11^B NMR spectroscopy (see the Supporting Information, Figure S2).^29^ Furthermore, no conversion was observed in tetrahydrofuran (THF), highlighting the crucial role of an alcoholic solvent (Table 2, entry 9). The addition of water resulted in no significant change in conversion and selectivity (Table 2, entry 10). In the absence of the catalyst, no reaction takes place (Table 2, entry 11). Gas chromatography (GC) monitoring of the reaction revealed full consumption of **1a** within 3 h (a kinetic profile is provided in the Supporting Information, Figure S1). Upon reaction progress, **1c** was detected as the intermediate (ca. 10%), which rapidly isomerizes to the corresponding *E*-alkene (**1b**). There was no evidence of over-reduction to afford the corresponding alkane after 20 h.

**Table 2:**
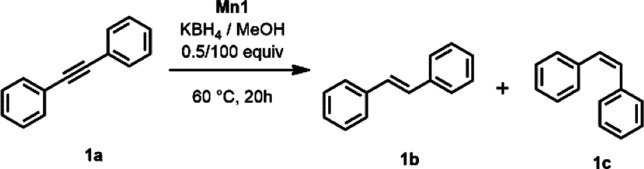
Optimization Reactions for the Semihydrogenation of **1a** with In Situ-Generated H_2_^a^

entry	Mn1 (mol %)	solvent	yield (%)	*E*/*Z*
1	1	MeOH	99^b^	99:1
2	0.5	MeOH	99^b^	99:1
3	0.3	MeOH	73	90:10
4	0.1	MeOH	1	66:33
5^c^	0.5	MeOH	97	99:1
6^d^	0.5	MeOH	90	97:3
7^c^	0.5	EtOH	5	60:40
8^c^	0.5	*i*PrOH	29	86:14
9	0.5	THF		
10^e^	0.5	MeOH	99	98:2
11		MeOH		

a Reaction conditions: diphenyl acetylene (**1a**) (0.9 mmol, 1 equiv), KBH_4_ (0.5 equiv), MeOH (3.7 mL, 100 equiv), 60 °C, 20 h, yields determined by GC–MS using *n*-dodecane as the internal standard.

b Isolated yields.

c 45 °C.

d 35 °C.

e Addition of H_2_O (200 μL).

Encouraged by the high activity of the semihydrogenation of **1a**, a series of aryl-aryl-substituted alkynes was investigated as substrates (Table 3). It turned out that in order to achieve high yields and good *E*/*Z* ratios, for substrates other than **1a**, the catalyst loadings had to be increased from 0.5 to 1 or 2 mol % in the case of terminal alkynes (see Supporting Information, Table S3). Likewise, when the reactions were performed at 90 °C, significantly higher yields and *E*/*Z* ratios could be achieved.

**Table 3:**
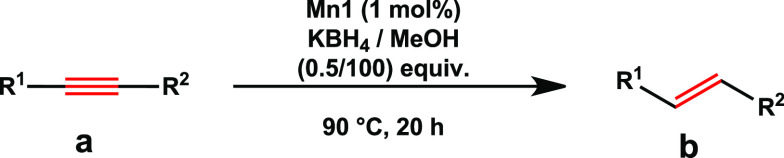

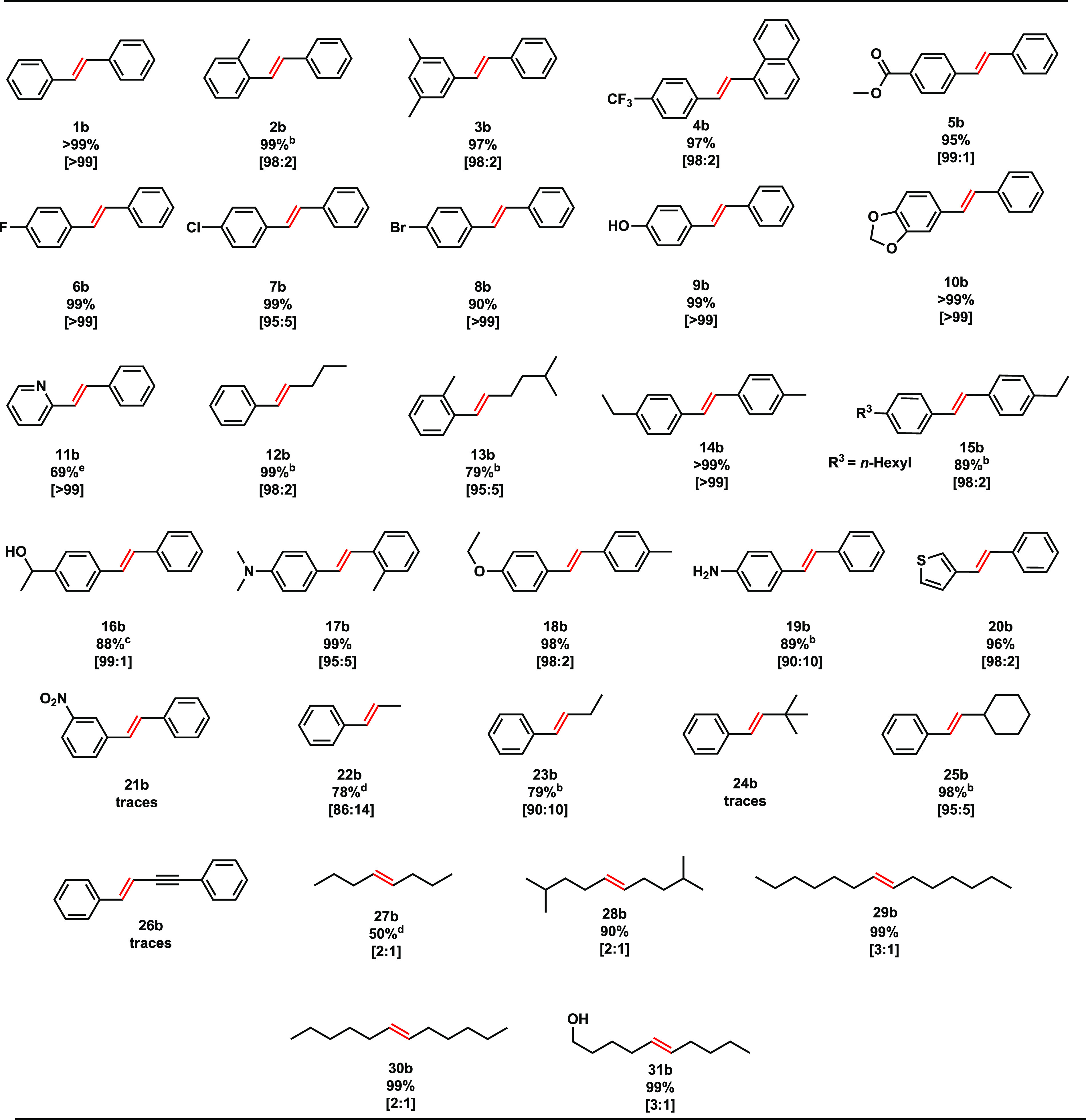
**Mn1**-Catalyzed Semihydrogenation of Alkynes with In Situ-Generated H_2_^a^

a Reaction conditions: alkyne (0.9 mmol, 1 equiv), **Mn1** (1 mol %), KBH_4_ (0.5 equiv), MeOH (3.7 mL, 100 equiv), 90 °C, 20 h, isolated yields. Value in the brackets corresponds to the *E*/*Z* ratio determined by ^1^H-NMR spectroscopy.

b **Mn1** (2 mol %).

c Substrate: (*E*)-1-(4-styrylphenyl)ethanone (**16a**), KBH_4_ (1 equiv).

d Yield determined by GC–MS using *n*-dodecane as the internal standard.

e 25h.

Under these conditions, *ortho-*, *meta-,* and *par*a-alkyl-substituted alkynes gave full conversion to *E*-stilbenes with excellent selectivity (Table 2, **1b-3b**, **14b**, and **15 b**). In the case of 1-methyl-2-(phenylethynyl)benzene (**2a**) and 1-ethyl-4-((4-hexylphenyl)ethynyl)benzene (**15a**), the catalyst loading had to be increased to 2 mol % to afford the desired *E*-alkenes in high isolated yields of 99 and 89%, respectively (Table 3, **2b** and **15b**). At 60 °C with a catalyst loading of 1 mol %, the yields of **2b** and **15b** were only 17 and 51%, respectively. Moreover, F-, Cl-, and Br-substituted substrates were tolerated, yielding corresponding *E-*products in high yields (Table 3, **6b-8b**). Notably, the ester functionality remained unaltered under the given reaction conditions, yielding the desired product with 95% yield (Table 3, **5b**). In general, substrates bearing strong electron-withdrawing groups such as CF_3_ and COOMe underwent up to 5% over hydrogenation (Table 2, **4b** and **5b**). However, the amount of overhydrogenation is much lower in comparison to hydrogenation with directly employed hydrogen (Table 1, **4b** and **5b**). Furthermore, unprotected phenol- and aniline-substituted alkynes (**9a**, **19a**) were obtained in 89 and 99% isolated yield (Table 3, **9b** and **19b**). The ketone-substituted substrate **16a** yielded *E*-1-(4-styrylphenyl)ethane-1-ol (**16b**) with 88% yield (Table 3, **16b**). More challenging substrates containing electron-donating groups such as NMe_2_ and OEt were also successfully reduced to corresponding *E-*alkenes with 98 and 99% yields and excellent *E*-selectivity (Table 3, **17b** and **18b**). In the case of 2-(phenylethynyl)pyridine (**11a**), a mixture of *E*/*Z* isomers in a 34:66 ratio was detected. Prolonging the reaction time from 20 to 25 h resulted in an increased *E*/*Z* ratio of 78:22, and **11b** was isolated in 69% yield (Table 3, **11b**). Other heterocycles containing oxygen or sulfur (**10a** and **20a**) were readily converted into the corresponding *E-*alkenes in 96 and 99% isolated yield (Table 3, **10b** and **20b**). Only traces of product were obtained in the presence of a NO_2_ group (Table 3, **21b**).

Furthermore, aryl-alkyl substituted alkynes were investigated. These substrates tend to be more challenging in selective semihydrogenations because of over-reduction and isomerization. Under the given reaction conditions, nonactivated aryl-alkyl alkynes bearing several alkyl group substituents afforded corresponding *E*-alkenes in good to excellent yields (Table 3, **12b**, **13b**, **22b**, **23b**, **25b**). For instance, **22a** gave the desired alkene with an *E*/*Z* ratio of 86:14 in 78% yield together with only 3% over-hydrogenated product (Table 2, **22b**). In the case of the sterically hindered 1-*t*butyl-2-phenylacetylene (**24a**) as well as the conjugated diyne **26a**, only traces of product were detected (Table 3, **24b** and **26b**).

Interestingly, **Mn1** was proven to be active also for the transformation of alkyl-alkyl substrate alkynes with moderate selectivity, as depicted in Table 3. Unfortunately, 4-octyne (**27a**) yielded only 50% of the desired product with an *E*/*Z* ratio of 2:1 (Table 3, **27b**). Alkynes with longer aliphatic chains proved to be more successful. 2,9-dimethyl-5-decyne (**28a**), 7-tetradecyne (**29a**), and 6-dodecyne (**30a**) were fully converted to corresponding alkenes with 90 to 99% yields (Table 3, **28b-30b**). The two isomers of 5-decenol (**31b**) are known to be hormones of the Peach Twig Borer and the Lepidoptera, respectively, and can be encountered in different industrial formulations of pesticides and fragrances.^30^**31a** was quantitatively transformed into the corresponding alkene **31b** with 99% isolated yield and a 3:1 *E*/*Z* ratio (Table 3, **31b**). Notably, double-bond migration was not observed in any case.

Terminal alkynes display another challenging class of substrates in semihydrogenation because of over hydrogenation, yielding alkanes as well as dimerization to afford 1,3-enynes.^28^ However, it was possible to achieve moderate yields of desired styrene derivatives upon increasing the catalyst loading to 2 mol % (for details, see Table S3).

The practical applicability of the system was demonstrated upon up-scaling, giving *E*-stilbene in >99% yield on a gram scale (Table 3, **1b**).

It has to be mentioned that the removal of alkyne impurities in the presence of olefins via alkyne semihydrogenation is of interest for industrial purposes because it benefits the subsequent polymerizations of some light hydrocarbon fractions from steam cracking.^2^ Because of the high selectivity of the introduced protocol, we investigated the potential applicability of this system for the purification of olefins.

**Scheme 2:**
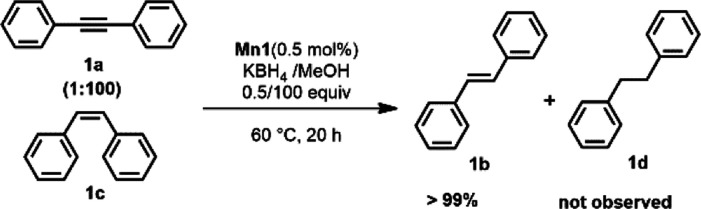
Selective Hydrogenation of **1a** and Isomerization of *Z*-Stilbene (**1c**) to *E*-Stilbene (**1b**) Catalyzed by Mn1

Accordingly, treating a mixture of **1a** and **1c** (1:100) with **Mn1** (0.5 mol %) in the presence of KBH_4_ in MeOH resulted in full conversion of the mixture to *E*-stilbene (**1b**) without the formation of over-hydrogenated product **1d** (Scheme 2).

**Scheme 3:**
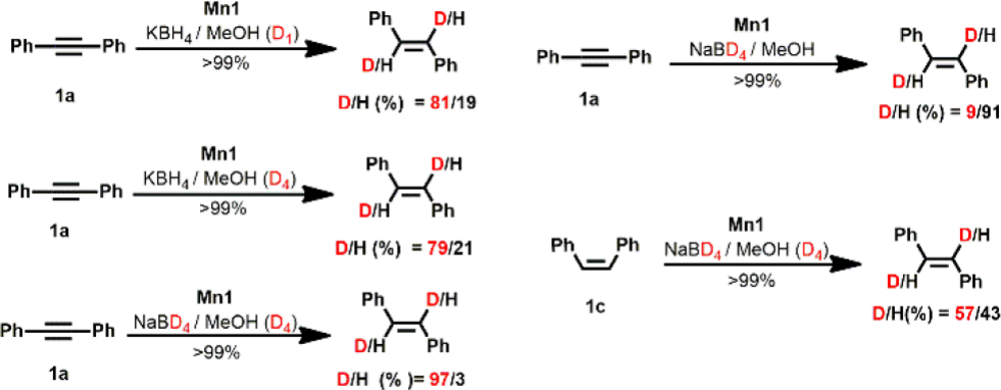
Deuterium-Labeling Studies for the *E*-Selective Semihydrogenation of **1a** and the Isomerization of **1c** Catalyzed by Mn1

Mechanistic studies were carried out to gain insights into the reaction mechanism (Scheme 3). Upon the addition of PPh_3_, only traces of *E*/*Z*-stilbene were obtained. This is attributed to the coordination of PPh_3_ to the manganese center blocking a vacant side of the catalyst. This again indicates that an inner-sphere mechanism operates in this system. Furthermore, deuterium-labeling experiments were performed. If **1a** was treated with KBH_4_ in MeOH-d_1_, a deuterium content of 81% was observed in *E*-stilbene. This amount of deuterium incorporation did not change significantly when MeOH-d_4_ was used instead. As expected, up to 97% deuterium content was incorporated when **1a** was treated with NaBD_4_ in MeOH-d_4_. In contrast, only a negligible amount of merely 9% deuterium was incorporated when NaBD_4_ was used in combination with MeOH. When *Z*-stilbene (**1c**) was treated with NaBD_4_ in MeOH-d_4_, only a deuterium content of 57% was observed in the isomerized product. These observations clearly show that the acidic proton of MeOH is almost exclusively incorporated into the substrate presumably via fast proton exchange with hydride intermediates showing the catalytic cycle (*vide infra*).^31^

**Figure 1:**
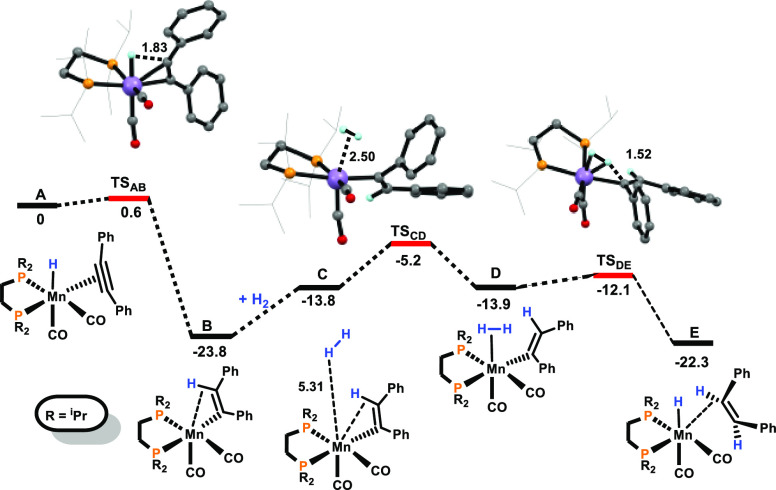
Free energy profile calculated at the PBE0/(SDD,6-31G**) level for the semihydrogenation of phenylacetylene. Free energies (kcal/mol) are referred to [Mn(dippe)(CO)_2_(H)(η^2^–PhC≡CPh)] (**A**).

**Figure 2:**
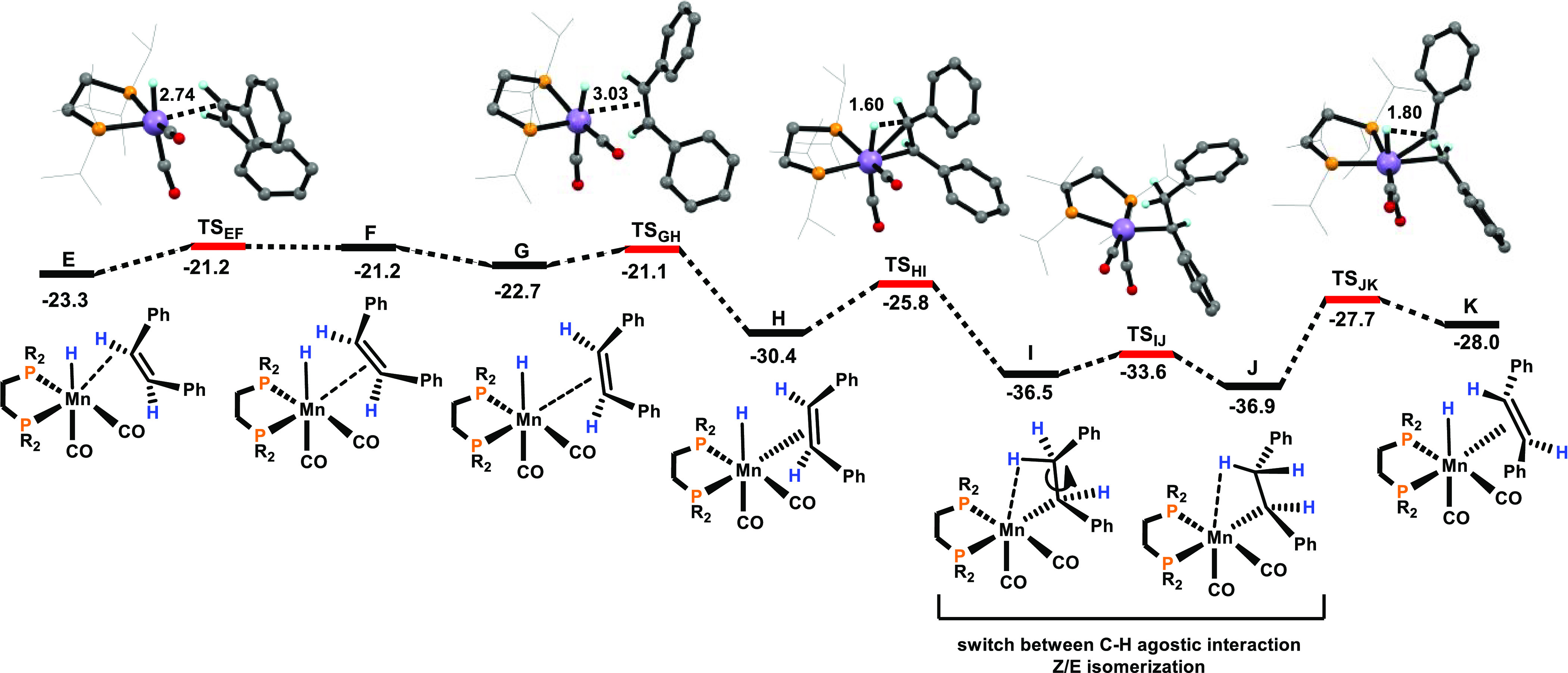
Free energy profile calculated at the PBE0/(SDD,6-31G**) level for the semihydrogenation of phenylacetylene. Free energies (kcal/mol) are referred to [Mn(dippe)(CO)_2_(H)(η^2^–PhC≡CPh)] (**A**).

**Scheme 4:**
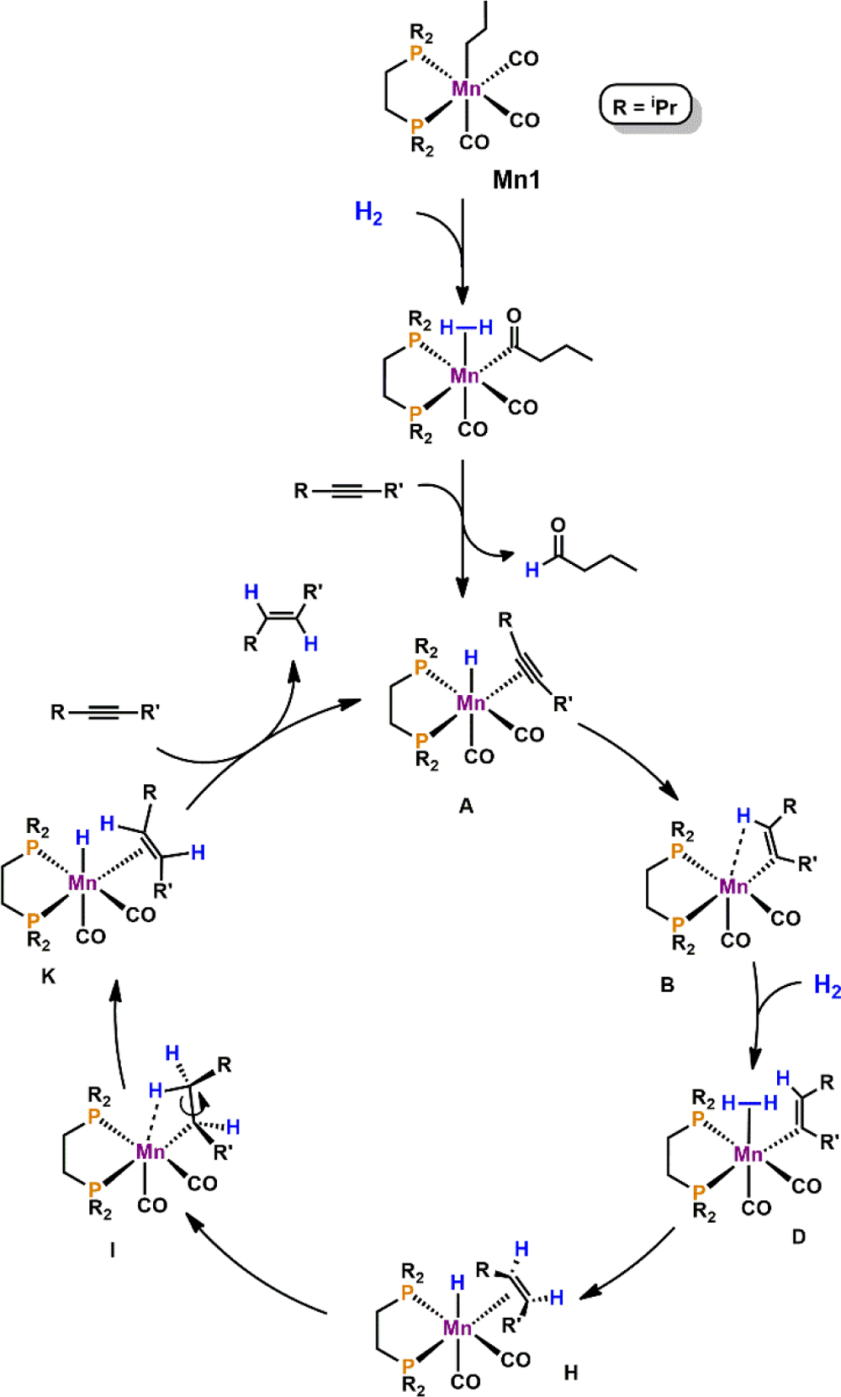
Simplified Catalytic Cycle for the *E*-Selective Semihydrogenation of Acetylenes Catalyzed by Mn1

A plausible catalytic cycle based on experimental data and density functional theory (DFT) calculations (PBE0/(SDD,6-31G**))^32^ with diphenylacetylene (**1a**) as the model substrate could be established. The resulting free energy profiles are presented in Figures 1 and 2, while Scheme 4 depicts the simplified catalytic cycle (only key intermediates are shown).

The activation of **Mn1** by dihydrogen has been reported recently.^24^ The substitution of the weakly bonded *n*-butanal by **1a** leads to the formation of the hydride species **A** featuring an η^2^-bound acetylene ligand (free energy values are referred to this complex). The insertion of acetylene into the Mn–H bond affords the coordinatively unsaturated vinyl complex **B**, which is stabilized by an agostic C–H bond. This process is exergonic by −23.8 kcal/mol with a very small barrier of 0.6 kcal/mol. The addition of dihydrogen leads via the van der Waals adduct **C** to the formation of intermediate **D**. This step is endergonic by 9.9 kcal/mol with an overall barrier of 18.6 kcal/mol being the highest of the entire cycle. The heterolytic cleavage of dihydrogen results in the formation of the hydride complex **E** featuring a C-H-bound *Z*-stilbene in an exergonic step (Δ*G* = −9.4 kcal/mol) with a negligible barrier of 1.8 kcal/mol. The reaction proceeds with the reorientation of olefin from the C–H σ-complex in **E** to the π-coordinated complex in **H**. This process proceeds via intermediates **F** and **G**, which are equivalent van der Waals pairs of *Z*-stilbene and the metallic fragment. It involves olefin dissociation, clockwise rotation of olefin by about 90°, and recoordination of olefin. The overall process has a negligible barrier of 1.2 kcal/mol and is favorable with Δ*G* = −7.1 kcal/mol, reflecting the stronger coordination of η^2^-olefin in **H** compared to the C–H σ-bound olefin in complex **E**.

In the next step of the reaction, the hydride ligand in **H** migrates to the adjacent olefin C-atom, resulting in an alkyl complex stabilized by a C–H agostic interaction in intermediate **I**. This is a facile step with a barrier of 4.6 kcal/mol and a free energy balance of Δ*G* = −6.1 kcal/mol. In **I**, a switch between the C–H agostic interaction (*Z* to *E* isomerization) yields intermediate **J** (Δ*G*^‡^ = 2.9 kcal/mol and Δ*G* = −0.4 kcal/mol) and finally β-hydrogen elimination to afford the hydride *E*-stilbene intermediate [Mn(dippe)(CO)_2_(H)(η^2^–CH(Ph)=CHPh)] (**K**) with a barrier of 9.2 kcal/mol in an endergonic final step (Δ*G* = 8.9 kcal/mol).

Closing of the catalytic cycle brings **K** back to **A** with the liberation of *E*-stilbene and the coordination of a new diphenylacetylene molecule in a favorable process with Δ*G* = −8.2 kcal/mol. Thus, the overall barrier for the reaction is 29.3 kcal/mol, measured from **J** to **TS_AB_** of the next cycle.

## Conclusions

In sum, efficient protocols for the selective semihydrogenation of aryl-aryl, aryl-alkyl, and terminal alkynes to afford *E*-alkenes are described. The precatalyst is the bench-stable alkyl complex *fac*-[Mn(dippe)(CO)_3_(CH_2_CH_2_CH_3_)]. The hydrogen gas required for the hydrogenation can be directly employed or is formed in situ upon alcoholysis of KBH_4_, wherein high-pressure equipment is not required. If hydrogen gas is directly employed (30 bar), the reaction proceeds at 60 °C with a catalyst loading of 1 mol % and notably without the addition of any additives. In case of in situ-generated hydrogen, the reaction proceeds at 90 °C with catalyst loadings of 0.5 to 2.0 mol %. These procedures represent rare examples of manganese-catalyzed hydrogenation reactions of alkynes to give selective *E*-alkenes. High functional group tolerance, including halides, phenols, nitriles, unprotected amines, and heterocycles, was observed. Even challenging substrates such as alkyl-alkyl alkynes and terminal alkynes allowed high conversions with moderate to good selectivity. The practical applicability of the protocol was demonstrated in the gram-scale synthesis of *E*-stilbene. Mechanistic investigations, including DFT calculations and deuterium-labeling studies, were undertaken to provide a reasonable reaction mechanism showing that the initially formed *Z-*isomer undergoes fast isomerization to afford the thermodynamically more stable *E-*isomer.

## Supporting Information Available

Synthetic procedures, ^1^H, ^13^C{^1^H}, ^19^F{^1^H}, and ^11^B NMR spectra of all compounds (PDF)
Cartesian coordinates for DFT-optimized structures (XYZ)

## Supplementary Material

cs1c06022_si_001.pdf

cs1c06022_si_002.xyz
