# Supplementary material for: E-Selective Manganese-Catalyzed Semihydrogenation of Alkynes with H2 Directly Employed or In Situ-Generated
Source: ACS Catal. 2022 Jan 31;12(4):2253–60. doi: 10.1021/acscatal.1c06022 (PMC8859827; doi:10.1021/acscatal.1c06022)
Supplement: Supplementary file 1 — cs1c06022_si_001.pdf [file cs1c06022_si_001.pdf]

Supporting Information

E-Selective Manganese-Catalyzed Semihydrogenation of Alkynes with H_2 - Directly Employed or *in situ* Generated

Ronald A. Farrar-Tobar,[a] Stefan Weber,[a] Zita Csendes,[a] Antonio Ammaturo,[a] Sarah Fleissner,[a] Helmuth Hoffmann,[a] Luis F. Veiros[b] and Karl Kirchner[a,*]

[a] Institute of Applied Synthetic Chemistry, Vienna University of Technology, Getreidemarkt 9, A-1060 Vienna, AUSTRIA

[b] Centro de Química Estrutural and Departamento de Engenharia Química, Instituto Superior Técnico, Universidade de Lisboa, Av Rovisco Pais, 1049-001 Lisboa, Portugal

E-mail: karl.kirchner@tuwien.ac.at

Contents

S1. General information

S2. Synthetic protocols for the semihydrogenation of alkynes

S3. Reaction monitoring of the semihydrogenation of diphenylacetylene

S4. Experiments on impact on alcoholysis

S5. Experiments on the influence of $K[B(OMe)_4]$

S6. Semihydrogenation of terminal alkynes by *in situ* generated hydrogen gas

S7. Deuterium labeling experiments

S8. Computational details

S9. Analytical data and NMR Spectra of all organic compounds:

S10. References

S1. General information

All reactions were performed under an inert atmosphere of argon by using Schlenk techniques or in a MBraun inert-gas glovebox. The solvents were purified according to standard procedures. The deuterated solvents were purchased from Aldrich and dried over activated 3 Å molecular sieves. Non-commercially available alkynes and complex *fac*-[Mn(dippe)$(CO)_3$(Pr)] (dippe = 1,2-bis(di-*iso*-propylphosphino)ethane) (**Mn1**) were synthesized according to literature.[1,2] K[B$(OMe)_4$] was synthesized from KBH_4 and MeOH. ^1H, $^{13}C\{^1H\}$, $^{19}F\{^1H\}$, $^{31}P\{^1H\}$ and ^{11}B NMR spectra were recorded on Bruker AVANCE-250 and AVANCE-400 spectrometers. ^1H and $^{13}C\{^1H\}$ NMR spectra were referenced internally to residual protio-solvent and solvent resonances, respectively, and are reported relative to tetramethylsilane (δ = 0 ppm). $^{31}P\{^1H\}$ NMR spectra were referenced externally to H_3PO_4 (85%) (δ = 0 ppm). $^{19}F\{^1H\}$ and ^{11}B NMR spectra were referenced to externally to $CFCl_3$ and $BF_3{\cdot}Et_2O$ (15%), respectively.

GC-FID analysis was carried out on a Trace 1310 with a Restek Rtx-5 15 m, 0,25 mm ID and 1 μm film thickness column using He as a carrier gas and dodecane as an internal standard. GC-MS spectra were recorded in a Thermo Scientific Ion Trap ITQ 1100, EI and CI, MS/MS, DEP (Direct Exposure Probe) for direct insertion of non-volatile samples, Trace GC Ultra with PTV, Autosampler AI/AS 3000, Column 30m (BGB-5). High-resolution accurate mass spectra were recorded on an Agilent 6545 QTOF equipped with an Agilent MMI ion source (Agilent Technologies, Santa Clara, CA, USA) which can be operated in mixed ESI and APCI mode. Measured accurate mass data for confirming calculated elemental compositions were typically within ±5 ppm accuracy. The mass calibration was performed with a commercial mixture of perfluorinated trialkyl-triazines (ES Tuning Mix, Agilent Technologies, Santa Clara, CA, USA).

S2. Synthetic protocols for the semihydrogenation of alkynes

S2.1. Semihydrogenation of alkynes by directly employed hydrogen gas

Inside a glove box, a 5 mL glass vials containing a magnetic stirrer was charged with **Mn1** (3 mg, 0.007 mmol, 1 mol%), toluene (3.5 mL) and alkyne (0.7 mmol, 1 equiv.) following this order. The vial was sealed with a septum screw cap and the septum was penetrated with a canula. The vial was placed in an aluminum carrousel of 6 slots inside a 100 mL Paar stainless steel autoclave. The autoclave was sealed, placed outside of the glovebox, purged 3 times with H_2, and charged with bar H_2 (30 bar). The autoclave was placed on a pre-heated aluminum block at indicated temperature. Vigorously stirring was applied for the indicated time. The reaction vessel was allowed to reach room temperature and carefully depressurized. *n*-Dodecane (100 µL) was added as standard and the reaction mixture was diluted with CH_2Cl_2 (2mL). A sample was taken for GC analysis, which was again diluted with CH_2Cl_2. Isolated products were received upon purification of the crude products *via* flash chromatography using *n*-pentane/Et_2O as eluent over SiO_2.

Table S1 Optimization Reactions for the Semihydrogenation of **1a** with directly employed H_2.[a]

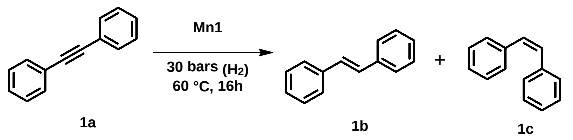

Entry	Load (mol%)	Solvent	Yield (%)	*E/Z*
**1**	1	THF	>99	99:1
**2**	1	toluene	>99	99:1
**3**[b]	1	toluene	81	95:5
**4**[b]	0.5	toluene	55	91:9
**5**	1	MeOH	60	86:14
**6**[c]	1	MeOH	13	92:8
**7**	0.5	MeOH	traces	n.d.

[a]Reaction conditions: alkyne (0.7 mmol, 1 equiv), **Mn1** (1 mol%), solvent (3.5 mL), 60 °C, 16 h: Yield and *E*/*Z* ratio determined by GC using *n*-dodecane as internal standard. [b]40°C. [c] 10 bar H_2

S2.2. Semihydrogenation of alkynes by *in situ* generated hydrogen gas

Inside a glove box, a 5 mL bushing type ace pressure tube was charged with **Mn1** (4 mg, 0.009 mmol, 1 mol%), methanol (100 equiv., 3475 µL), alkyne (0.9 mmol, 1 equiv.), (and toluene (200 µL) if required for solubility) following this order. KBH_4 (20 mg, 0.45 mmol, 0.5 equiv.) was added and the tube was sealed. The tube was removed from the glovebox, covered with aluminum foil and placed in a preheated aluminum block at the indicated temperature. After 20 h, the reaction vessel was allowed to reach room temperature, *n*-dodecane (100 µL) was added as standard and the reaction mixture was diluted with CH_2Cl_2 (2mL). A sample was taken for GC analysis, which was again diluted with CH_2Cl_2. Isolated products were received upon purification of the crude products *via* flash chromatography using *n*-pentane/Et_2O as eluent over SiO_2.

S3. Reaction monitoring of the semihydrogenation of diphenylacetylene

The hydrogenation of phenylacetylene (**1a**) was monitored in a 100 mL stainless steel Paar Autoclave equipped with an autosampler valve. Due to the larger headspace in the autoclave compared to a standard ace pressure tube, a higher amount of KBH_4 was added to reach the required H_2 pressure to accomplish full conversion of **1a**. Accordingly, the vessel was charged with **Mn1** (6 mg, 0.5 mol %), MeOH (15 mL, 150 equiv), **1a** (441 mg, 2.5 mmol), dodecane (100µL) and KBH_4 (150 mg, 1.2 equiv). The Autoclave was closed and placed outside of the glovebox in a heated aluminum block at 60 °C and samples were taken for GC analysis (Figure S1).

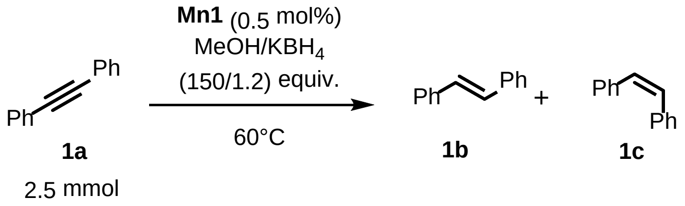

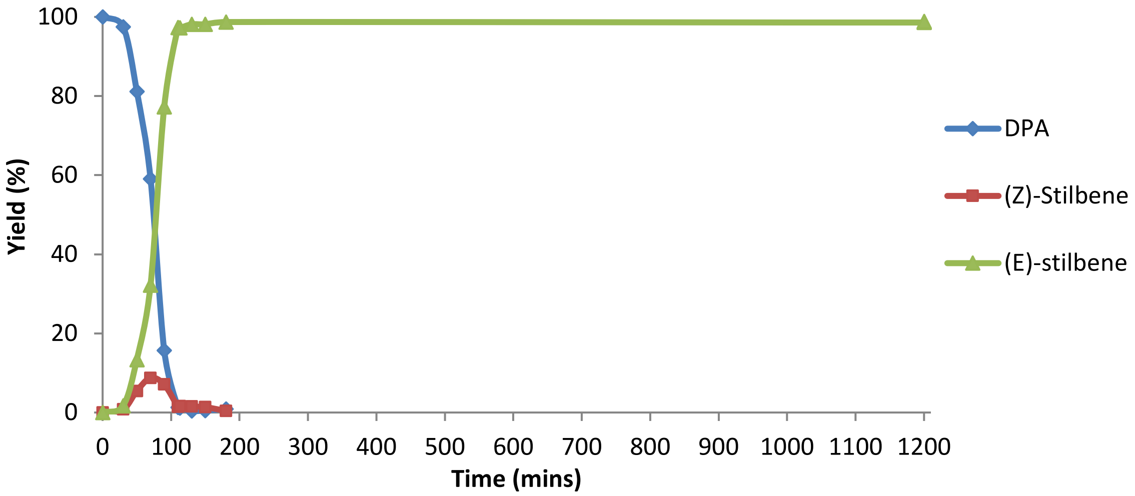

Figure S1. Kinetic profile of the semihydrogenation of (**1a**) catalysed by **Mn1** upon in situ generated H_2.

S4. Experiments on impact on alcoholysis

Inside a glove box, a 5 mL bushing type ace pressure tube was charged with **Mn1** (2 mg, 0.004 mmol), MeOH$_{d\text{-}4}$ (3400 µL), KBH_4 (18.4 mg, 0.34 mmol) and a magnetic stirring bar. The tube was sealed and stirred vigorously outside of the glove box in a pre-heated aluminum block at 60 °C. After indicated time, a sample was taken for NMR analysis. A sample without **Mn1** was prepared and analyzed under the same conditions.

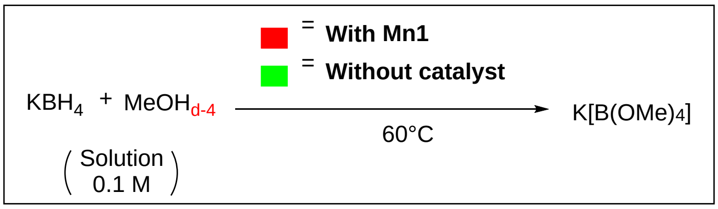

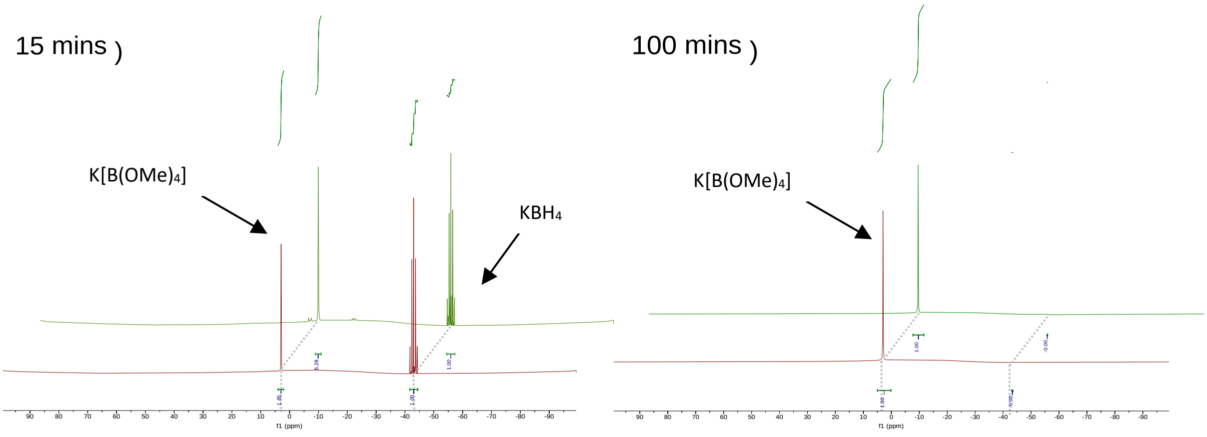

Figure S2. ^{11}B-NMR spectrum of KBH_4 alcoholysis using MeOH-d_4 with and without catalyst.

<u>Integration ratio at 15 mins [product/substrate]:</u>

- With catalyst **Mn1** [1.65/1.00]
- Without catalyst [5.28/1.00]

<u>At 100 mins. Only K[B(OMe$_3$)$_4$] peak detected.</u>

S5. Experiments on the influence of K[B(OMe)$_4$]

Inside a glove box, a 5 mL bushing type ace pressure tube was charged with **Mn1** (2 mg, 0.004 mmol, 0.5 mol%), MeOH$_{d\text{-}4}$ (100 equiv., 3475 µL), K[B(OMe)$_4$] (100 mg, 0.58 mmol, 0.7 equiv.) and diphenylacetylene (148 mg, 0.83mmol 1 equiv.), following this order. KBH_4 (18.4 mg, 0.40 mmol, 0.5 equiv.) was added and the tube was sealed. The tube was removed from the glovebox, covered with aluminum foil and placed in a preheated aluminum block at 60 °C. After 70 minutes, the reaction vessel was allowed to reach room temperature, *n*-dodecane (100 µL) was added as standard and the sample was diluted with CH_2Cl_2 (2mL). A sample was taken for ^{11}B-NMR analysis. For GC analysis, the sample was again diluted with CH_2Cl_2. A sample without K[B(OMe)$_4$] was prepared and analyzed under the same conditions.

Table S2. Optimization reactions for Semi-Reduction of Diphenylacetylene.

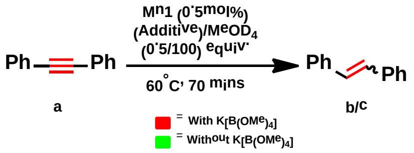

Entry	Conv.	Yield	*E:Z*
1	>99	>99	[>99]
2[b]	>99	>99	[>99]

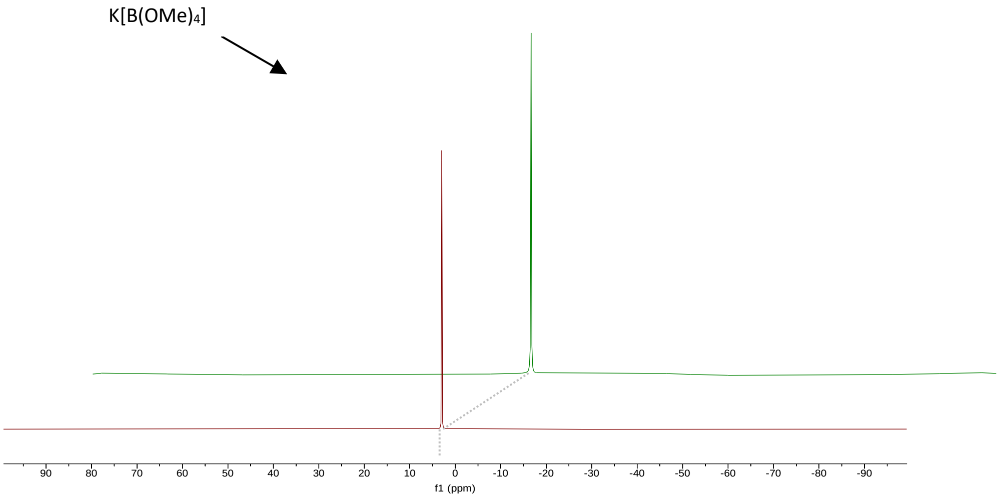

Figure S3. ^{11}B-NMR of the of the crude of reaction (Table S2) at 70 min with and without K[B(OMe)$_4$].

S6. Semihydrogenation of terminal alkynes by *in situ* generated hydrogen gas

Inside a glove box, a 5 mL bushing type ace pressure tube was charged with **Mn1** (4 mg, 0.008 mmol, 2 mol%), methanol (150 equiv., 3500 µL), alkyne (0.4 mmol, 1 equiv.), following this order. KBH_4 (6.5 mg, 0.12 mmol, 0.3 equiv.) was added and the tube was sealed. The tube was removed from the glovebox, covered with aluminum foil and placed in a preheated aluminum block at 90 °C. After 20 h, the reaction vessel was allowed to reach room temperature, *n*-dodecane (100 µL) was added as standard and the sample was diluted with CH_2Cl_2 (2mL). A sample was taken for GC analysis, which was again diluted with CH_2Cl_2.

Table S3. Mn1 catalyzed semihydrogenation of terminal alkynes by *in situ* generated hydrogen gas. [a]

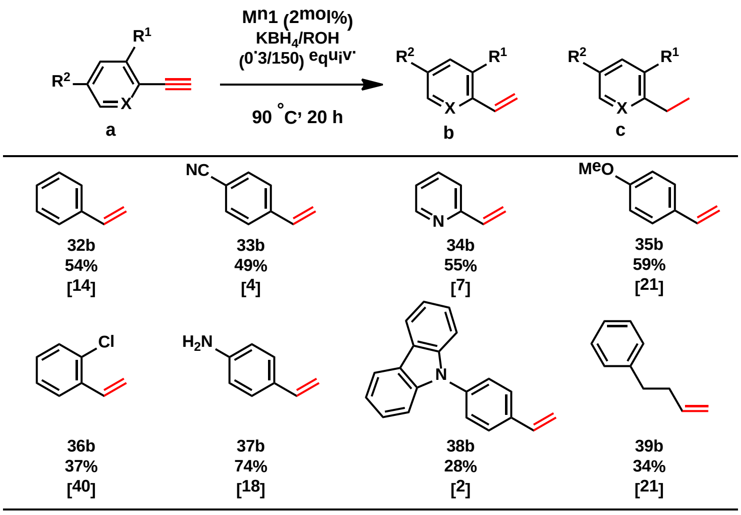

[a]Reaction conditions: alkyne (0.4 mmol, 1 equiv), **Mn1** (2 mol%), KBH_4 (0.3 equiv), MeOH (3.5 mL, 150 equiv), 90 °C, 20 h, yield corresponds to alkene (**b**), values in brackets correspond to yield of saturated alkane (**c**) as determined GC-MS using *n*-dodecane as internal standard.

S7. Deuterium labeling experiments

Labeling experiments were carried in accordance to procedure **S2.2** using $NaBD_4$ (instead of KBH_4), MeOH$_{d\text{-}1}$ (instead of MeOH) or MeOH$_{d\text{-}4}$ (instead of MeOH). For further details see Figure **S4-S8**.

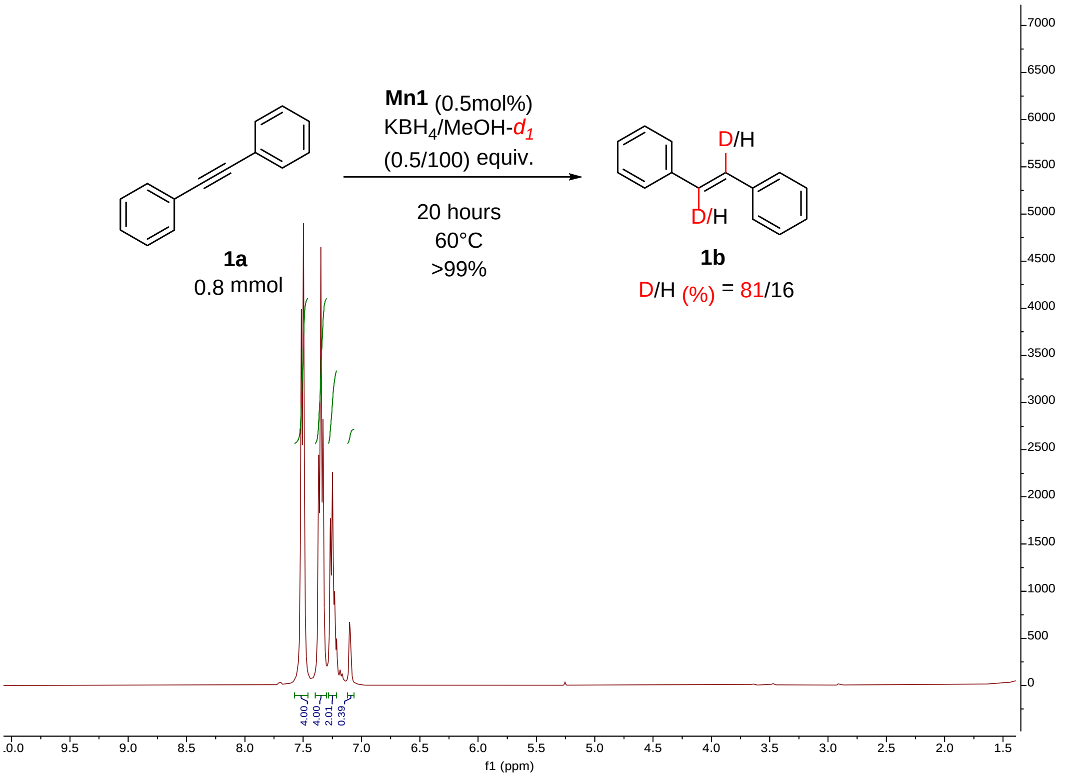

Figure S4. Mn1 catalysed semihydrogenation of **1a** with KBH_4 in MeOH-*d*$_1$.

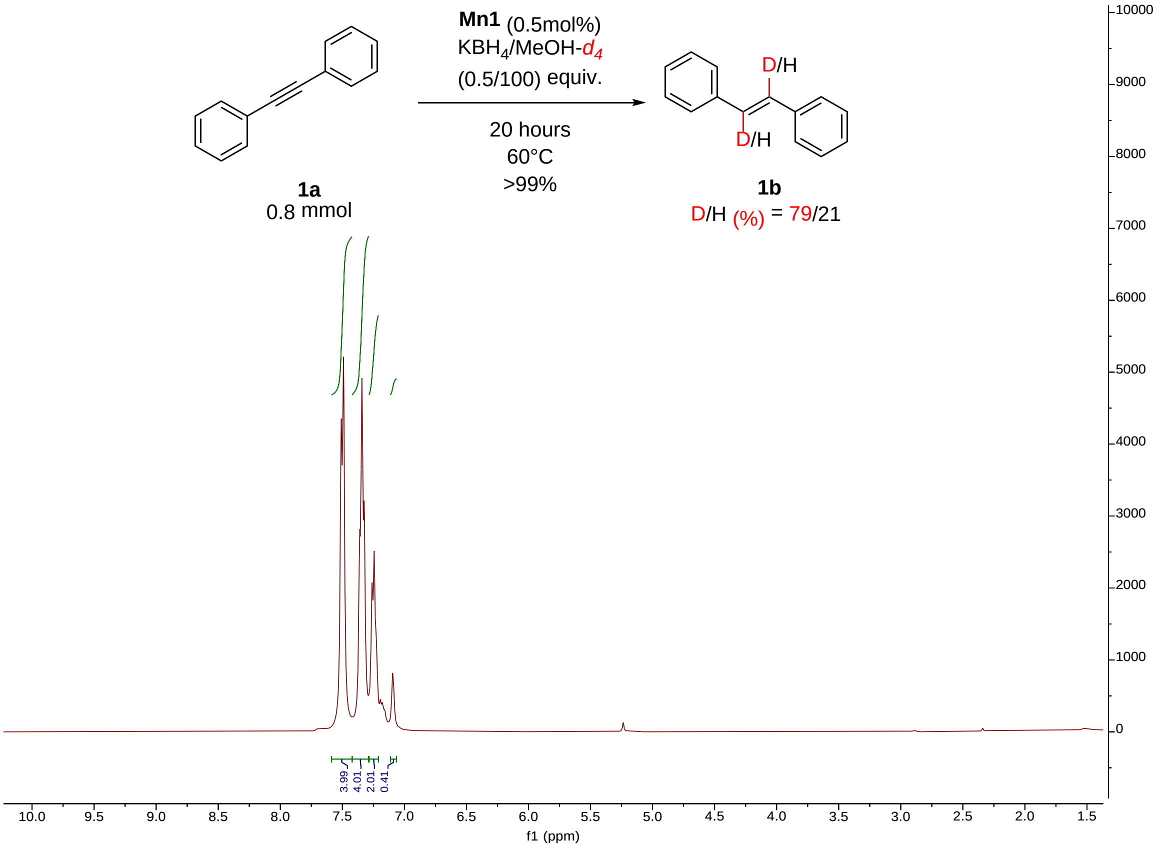

Figure S5. Mn1 catalysed semihydrogenation of **1a** with KBH_4 in MeOH-d_4.

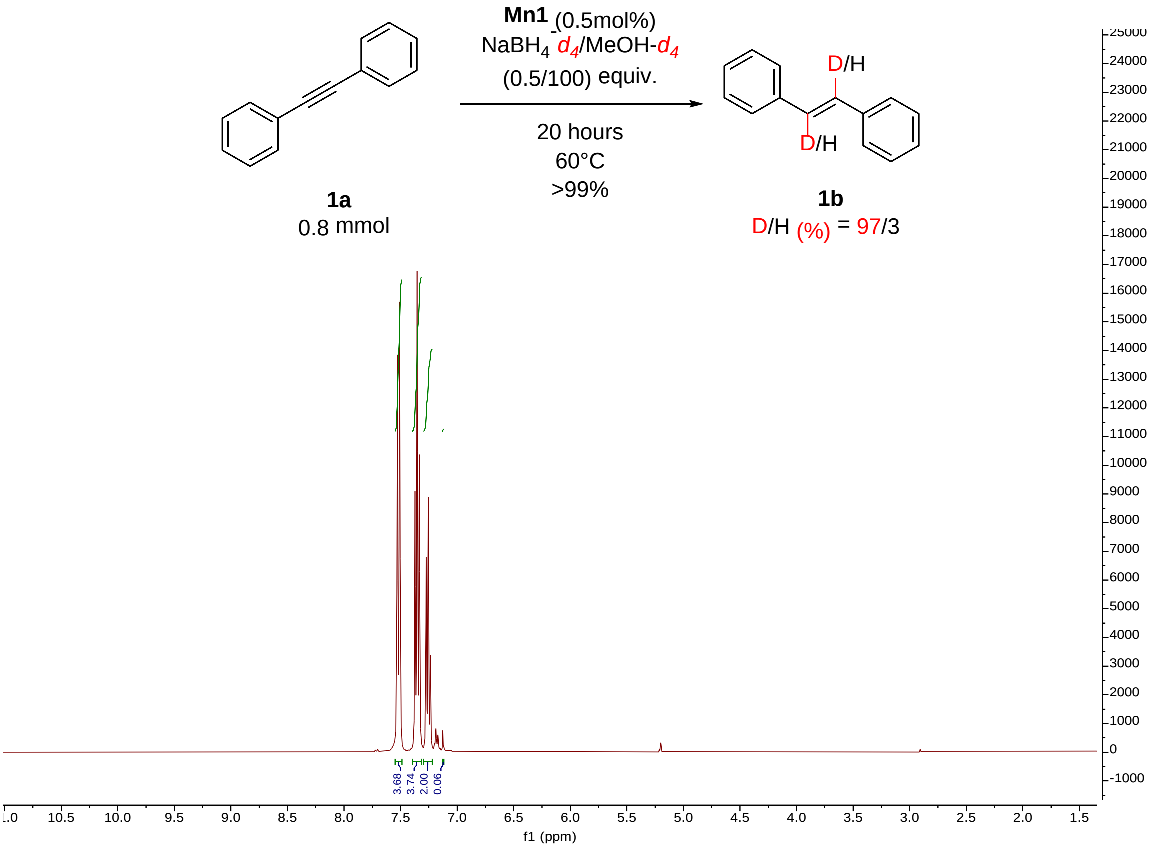

Figure S6. Mn1 catalysed semihydrogenation of **1a** with $NaBH_4$-d_4 in MeOH-d_4.

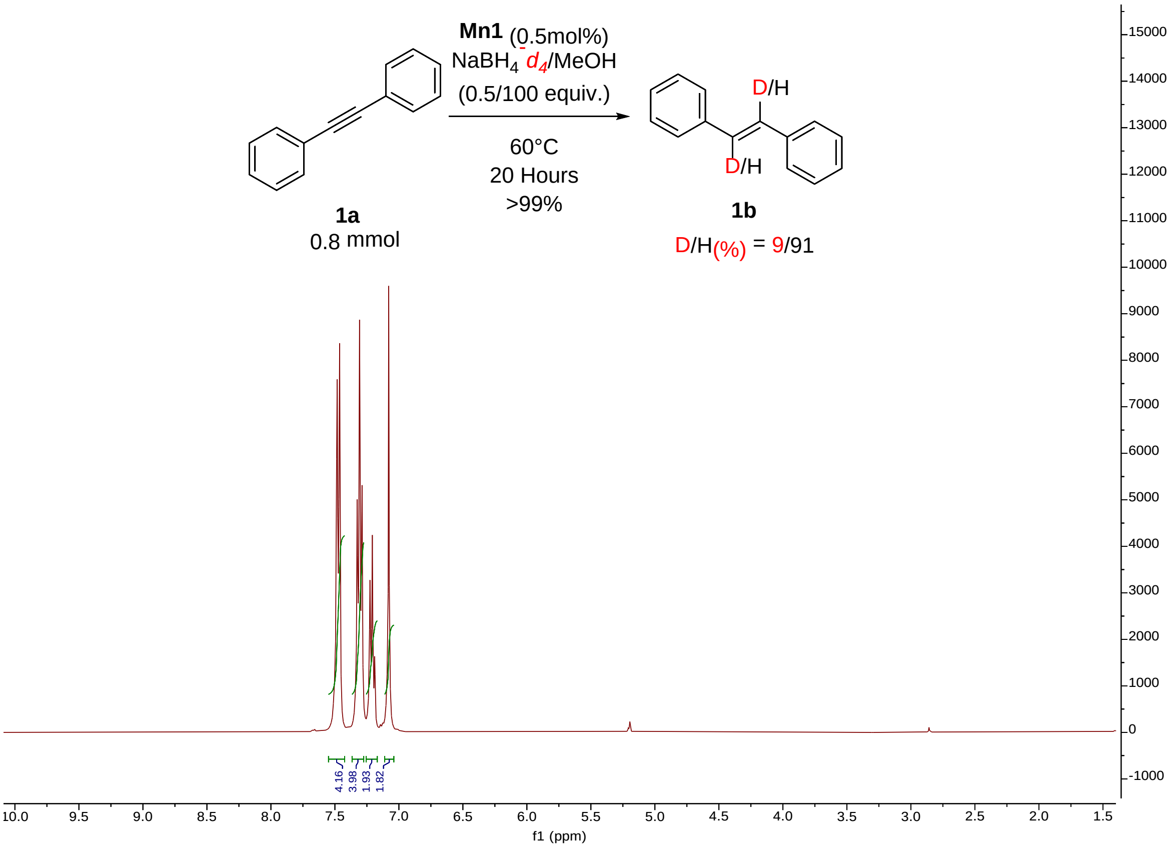

Figure S7. Mn1 catalysed semihydrogenation of **1a** with $NaBH_4$-*d_4* in MeOH.

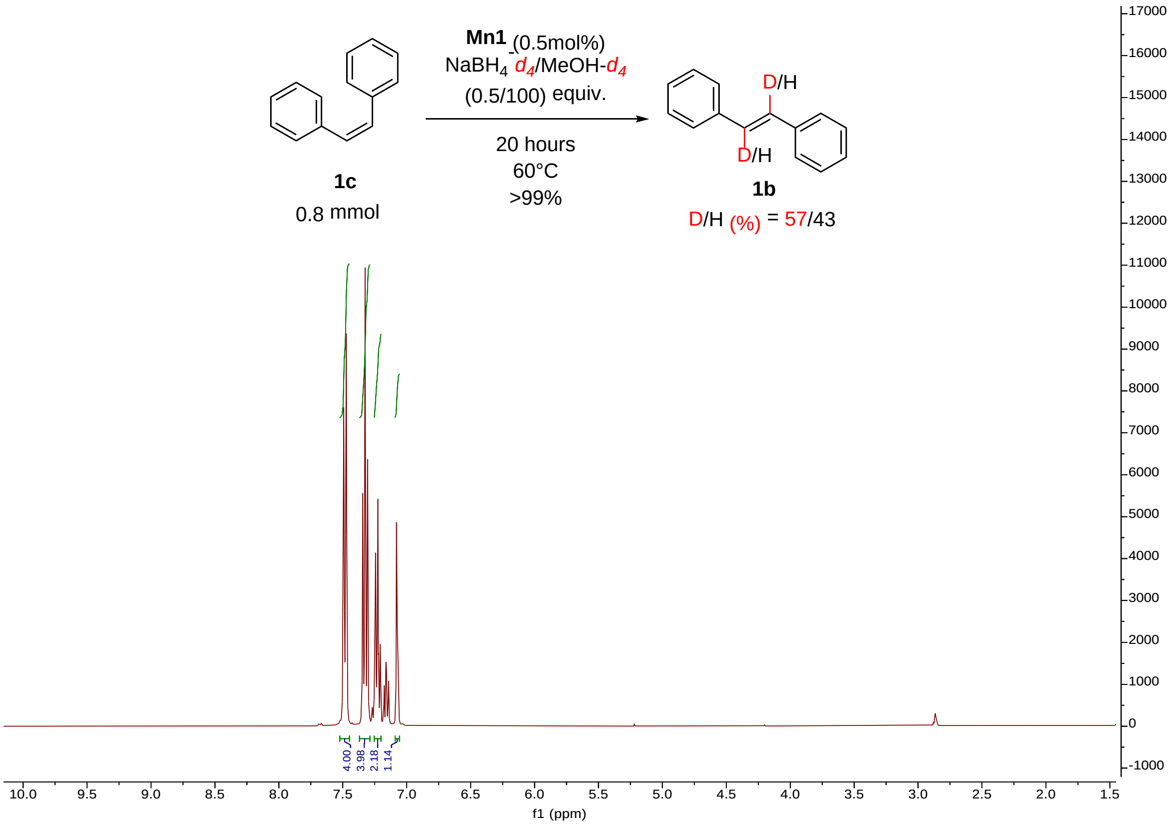

Figure S8. Mn1 catalysed isomerization of **1c** to **1b** with $NaBH_4$-*d_4* in MeOH-*d_4*.

S8. Computational details

The computational results presented have been achieved in part using the Vienna Scientific Cluster (VSC). All calculations were performed using the GAUSSIAN 09 software package.[3] Geometry optimizations were obtained using the PBE0 functional without symmetry constraints and a basis set consisting of the Stuttgart/Dresden ECP (SDD) basis set[4] to describe the electrons of Mn, and a standard 6-31G(d,p) basis set[5] for all other atoms. The PBE0 functional uses a hybrid generalized gradient approximation (GGA), including 25 % mixture of Hartree-Fock[6] exchange with DFT[7] exchange-correlation, given by Perdew, Burke and Ernzerhof functional (PBE).[8] Transition state optimizations were performed with the Synchronous Transit-Guided Quasi-Newton Method (STQN) developed by Schlegel *et al*,[9] following extensive searches of the Potential Energy Surface. Frequency calculations were performed to confirm the nature of the stationary points, yielding one imaginary frequency for the transition states and none for the minima. Each transition state was further confirmed by following its vibrational mode downhill on both sides and obtaining the minima presented on the energy profiles. The electronic energies were converted to free energy at 298.15 K and 1 atm by using zero-point energy and thermal energy corrections based on structural and vibration frequency data calculated at the same level. Solvent effects (MeOH) were considered in all the calculations using the Polarizable Continuum Model (PCM) initially devised by Tomasi and coworkers[10] with radii and non-electrostatic terms of the SMD solvation model, developed by Truhlar *et al.*[11]

S9. Analytical data and NMR spectra of all organic compounds:

Substrates:

1-methyl-2-(phenylethynyl)benzene (**2a**)[12]

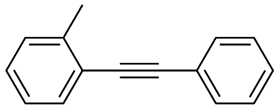

^{1}H-NMR (400 MHz, $CDCl_3$) δ 7.58 – 7.52 (m, 2H), 7.50 (dt, *J* = 7.4, 1.1 Hz, 1H), 7.42 – 7.30 (m, 3H), 7.23 (dd, *J* = 4.9, 1.2 Hz, 2H), 7.17 (dt, *J* = 8.5, 4.3 Hz, 1H), 2.52 (s, 3H).

^{13}C-NMR (101 MHz, $CDCl_3$) δ 140.33 (C), 131.98 (CH), 131.65 (CH), 129.60 (CH), 128.49 (CH), 128.44 (CH), 128.31 (CH), 125.72 (C), 123.70 (C), 123.17 (CH), 93.48 (C), 88.48 (C), 20.89 (CH_3).

1,3-dimethyl-5-(phenylethynyl)benzene (**3a**)[13]

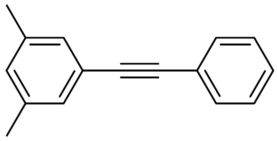

^{1}H-NMR (400 MHz, $CDCl_3$) δ 7.67 – 7.47 (m, 2H), 7.45 – 7.31 (m, 3H), 7.20 (dt, *J* = 1.5, 0.8 Hz, 2H), 6.99 (dt, *J* = 1.6, 0.8 Hz, 1H), 2.34 (d, *J* = 0.8 Hz, 6H).

^{13}C-NMR (101 MHz, $CDCl_3$) δ 138.02 (C), 131.72 (CH), 130.34 (CH), 129.44 (CH), 128.45 (CH), 128.23
(CH), 123.62 (CH), 123.01 (C), 89.88 (C), 88.84 (C), 21.26 (CH_3).

1-((4-(trifluoromethyl)phenyl)ethynyl)naphthalene (**4a**)[13]

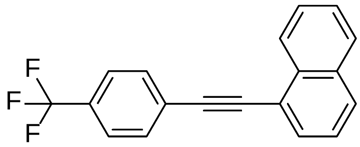

^{1}H-NMR (400 MHz, $CDCl_3$) δ 8.20 (dd, *J* = 8.3, 1.2 Hz, 1H), 7.67 (d, *J* = 7.3 Hz, 1H), 7.57 (dd, *J* = 7.1, 1.2 Hz, 1H), 7.53 (d, *J* = 8.1 Hz, 2H), 7.43 (d, *J* = 8.7 Hz, 2H), 7.40 (dd_{br}, *J* = 8.3, 1.4 Hz, 1H), 7.34 (ddd, *J* = 8.2, 6.8, 1.4 Hz, 1H), 7.26 (dd, *J* = 8.3, 7.1 Hz, 1H).

^{13}C-NMR (101 MHz, $CDCl_3$) δ 133.36 (C), 132.00 (C), 130.95 (CH), 130.17 (q, *J* = 32.8 Hz, C), 129.55 (CH), 128.58 (CH), 127.37 (q_{br}, *J* = 1.5 Hz, CH), 127.16 (CH), 126.73 (C), 126.15 (, 125.51 (q, *J* = 3.9 Hz, CF_3), 125.42, 122.77, 120.31 (C), 92.96 (C), 90.11 (C).

^{19}F-NMR (376 MHz, $CDCl_3$) δ -62.73.

methyl 4-(phenylethynyl)benzoate (**5a**)[12]

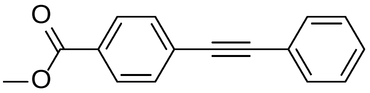

^{1}H-NMR (400 MHz, $CDCl_3$) δ 8.03 (s_{br}, 2H), 7.67 – 7.51 (m_{br}, 4H), 7.37 (s_{br}, 3H), 3.93 (s, 3H).

^{13}C-NMR (101 MHz, $CDCl_3$) δ 166.68 (COO), 131.86 (C), 131.63 (CH), 129.65 (CH), 129.60 (CH), 128.89 (CH), 128.57 (CH), 128.14 (C), 122.84 (C), 92.49 (C), 88.76 (C), 52.35 (CH_3).

1-fluoro-4-(phenylethynyl)benzene (6a)[12]

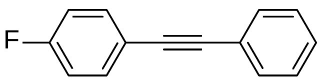

^{1}H-NMR (400 MHz, $CDCl_3$) δ 7.54 (m, 4H), 7.36 (m, 3H), 7.06 (t, J = 8.6 Hz, 2H).

^{13}C-NMR (101 MHz, $CDCl_3$) δ 162.63 (d, $J_{C\text{-}F}$ = 249.5 Hz, C), 133.61 (d, $J_{C\text{-}F}$ = 8.4 Hz, CH), 131.69 (CH), 128.51 (CH), 128.47 (CH), 123.23 (C), 119.51 (d, $J_{C\text{-}F}$ = 3.5 Hz, C), 115.77 (d, $J_{C\text{-}F}$ = 22.3 Hz, CH), 89.19 (d, $J_{C\text{-}F}$ = 1.6 Hz, C), 88.44 (C).

^{19}F-NMR (376 MHz, $CDCl_3$) δ -110.91.

1-chloro-4-(phenylethynyl)benzene (7a)[12]

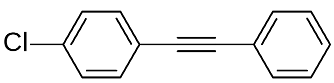

^{1}H-NMR (400 MHz, $CDCl_3$) δ 7.58 – 7.51 (m, 2H), 7.50 – 7.44 (dt, J_1=8.5, 2.0 Hz, 2H), 7.42 – 7.33 (m, 4H), 7.32 (t, J = 2. Hz, 1H).

^{13}C-NMR (101 MHz, $CDCl_3$) δ 134.40 (C), 132.95 (CH), 131.74 (CH), 128.84 (CH), 128.63 (CH), 128.54 (CH), 123.08, 121.93 (C), 90.46 (C), 88.38 (C).

4-(phenylethynyl)phenol (9a)[16]

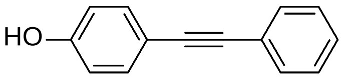

^{1}H-NMR (400 MHz, $CDCl_3$) δ 7.59 – 7.49 (m, 2H), 7.44 (dt, J=8.7, 2.0 Hz, 2H), 7.40 – 7.29 (m, 3H), 6.82 (dt, J=8.7, 2.0 Hz, 2H), 5.00 (s_{br}, 1H).

^{13}C-NMR (101 MHz, $CDCl_3$) δ 155.67 (C), 133.42 (CH), 131.59 (CH), 128.46 (CH), 128.14 (CH), 123.61 (C), 115.84 (CH), 115.65 (C), 89.34 (C), 88.25 (C).

5-(phenylethynyl)benzo[d][1,3]dioxole (10a)[19]

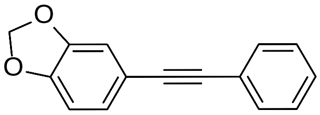

^{1}H-NMR (400 MHz, $CDCl_3$) δ 7.62 – 7.47 (m, 2H), 7.44 – 7.27 (m,3H), 7.08 (dd, J = 8.0, 1.6 Hz, 1H), 6.99 (d, J = 1.6 Hz, 1H), 6.80 (d, J = 8.0 Hz, 1H), 5.99 (s, 2H).

^{13}C-NMR (101 MHz, $CDCl_3$) δ 148.04 (C), 147.59 (C), 131.62 (CH), 128.46 (CH), 128.20 (CH), 126.40 (C), 123.51 (CH), 116.68 (C), 111.69 (CH), 108.63 (CH), 101.45 (CH_2), 89.45 (C), 87.93 (C)

2-(phenylethynyl)pyridine (11a)[17]

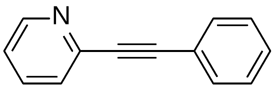

^{1}H-NMR (400 MHz, $CDCl_3$) δ 8.61 (ddd, J = 4.9, 1.8, 0.9 Hz, 1H), 7.66 (tt, J = 7.8, 1.5 Hz, 1H), 7.63 – 7.56 (m, 2H), 7.51 (dq, J = 7.8, 1.0 Hz, 1H), 7.36 (d, J = 2.3 Hz, 2H), 7.35 – 7.32 (m, 1H), 7.22 (ddt, J = 7.5, 4.9, 1.2 Hz, 1H).

^{13}C-NMR (101 MHz, $CDCl_3$) δ 150.18 (CH), 143.57 (C), 136.24 (CH), 132.14 (CH), 129.07 (CH), 128.48 (CH), 127.25 (CH), 122.84 (CH), 122.35 (C), 89.33 (C), 88.72 (C).

1-methyl-2-(5-methylhex-1-yn-1-yl)benzene (13a)

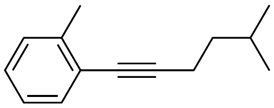

^{1}**H-NMR** (400 MHz, $CDCl_3$) δ 7.37 (d_{br}, *J* = 7.2Hz, 1H), 7.22 – 7.16 (m, 2H), 7.16 – 7.05 (m, 1H), 2.47 (t, *J* = 7.4 Hz, 2H), 2.42 (s, 3H), 1.81 (dp, *J* = 13.4, 6.7 Hz, 1H), 1.54 (q, *J* = 7.2 Hz, 2H), 0.96 (d, *J* = 6.7 Hz, 6H).

^{13}**C-NMR** (101 MHz, $CDCl_3$) δ 140.04 (C), 131.91 (CH), 129.40 (CH), 127.56 (C), 125.54 (CH), 124.01 (CH), 94.57 (C), 79.47 (C), 37.99 (CH_2), 27.41 (CH), 22.36(CH_3), 20.86 (CH_3), 17.72 (CH_2).
HR-MS MMI(+) m/z calc. for $C_{14}H_{18}$ $[M+H]^+$: 187.1481, found: 187.1486, Mass error: (+2.6 ppm)

1-(4-(phenylethynyl)phenyl)ethan-1-one (**16a**)[14]

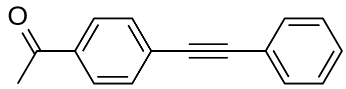

^{1}**H-NMR** (400 MHz, $CDCl_3$) δ 7.94 (dt, *J*=8.5, 2 Hz, 2H), 7.63 (dt, *J*=8.5, 2 Hz, 2H), 7.59 – 7.52 (m, 2H), 7.46 – 7.32 (m, 3H), 2.61 (s, 3H).

^{13}**C-NMR** (101 MHz, $CDCl_3$) δ 197.41 (CO), 136.31 (C), 131.87(CH), 131.82 (CH), 128.94 (CH), 128.57 (CH), 128.39 (CH), 128.31 (C), 122.77 (C), 92.84 (C), 88.74 (C), 26.74 (CH_3).

***N*,*N*-dimethyl-4-(o-tolylethynyl)aniline** (**17a**)[15]

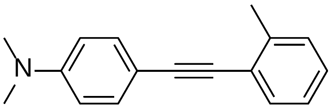

^{1}**H NMR** (400 MHz, $CDCl_3$) δ 7.49 (dd, *J* = 7.2, 1.7 Hz, 1H), 7.44 (dt, *J* = 9.0, 2.0 Hz, 2H), 7.29 – 6.85 (m, 3H), 6.58 (dt, *J* = 9.0, 2.0 Hz, 2H), 3.00 (s, 6H), 2.53 (s, 3H).

^{13}**C-NMR** (101 MHz, $CDCl_3$) δ 150.22 (C), 139.84 (C), 132.73 (CH), 131.58 (CH), 129.46 (CH), 127.62 (CH), 125.61 (C), 124.03, 112.01 (C), 110.55, 94.73 (C), 86.41 (C), 40.37 (C-N), 20.94 (CH_3).

4-(phenylethynyl)aniline (**19a**)[12]

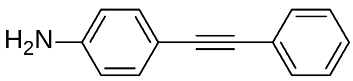

^{1}**H-NMR** (400 MHz, $CDCl_3$) δ 7.55 – 7.46 (m, 2H), 7.45 – 7.29 (m, 5H), 6.68 – 6.61 (dt,*J*=8.25, 2.0 Hz, 2H), 3.81 (s_{br}, 2H).

^{13}**C-NMR** (101 MHz, $CDCl_3$) δ 146.78 (CH, 133.08 (CH), 131.47 (CH), 128.39 (C), 127.78 (CH), 124.03 (C), 114.87 (CH), 112.72 (C), 90.25 (C), 87.45 (C).

3-(phenylethynyl)thiophene (**20a**)[18]

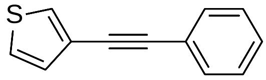

^{1}**H-NMR** (400 MHz, $CDCl_3$) δ 7.53 (ddd, *J* = 4.0, 3.4, 2.4 Hz, 3H), 7.42 – 7.33 (m, 3H), 7.31 (dd, *J* = 5.0, 3.0 Hz, 1H), 7.22 (dd, *J* = 5.0, 1.2 Hz, 1H).

13C-NMR (101 MHz, CDCl3) δ 131.67 (CH), 130.02 (CH), 128.73 (C), 128.48 (CH), 128.35 (CH), 125.50 (CH), 123.35 (C), 122.44 (CH), 89.01 (C), 84.64 (C).

1-nitro-3-(phenylethynyl)benzene (**21a**)[20]

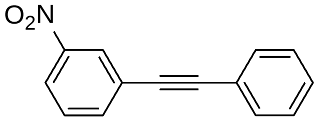

^{1}**H-NMR** (400 MHz, $CDCl_3$) δ 8.37 (ddd, *J* = 2.2, 1.5, 0.4 Hz, 1H), 8.17 (ddd, *J* = 8.3, 2.3, 1.1 Hz, 1H), 7.82 (ddd, *J* = 7.7, 1.6, 1.1 Hz, 1H), 7.70 – 7.47 (m, 3H), 7.46 – 7.34 (m, 3H).

^{13}**C-NMR** (101 MHz, $CDCl_3$) δ 148.29 (C), 137.33 (CH), 131.91 (CH), 129.48 (CH), 129.19 (CH), 128.63 (CH), 126.51, 125.28, 123.00 (C), 122.31 (C), 92.06 (C), 86.99 (C).

(3,3-dimethylbut-1-yn-1-yl)benzene (**24a**)[22]

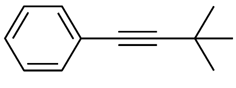

^{1}H-NMR (400 MHz, CDCl3) δ 7.40 – 7.31 (m, 2H), 7.28 – 7.18 (m, 3H), 1.59 – 1.21 (m_{br} , 9H).

^{13}C-NMR (101 MHz, $CDCl_3$) δ 131.70 (CH), 128.25 (CH), 127.52 (CH), 124.22 (C), 98.65 (C), 79.16 (C), 31.20 (CH_3), 28.07 (C).

(cyclohexylethynyl)benzene (**25a**)[21]

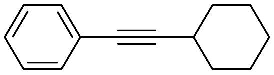

^{1}H-NMR (400 MHz, $CDCl_3$) δ 7.68 – 7.60 (m, 2H), 7.56 – 7.47 (m, 3H), 2.90 – 2.79 (m, 1H), 2.19 – 2.09 (m, 2H), 2.07 – 1.96 (m, 2H), 1.86 – 1.73 (m, 3H), 1.67 – 1.51 (m, 3H).

^{13}C-NMR (101 MHz, $CDCl_3$) δ 131.71 (CH), 128.28 (CH), 127.53 (CH), 124.28 (C), 94.60 (C), 80.65 (C), 32.87 (CH_2), 29.81 (C), 26.08 (CH_2), 25.06 (CH_2).

Products:

(E)-Stlibene (**1b**)[12]

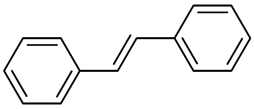

^{1}H-NMR (400 MHz, CD_2Cl_2) δ: 7.60 (d, *J* = 7.8 Hz, 5H), 7.43 (t, *J* = 7.5 Hz, 4H), 7.33 (dd, *J* = 8.4, 6.2 Hz, 2H), 7.20 (s, 2H).
^{13}C-NMR (101 MHz, $CDCl_3$) δ: 137.31(C), 128.68(CH), 128.66(CH), 127.60(CH), 126.50(CH).

(E)-1-methyl-2-styrylbenzene (**2b**)[12]

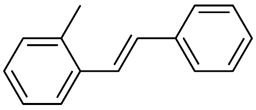

^{1}H-NMR (400 MHz, CDCl3) δ 7.62 (d, J = 7.1 Hz, 1H), 7.55 (d, *J* = 7.7 Hz, 2H), 7.38 (dd, J_1= 9.4 Hz, J_2= 7.3 Hz, 2H), 7.34 (s, 1H), 7.29 (t, *J* = 7.1 Hz, 1H), 7.27 – 7.12 (m, 3H), 7.02 (d, *J* = 16.1 Hz, 1H), 2.45 (s, 3H).
^{13}C-NMR (101 MHz, $CDCl_3$) δ 137.82, 136.54, 135.94, 130.54, 130.15, 128.82, 127.73, 127.69, 126.70, 126.34, 125.51, 20.06.

(E)-1,3-dimethyl-5-styrylbenzene (**3b**)[13]

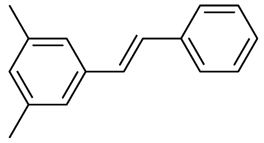

^{1}H-NMR (400 MHz, $CDCl_3$) δ 7.53 (d, *J* = 7.6 Hz, 2H), 7.38 (t, *J* = 7.5 Hz, 2H), 7.32 – 7.23 (m, 1H), 7.17 (s, 2H), 7.10 (d, *J* = 4.4 Hz, 2H), 6.94 (s, 1H), 2.37 (s, 6H).
^{13}C-NMR (101 MHz, $CDCl_3$) δ 138.25, 137.66, 137.37, 129.57, 128.79, 128.42, 127.59, 126.58, 124.57, 21.45.

(E)-1-(4-(trifluoromethyl)styryl)naphthalene (**4b**) [25]

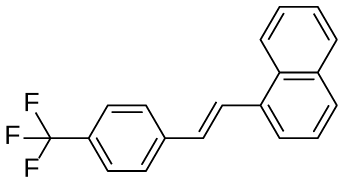

^{1}H-NMR (400 MHz, $CDCl_3$) δ 8.22 (d_{br}, *J* = 8.4, 1H), 7.98 (d, *J* = 16.0 Hz, 1H), 7.94 – 7.87 (m, 1H), 7.85 (d, *J* = 8.2 Hz, 1H), 7.77 (d_{br}, *J* = 7.2 Hz, 1H), 7.68 (q, *J* = 8.6 Hz, 4H), 7.62 – 7.46 (m, 3H), 7.17 (d, *J* = 16.0 Hz, 1H).
^{13}C-NMR (101 MHz, $CDCl_3$) δ 141.27 (d, *J* = 1.5 Hz), 134.55, 133.98, 131.60, 130.42, 129.67 (q, *J* = 32.5 Hz), 128.98, 128.92, 128.58, 127.01, 126.59, 126.24, 125.93 (q, *J* = 3.8 Hz), 125.90, 124.16, 123.81, 123.14.
^{19}F-NMR (376 MHz, $CDCl_3$) δ -62.40

methyl (*E)*-4-styrylbenzoate (**5b**)[12]

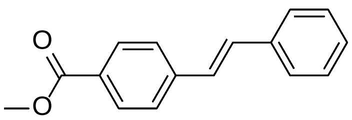

^{1}H-NMR (400 MHz, $CDCl_3$) δ 8.03 (d, *J* = 8.4 Hz, 2H), 7.72 – 7.44 (m, 4H), 7.38 (t, *J* = 7.5 Hz, 2H), 7.30 (t_{br}, *J* = 7.39 1H), 7.17 (m, J_1 = 38.0, J_2 = 16.5 Hz, 2H), 3.93 (s, 3H).
^{13}C-NMR (101 MHz, $CDCl_3$) δ 166.99, 141.93, 136.87, 131.34, 130.15, 129.03, 128.90, 128.36, 127.68, 126.91, 126.44, 52.18.

(E)-1-fluoro-4-styrylbenzene (**6b**)[12]

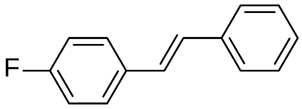

^{1}H-NMR (400 MHz, $CDCl_3$) δ 7.56 – 7.42 (m, 4H), 7.36 (t, *J* = 7.5 Hz, 2H), 7.26 (t, *J* = 7.3 Hz 1H), 7.12 – 6.95 (m, 4H).

^{13}C-NMR (101 MHz, $CDCl_3$) δ 163.71, 161.25, 137.31, 133.66 (d, *J* = 3.3 Hz), 128.85, 128.63 (d, *J* = 2.4 Hz), 128.12 (d, *J* = 8.0 Hz), 127.72 (d, *J* = 19.3 Hz), 126.58, 115.76 (d, *J* = 21.6 Hz).
^{19}F-NMR (376 MHz, $CDCl_3$) δ -114.23.

(E)-1-chloro-4-styrylbenzene (**7b**)[12]

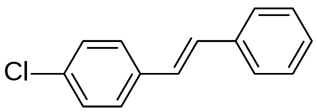

^{1}H-NMR (400 MHz, $CDCl_3$) δ 7.51 (dd, J_1 = 8.3, J_2 = 1.3 Hz, 2H), 7.45 (dt, J_1 = 8.5, J_2 = 1.9 Hz, j2H), 7.41 – 7.27 (m, 5H), 7.07 (dd J_1 = 19.3 Hz, J_2 = 16.3, 2H).

^{13}C-NMR (101 MHz, $CDCl_3$) δ 137.13, 135.99, 133.32, 129.46, 128.99, 128.88, 128.02, 127.80, 127.51, 126.69.

(E)-1-bromo-4-styrylbenzene (**8b**)[24]

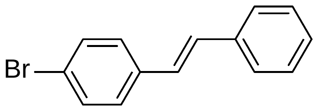

^{1}H-NMR (400 MHz, $CDCl_3$) δ 7.53 (t_{br}, *J* = 9.7 Hz, 4H), 7.39 (t_{br}, *J* = 9.4 Hz, 4H), 7.33 (t_{br}, *J* = 7.1 Hz, 1H), 7.09 (q, *J* = 16.3 Hz, 2H).

^{13}C-NMR (101 MHz, $CDCl_3$) δ 136.84, 136.16, 131.67, 129.31, 128.65, 127.89, 127.81, 127.28, 126.49, 121.21.

(E)-4-styrylphenol (**9b**)[35]

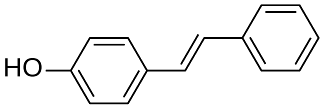

^{1}H-NMR (400 MHz, MeOD) δ 7.31 – 7.22 (m, 2H), 7.16 (d, *J* = 8.6 Hz, 2H), 7.08 (t, *J* = 7.7 Hz, 3H), 7.00 – 6.92 (m, 1H), 6.85 (d, *J* = 16.4 Hz, 1H), 6.73 (d, *J* = 16.4 Hz, 1H), 6.57 (d, *J* = 8.6 Hz, 2H), 4.69 (s, 3H).
^{13}C-NMR (101 MHz, MeOD) δ 158.38, 139.26, 130.42, 129.58, 129.52, 128.84, 127.94, 127.12, 126.73, 116.48.

(E)-5-styrylbenzo[d][1,3]dioxole (**10b**)[28]

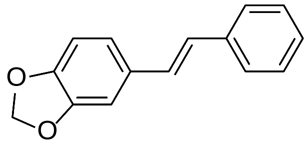

^{1}H-NMR (400 MHz, $CDCl_3$) δ 7.63 – 7.46 (m, 2H), 7.42 – 7.32 (m, 2H), 7.31 – 7.23 (m, 1H), 7.09 (d, *J* = 1.8 Hz, 1H), 7.05 (d, *J* = 16.2 Hz, 1H), 6.97 (s, 1H), 6.97 – 6.90 (m, 1H), 6.82 (d, *J* = 8.0 Hz, 1H), 5.98 (s, 2H).
^{13}C-NMR (101 MHz, $CDCl_3$) δ 148.29, 147.45, 137.54, 132.02, 128.79, 128.48, 127.49, 127.15, 126.44, 121.60, 108.54, 105.69, 101.25.

(E)-2-styrylpyridine (**11b**)[30]

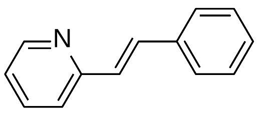

^{1}H-NMR (400 MHz, $CDCl_3$) δ 8.61 (d, *J* = 4.2 Hz, 1H), 7.76 – 7.65 (m, 1H), 7.63 (d, *J* = 3.5 Hz, 1H), 7.62 – 7.52 (m, 2H), 7.44 – 7.35 (m, 4H), 7.34 – 7.27 (m, 1H), 7.18 (d, *J* = 16.0 Hz, 1H), 7.15 – 7.10 (m, 1H).
^{13}C-NMR (101 MHz, $CDCl_3$) δ 155.75, 149.80, 136.77, 136.64, 132.83, 128.84, 128.44, 128.08, 127.22, 122.22, 122.17.

(E)-pent-1-en-1-ylbenzene (**12b**)[29]

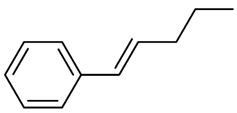

^{1}H-NMR (400 MHz, $CDCl_3$) δ 7.74 – 7.02 (m, 5H), 6.40 (d, *J* = 15.9 Hz, 1H), 6.25 (dd, *J* = 15.0, 7.6 Hz, 1H), 2.21 (q, *J* = 7.1 Hz, 2H), 1.52 (q, *J* = 7.4 Hz, 2H), 0.98 (t, *J* = 7.3 Hz, 3H).
^{13}C-NMR (101 MHz, $CDCl_3$) δ 137.97, 131.00, 129.91, 128.48, 126.77, 125.93, 35.15, 22.58, 13.76.

(E)-1-methyl-2-(5-methylhex-1-en-1-yl)benzene (**13b**)

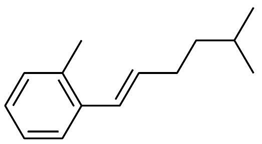

^{1}H-NMR (400 MHz, $CDCl_3$) δ 7.44 (d, *J* = 6.9 Hz, 1H), 7.23 – 7.02 (m, 3H), 6.60 (d, *J* = 15.6 Hz, 1H), 6.12 (dt, *J* = 15.6, 6.9 Hz, 1H), 2.36 (s, 3H), 2.31 – 2.20 (m, 2H), 1.67 (dp, *J* = 13.3, 6.7 Hz,1), 1.48 – 1.32 (m, 2H), 0.96 (d, *J* = 6.7 Hz, 6H).
^{13}C-NMR (101 MHz, $CDCl_3$) δ 137.24, 134.99, 132.84, 130.26, 127.55, 126.84, 126.13, 125.57, 38.78, 31.38, 27.70, 22.67, 19.98.
HR-MS MMI(+) m/z calc. for $C_{14}H_{20}$ $[M+H]^+$: 189.1637, found: 189.1631, Mass error: (-3.2 ppm)
(*E)*-1-ethyl-4-(4-methylstyryl) benzene (**14b**)[23]

^{1}H-NMR (400 MHz, $CDCl_3$) δ: 7.47 – 7.38 (tp, J_1 = 8.2, J_2 = 1.72 4H), 7.18 (dd, J_1 = 10.5, J_2 = 7.8 Hz, 4H), 7.05 (s, 2H), 2.66 (q, *J* = 7.6 Hz, 2H), 2.37 (s, 3H), 1.26 (t, *J* = 7.6 Hz, 3H).

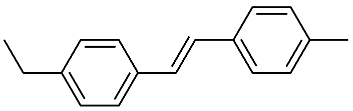

^{13}C-NMR (101 MHz, $CDCl_3$) δ: 143.68 (C), 137.25 (C), 134.99 (C), 134.74 (C), 129.35 (CH), 128.16 (CH), 127.69 (CH), 127.64 (CH), 126.37 (CH), 126.30 (CH), 28.62 (CH_2), 21.22 (CH_3), 15.53(CH_3).

(E)-1-ethyl-4-(4-hexylstyryl)benzene (**15b**)

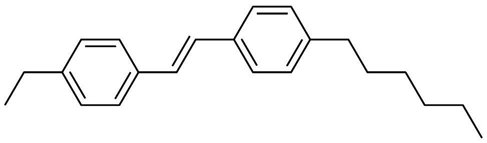

^{1}H-NMR (400 MHz, $CDCl_3$) δ 7.45 (dd, J_1 = 8.2, J_2 = 3.7 Hz, 1H), 7.20 (t, *J* = 8.6 Hz, 1H), 7.07 (s, 1H), 2.66 (m, 4H), 1.64 (q_{br}, *J* = 7.0 Hz, 2H), 1.47 – 1.30 (m, 6H), 1.27 (t, *J* = 7.6 Hz, 3H), 0.91 (td, J = 5.8, 4.8, 1.7 Hz, 1H).
^{13}C-NMR (101 MHz, $CDCl_3$) δ 143.81, 142.56, 135.18, 135.11, 128.86, 128.31, 127.90, 127.82, 126.54, 126.46, 35.88, 31.89, 31.56, 29.14, 28.78, 22.77, 15.69, 14.25.
HR-MS MMI(+) m/z calc. for $C_{22}H_{28}$ $[M+H]^+$: 293.2263, found: 293.2251, Mass error: (-4 ppm)

(E)-1-(4-styrylphenyl)ethan-1-ol (rac) (**16b**) [31]

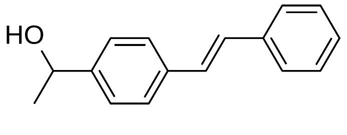

^{1}H-NMR (400 MHz, $CDCl_3$) δ 7.60 – 7.49 (m, 4H), 7.38 (t_{br}, *J* = 7.36 , 4H), 7.33 – 7.21 (tt, J_1 = 6.85, J_2 = 1.3, 1H), 7.12 (s, 2H), 4.90 (q, *J* = 6.5 Hz, 1H), 2.04 (s, 1H), 1.52 (d, *J* = 6.5 Hz, 3H).
^{13}C-NMR (101 MHz, $CDCl_3$) δ 145.34, 137.40, 136.70, 128.80, 128.72, 128.38, 127.73, 126.74, 126.61, 125.88, 70.25, 25.19.

(E)-*N*,*N*-dimethyl-4-(2-methylstyryl)aniline (**17b**)[26]

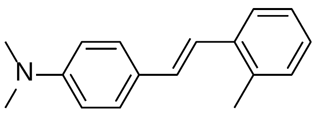

^{1}H-NMR (400 MHz, $CDCl_3$) δ 7.62 (d, *J* = 8.2 Hz, 1H), 7.46 (d, *J* = 8.8 Hz, 2H), 7.26 – 7.10 (m, 4H), 6.98 (d, *J* = 16.1 Hz, 1H), 6.76 (d, *J* = 8.8 Hz, 2H), 3.01 (s, 6H), 2.46 (s, 3H).
^{13}C-NMR (101 MHz, $CDCl_3$) δ 150.27, 137.26, 135.40, 130.41, 130.16, 127.72, 126.81, 126.33, 126.23, 125.03, 122.35, 112.60, 40.61, 20.11.

(E)-1-ethoxy-4-(4-methylstyryl)benzene (**18b**)[27]

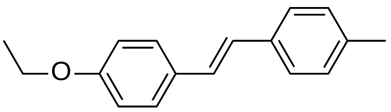

^{1}H-NMR (400 MHz, $CDCl_3$) δ 7.46 (d, *J* = 8.8 Hz, 2H), 7.43 (d, *J* = 8.0 Hz, 2H), 7.19 (d, *J* = 7.8 Hz, 2H), 7.02 (q, *J* = 16.3 Hz, 2H), 6.92 (d, *J* = 8.7 Hz, 2H), 4.07 (q, *J* = 7.0 Hz, 2H), 2.39 (s, 3H), 1.46 (t, *J* = 7.0 Hz, 3H).
^{13}C-NMR (101 MHz, $CDCl_3$) δ 158.49, 136.92, 134.85, 130.12, 129.30, 127.53, 127.24, 126.38, 126.11, 114.61, 63.40, 21.17, 14.79.

(E)-4-styrylaniline (**19b**)[12]

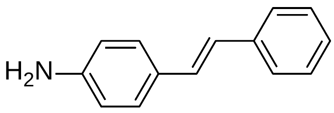

^{1}H-NMR (400 MHz, $CDCl_3$) δ 7.48 (d_{br}, *J* = 7.36 Hz 2H), 7.36 (s_{br}, 1H), 7.35 – 7.35 (m, 3H), 7.26 (tt, J_1 = 6.8, J_2 = 1.3 Hz, 1H), 7.04 (d, *J* = 16.28 Hz, 1H), 6.93 (d, *J* = 16.28 Hz, 1H), 6.68 (dt, J_1 = 8.5, J_2 = 2.0 Hz, 2H), 3.74 (s, 2H).
^{13}C-NMR (101 MHz, $CDCl_3$) δ 146.70, 138.50, 129.24, 129.15, 128.57, 128.30, 127.44, 126.65, 125.65, 115.75.

(*E*)-3-styrylthiophene (**20b**)[31]

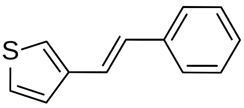

^{1}H-NMR (400 MHz, $CDCl_3$) δ 7.47 (dd, *J* = 7.5, 1.7 Hz, 2H), 7.33 (tdd, *J* = 8.0, 4.7, 2.8 Hz, 4H), 7.27 – 7.19 (m, 2H), 7.12 (d, *J* = 16.3 Hz, 1H), 6.95 (d, *J* = 16.3 Hz, 1H).
^{13}C-NMR (101 MHz, $CDCl_3$) δ 140.25, 137.51, 128.81, 127.59, 126.42, 126.32, 125.06, 123.02, 122.49.

(E)-but-1-en-1-ylbenzene (**23b**)[32]

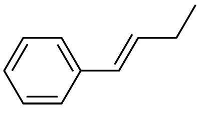

^{1}H-NMR (400 MHz, $CDCl_3$) δ 7.42 – 7.35 (m, 2H), 7.31 (dd, *J* = 8.4, 6.9 Hz, 3H), 7.25 – 7.14 (m, 1H), 6.41 (dd, *J* = 15.8, 1.4 Hz, 1H), 6.32 (t, *J* = 6.3 Hz, 1H), 2.35 – 2.21 (m, 2H), 1.12 (td, *J* = 7.4, 1.2 Hz, 3H).
^{13}C-NMR (101 MHz, $CDCl_3$) δ 138.41, 135.19, 133.09, 129.26, 129.20, 128.92, 128.71, 128.56, 127.21, 126.90, 126.37, 26.53, 22.42, 14.93, 14.11.

*(*E*)*-(2-cyclohexylvinyl)benzene (**25b**)[24]

^{1}H-NMR (400 MHz, $CDCl_3$) δ 7.37 (dt, *J* = 8.1, 1.8 Hz, 2H), 7.31 (dd, *J* = 8.5, 6.8 Hz, 2H), 7.24 – 7.14 (m, 1H), 6.37 (dd, *J* = 16.0, 1.2 Hz, 1H), 6.21 (dd, *J* = 16.0, 6.9 Hz, 1H), 2.15 (dttd, *J* = 10.4, 7.0, 3.4, 1.2 Hz, 1H), 1.95 – 1.76 (m, 4H), 1.72 (dddd, *J* = 11.3, 5.2, 3.3, 1.7 Hz, 1H), 1.46 – 1.29 (m, 2H), 1.29 – 1.10 (m, 3H).

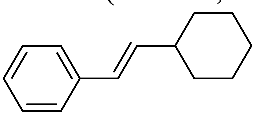

^{13}C-NMR (101 MHz, $CDCl_3$) δ 138.20, 136.98, 128.59, 127.37, 126.86, 126.08, 41.30, 33.11, 26.33, 26.20.

(E/Z)-2,9-dimethyldec-5-ene (**28a**/**28b**)[34]

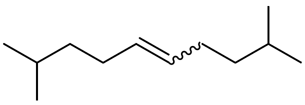

^{1}H-NMR (400 MHz, $CDCl_3$) δ 5.39 (ddt, *J* = 5.3, 3.2, 1.6 Hz, 1H), 5.33 (ddd, *J* = 5.6, 4.4, 1.1 Hz, 1H), 2.13 – 1.92 (m, 3H), 1.72 – 1.44 (m, 3H), 1.23 (dtd, *J* = 8.8, 6.7, 1.0 Hz, 5H), 0.88 (t, *J* = 6.7 Hz, 15H).
^{13}C-NMR (101 MHz, $CDCl_3$) δ 130.45, 130.00, 39.19, 39.06, 30.61, 27.78, 27.63, 25.24, 22.69, 22.67.

(*E/Z)*-tetradec-7-ene (**29b/29c**)[33]

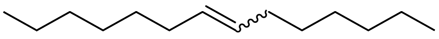

^{1}H-NMR (400 MHz, $CDCl_3$) δ 5.39 (dt, *J* = 3.2, 1.6 Hz, 0H), 5.37 – 5.30 (m, 1H), 2.37 – 1.79 (m, 3H), 1.45 – 1.07 (m, 14H), 0.88 (td, *J* = 6.9, 2.0 Hz, 5H).
^{13}C-NMR (101 MHz, $CDCl_3$) δ 130.52, 130.06, 32.77, 31.94, 31.91, 29.90, 29.78, 29.14, 28.99, 27.37, 22.81, 14.25.

(*E/Z)*-dodec-6-ene (**30b/30c**)[12]

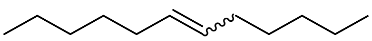

^{1}H-NMR (400 MHz, $CDCl_3$) δ 5.39 (t, *J* = 3.8 Hz, 1H), 5.36 (t, *J* = 4.8 Hz, 0H), 1.99 (dq, *J* = 11.9, 6.2 Hz, 2H), 1.69 – 1.15 (m, 6H), 0.89 (t, *J* = 6.8 Hz, 3H).
^{13}C-NMR (101 MHz, $CDCl_3$) δ 130.37, 129.90, 32.58, 31.54, 31.41, 29.46, 29.35, 27.18, 22.59, 22.56, 14.07, 14.00.

(*E/Z)*-dec-5-en-1-ol (**31b/31c**)[26/25]

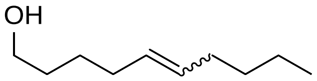

^{1}H-NMR (400 MHz, $CDCl_3$) δ 5.82 – 5.08 (m, 1H), 3.63 (dd, *J* = 7.9, 5.2 Hz, 1H), 2.59 – 1.93 (m, 2H), 1.56 (dq, *J* = 8.8, 6.5 Hz, 1H), 1.41 (qd, *J* = 7.5, 3.4 Hz, 1H), 1.30 (dq, *J* = 6.4, 3.2, 2.6 Hz, 2H), 0.88 (td, *J* = 7.1, 4.4 Hz, 2H).
^{13}C-NMR (101 MHz, $CDCl_3$) δ 131.32, 130.80, 130.23, 129.76, 63.36, 32.82, 32.73, 32.70, 32.68, 32.37, 32.23, 27.38, 27.34, 26.31, 26.16, 22.79, 22.64, 14.43, 14.39

Alkynes Spectra:

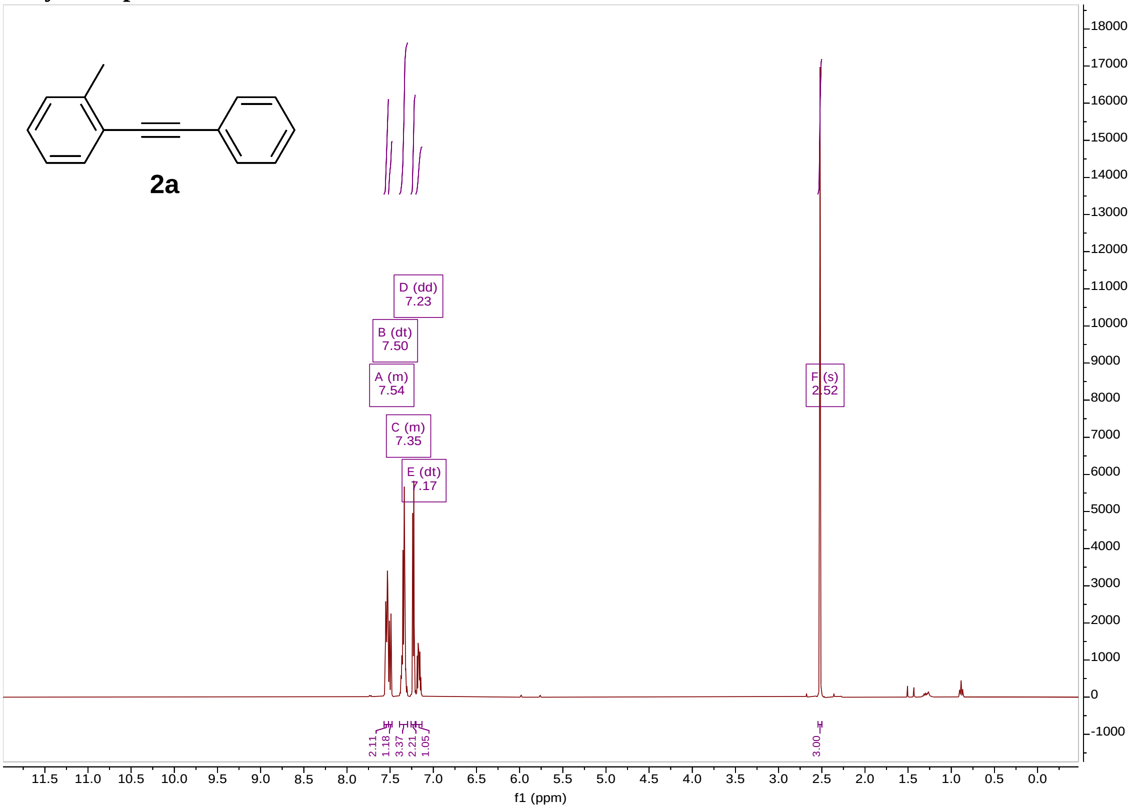

Figure S9. ^{1}H-NMR spectra of compound (**2a**).

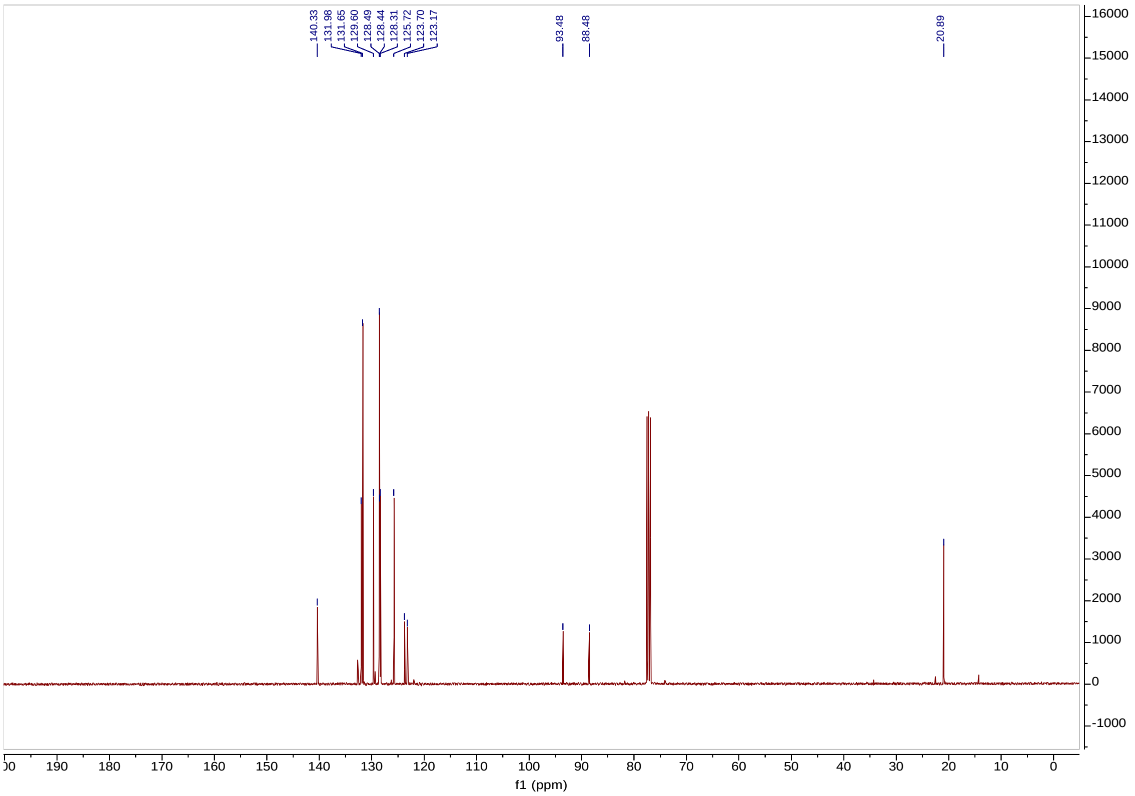

Figure S10. ^{13}C-NMR spectra of compound (**2a**).

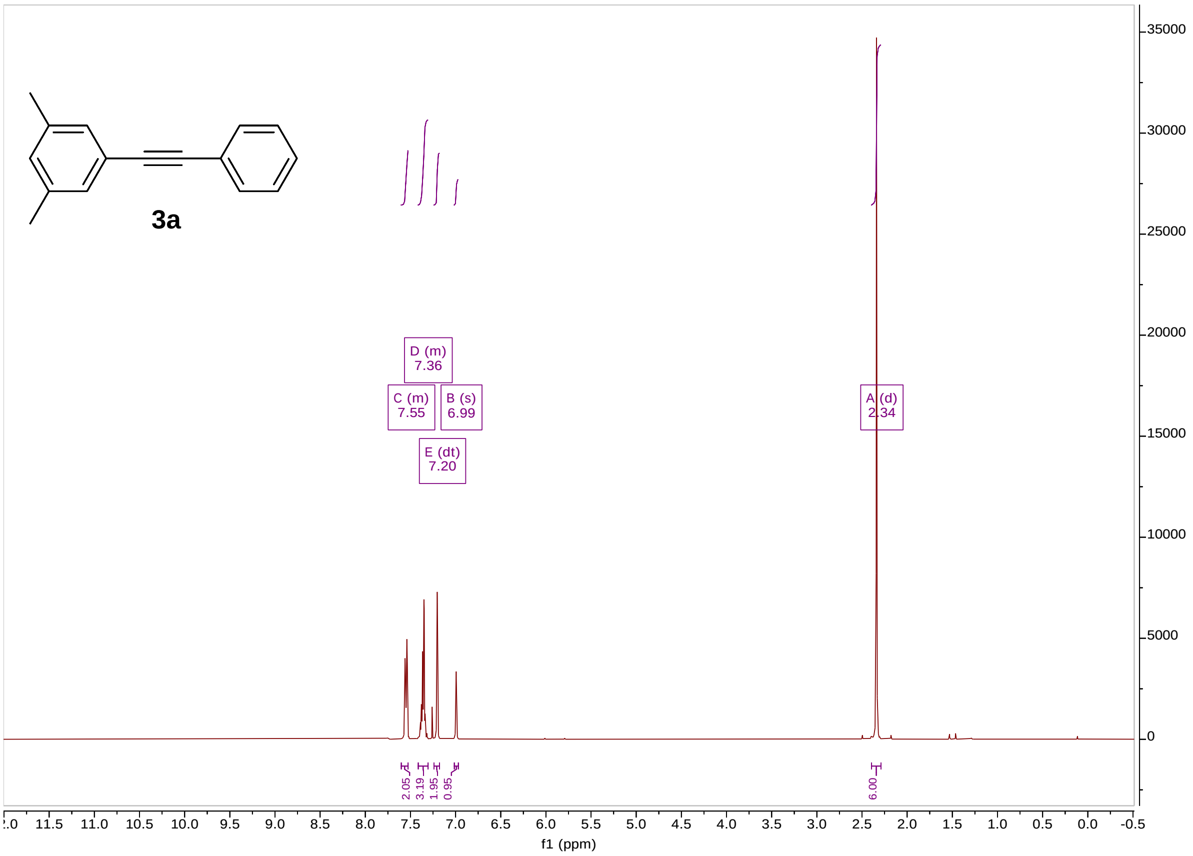

Figure S11. ^{1}H-NMR spectra of compound (**3a**).

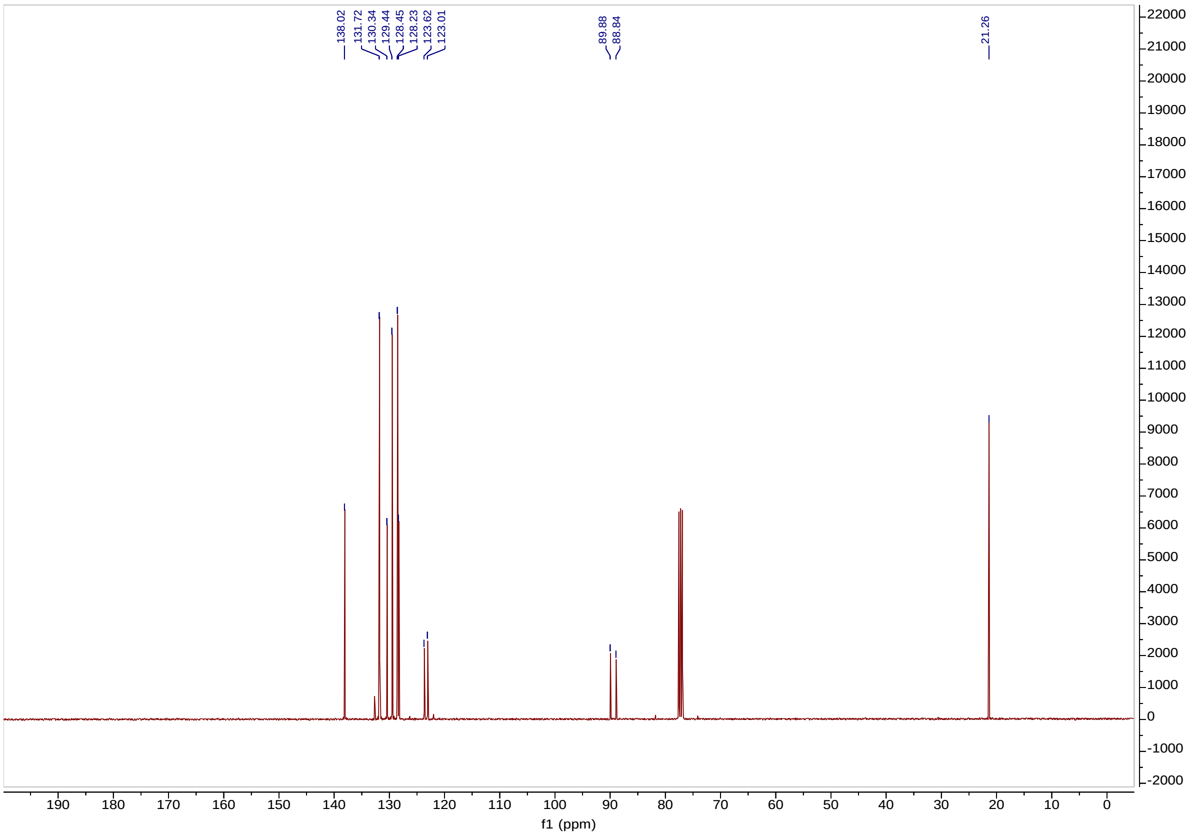

Figure S12. ^{13}C-NMR spectra of compound (**3a**).

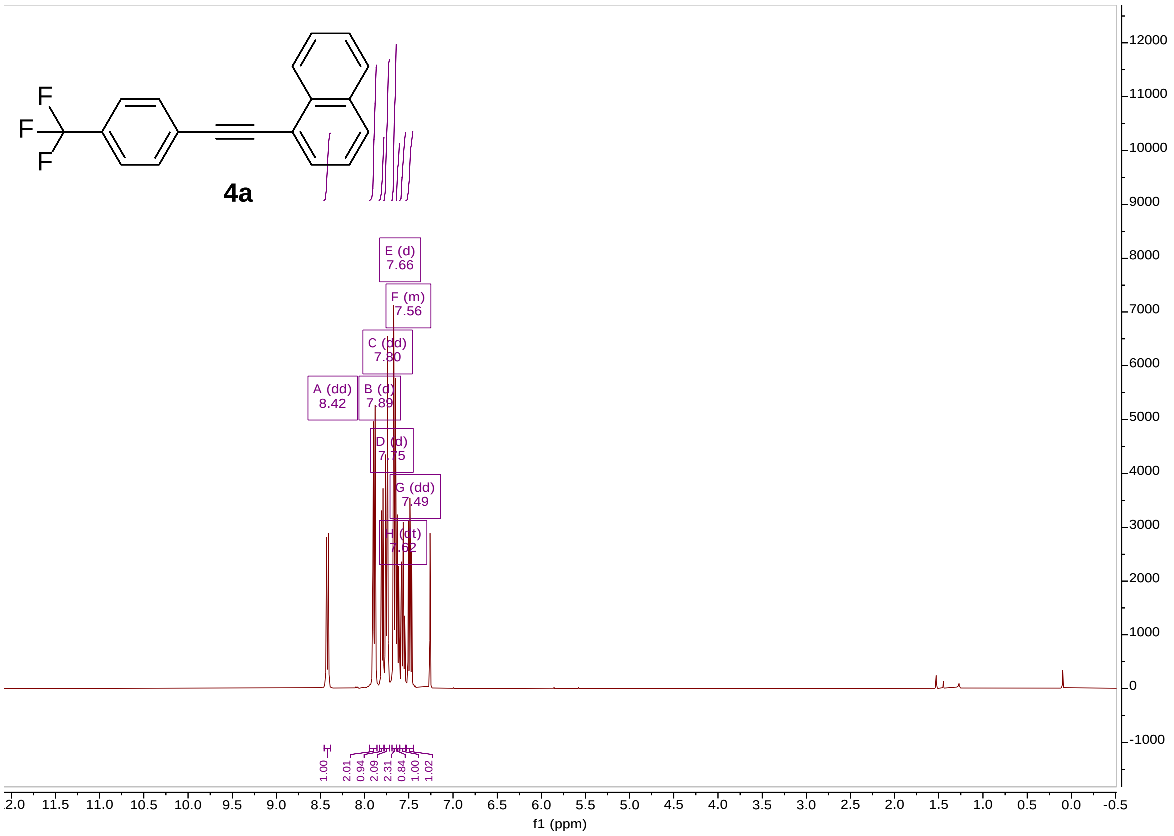

Figure S13. ^{11}H-NMR spectra of compound (**4a**).

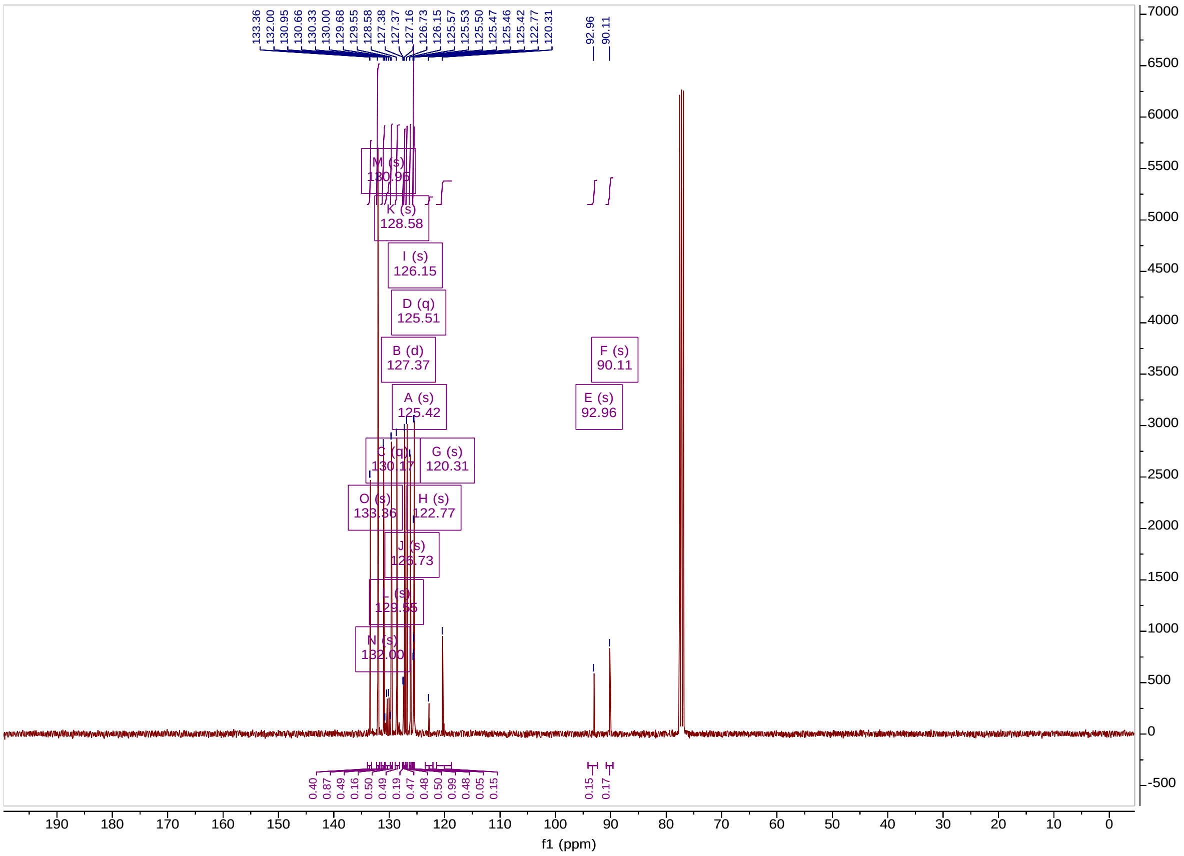

Figure S14. ^{13}C-NMR spectra of compound (**4a**).

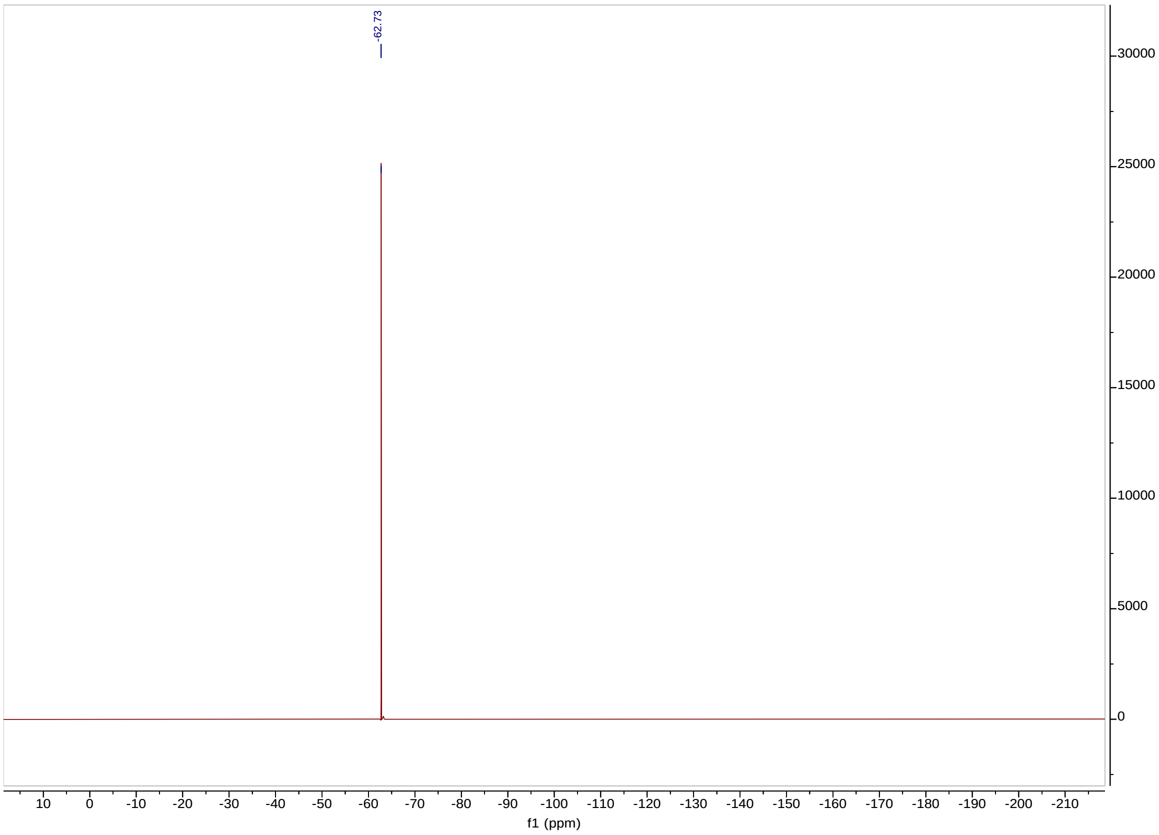

Figure S15. ^{11}F-NMR spectra of compound (**4a**).

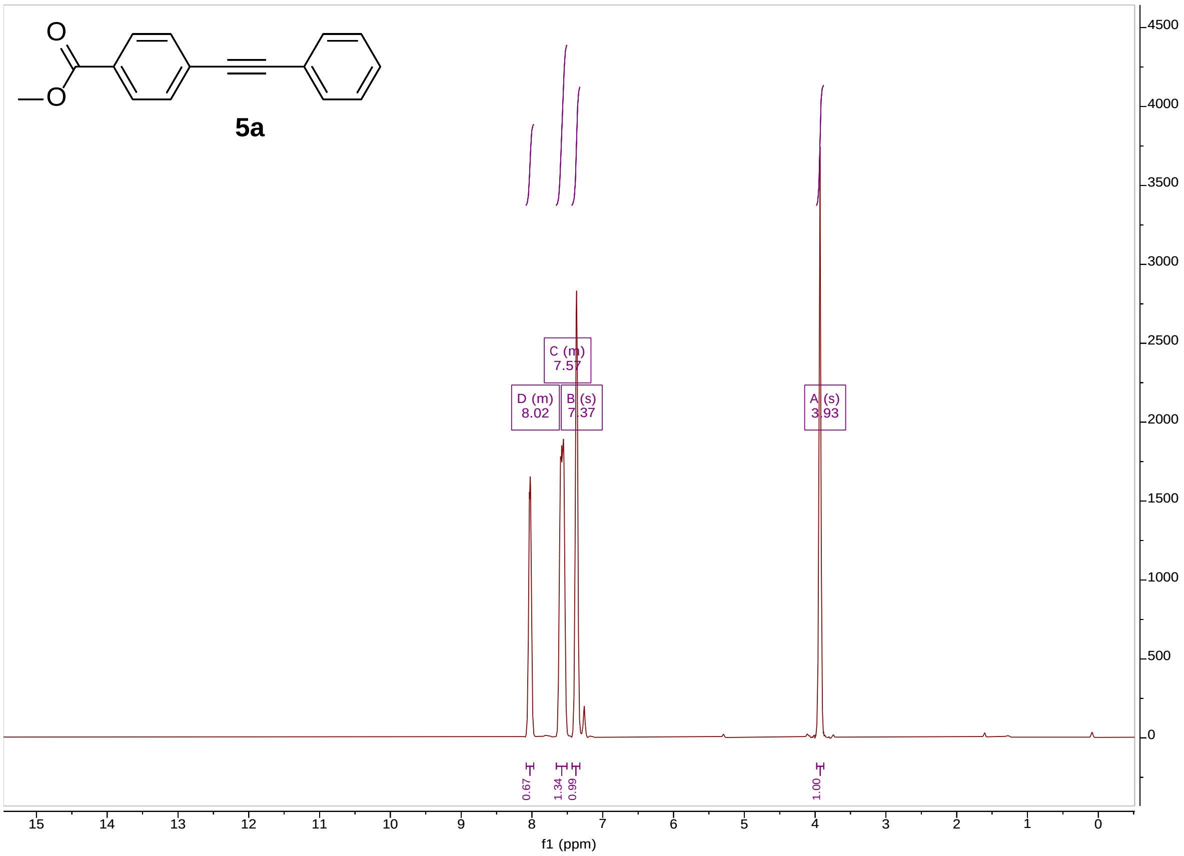

Figure S16. ^{1}H-NMR spectra of compound (**5a**).

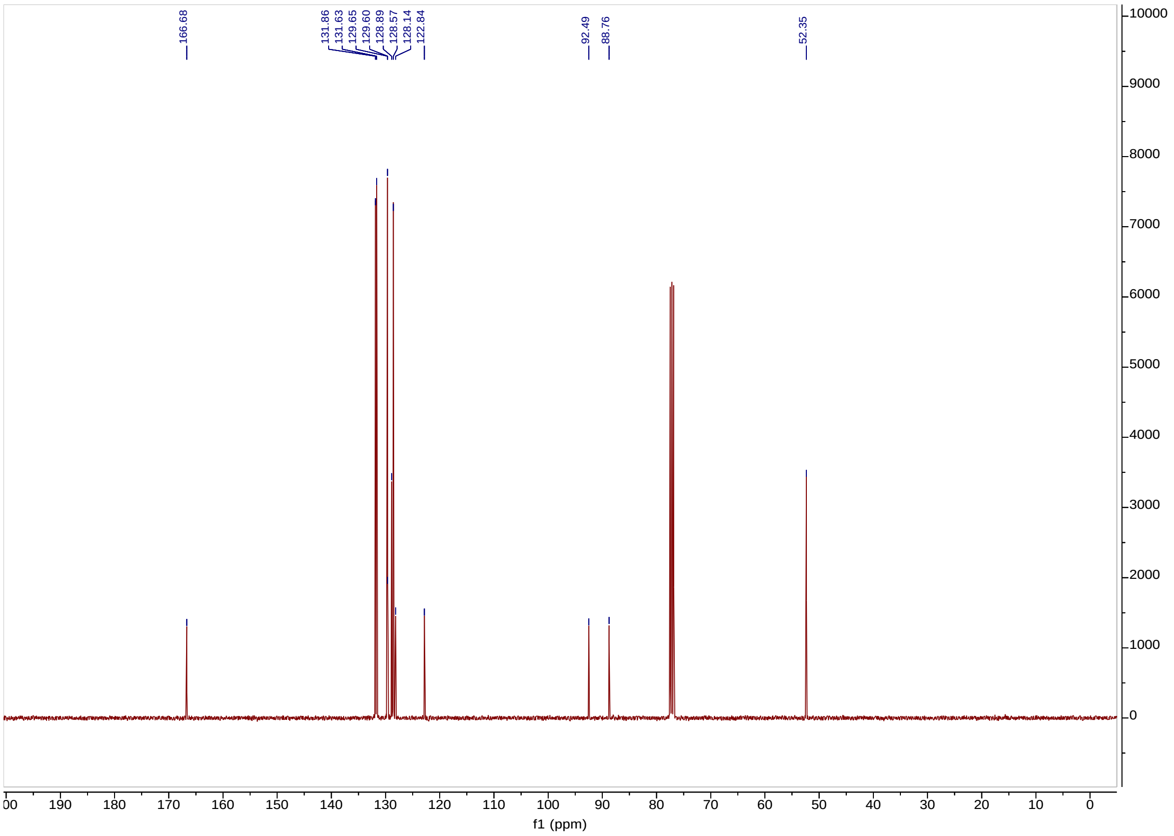

Figure S17. ^{13}C-NMR spectra of compound (**5a**).

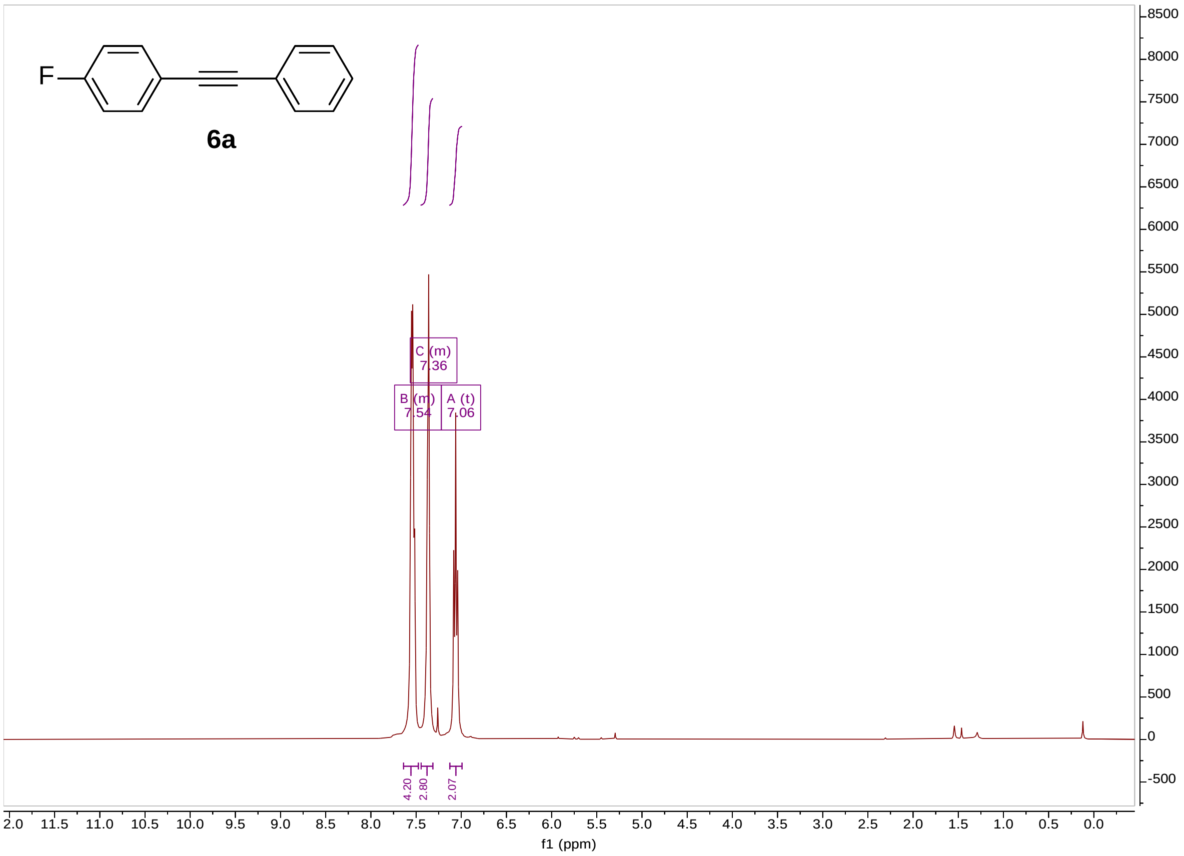

Figure S18. ^{1}H-NMR spectra of compound (**6a**).

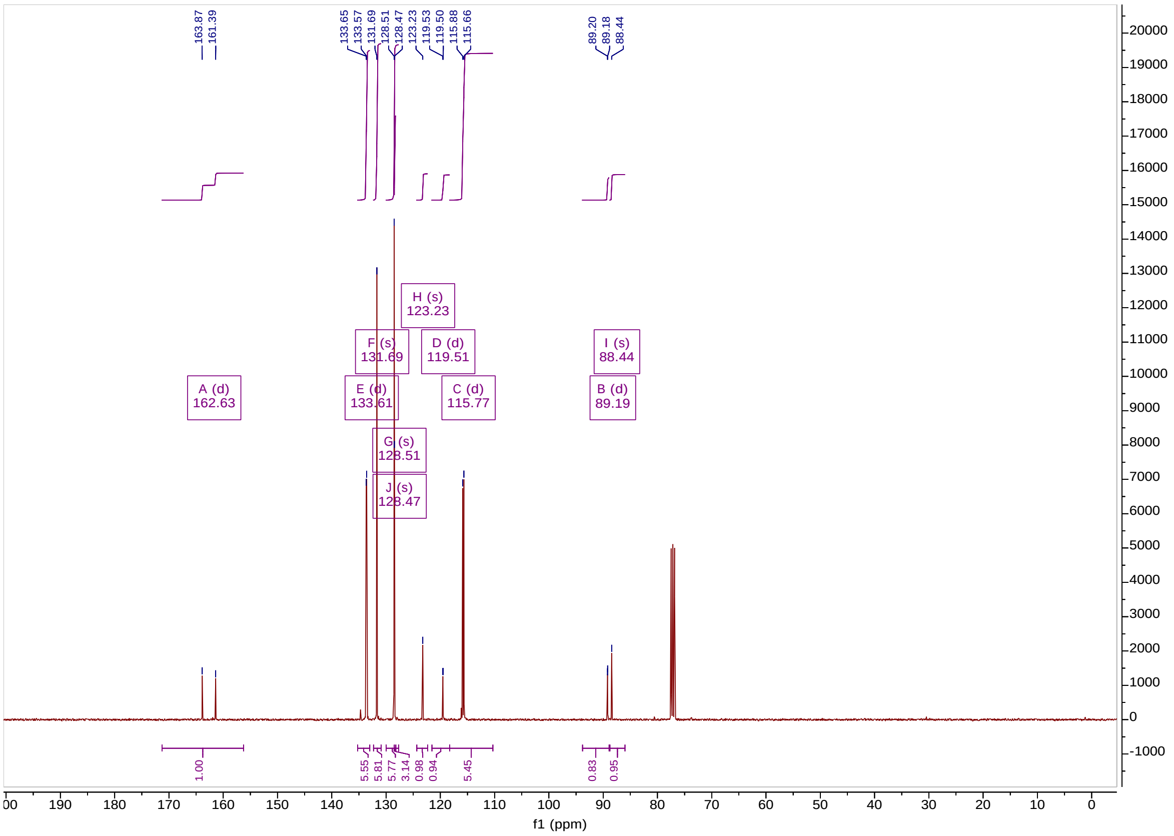

Figure S19. ^{13}C-NMR spectra of compound (**6a**).

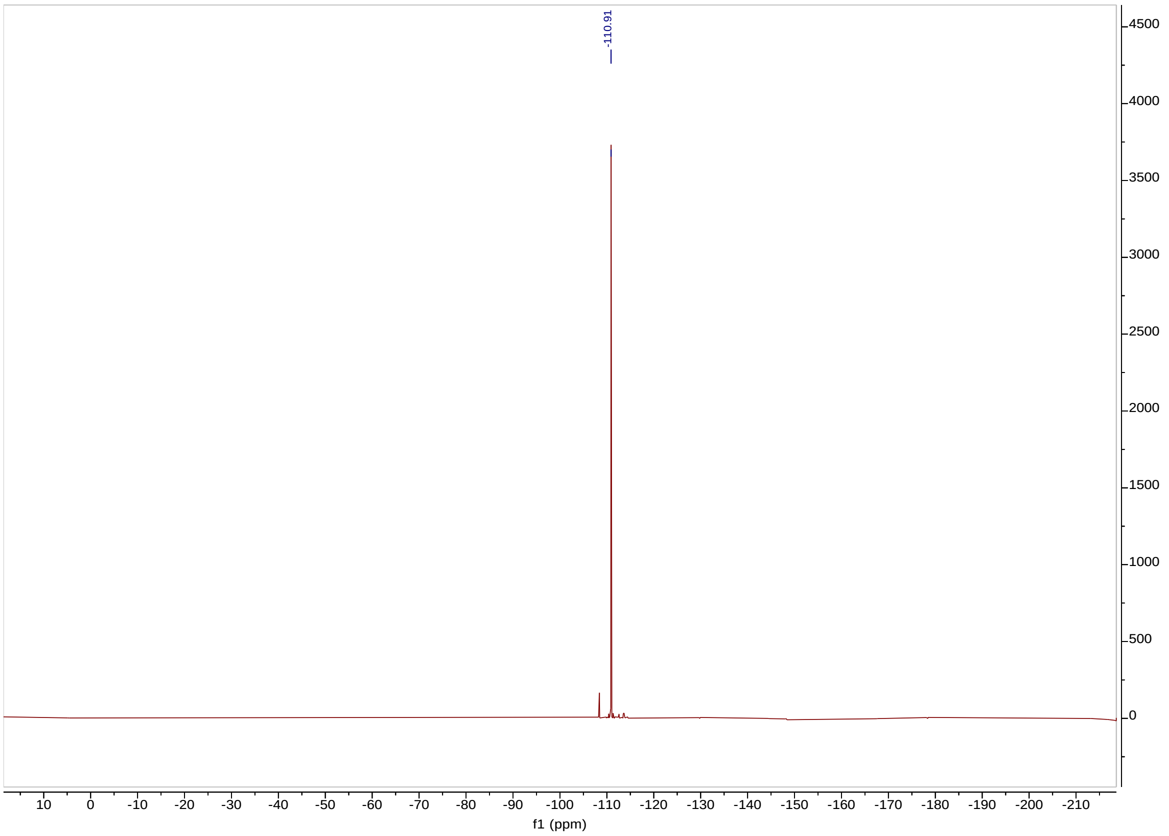

Figure S20. ^{11}F-NMR spectra of compound (**6a**).

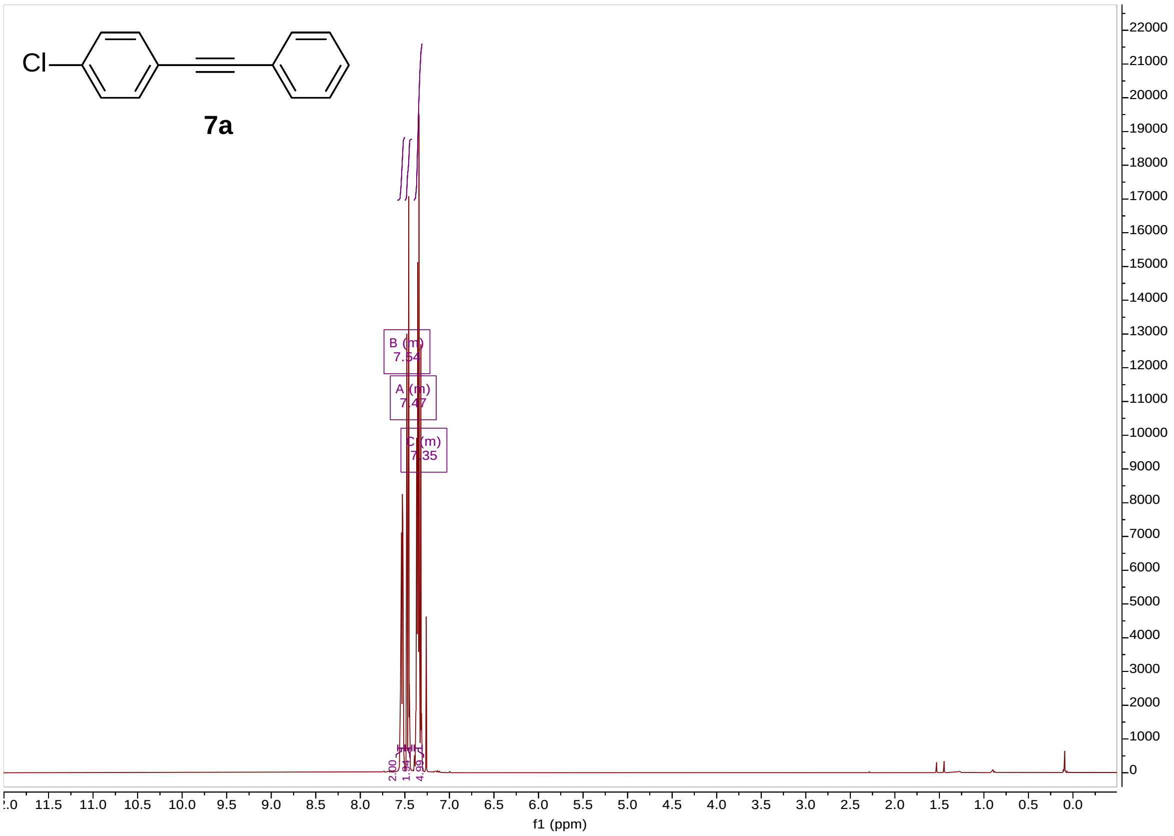

Figure S21. ^{1}H-NMR spectra of compound (**7a**).

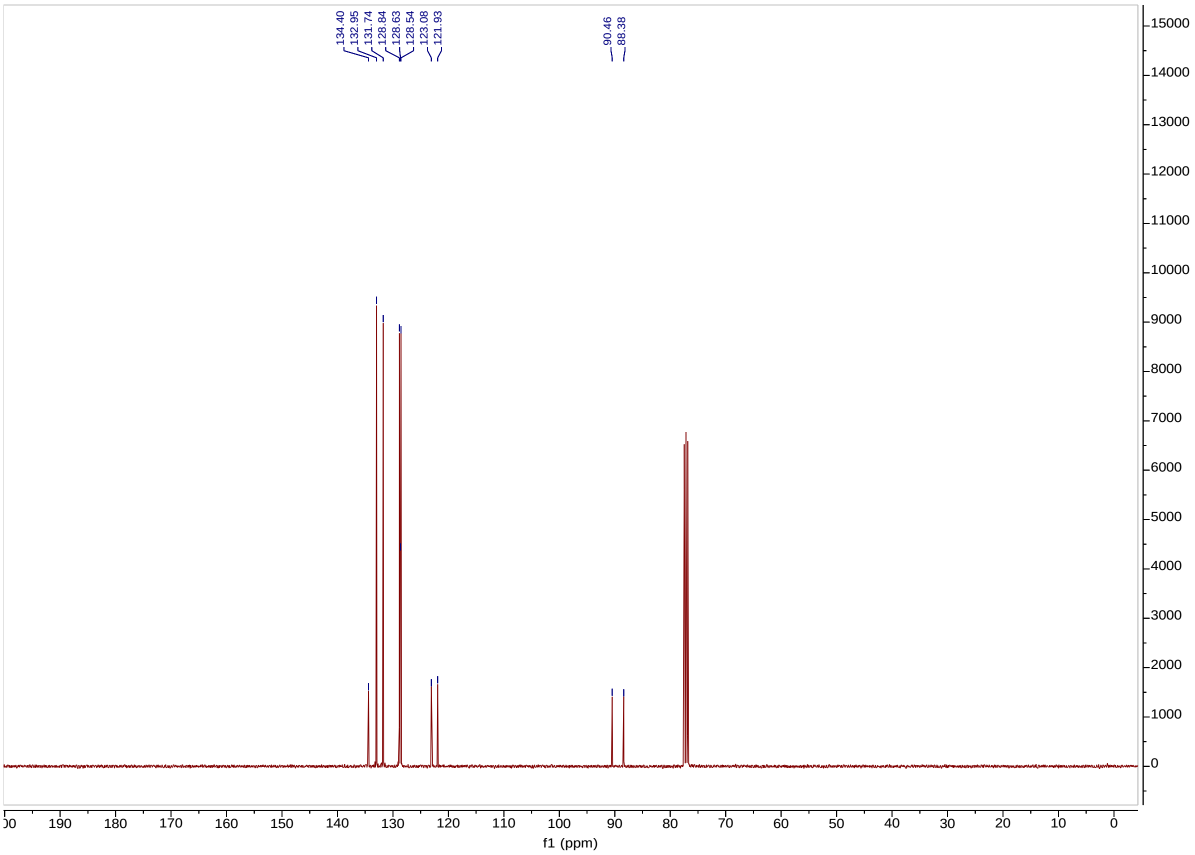

Figure S22. ^{13}C-NMR spectra of compound (**7a**).

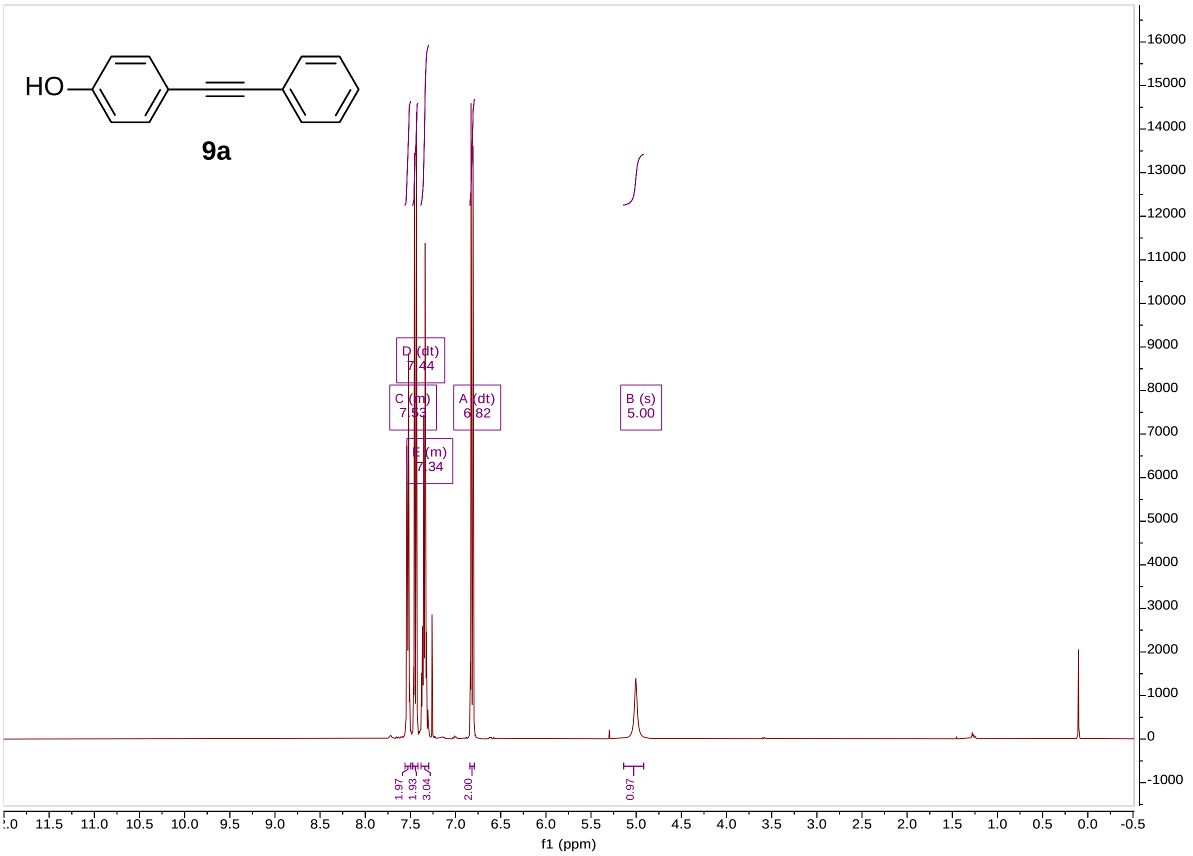

Figure S23. ^{1}H-NMR spectra of compound (**9a**).

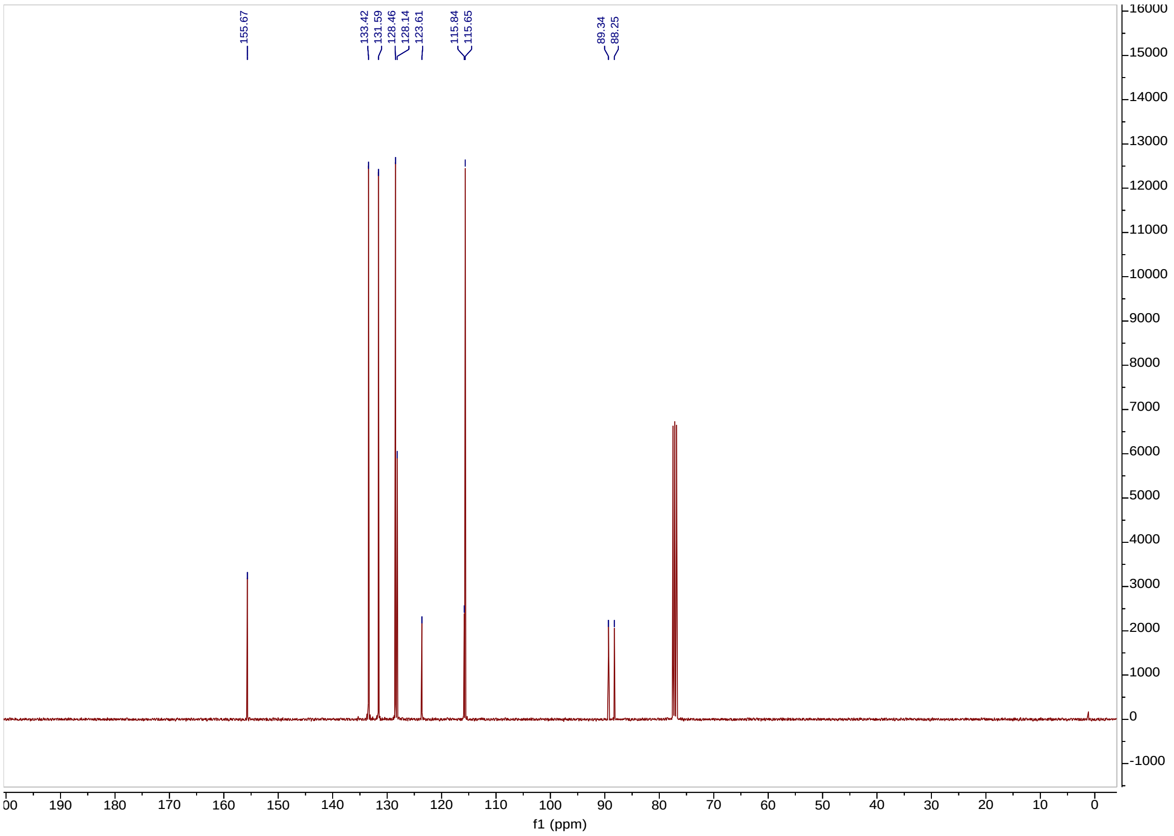

Figure S24. ^{13}C-NMR spectra of compound (**9a**).

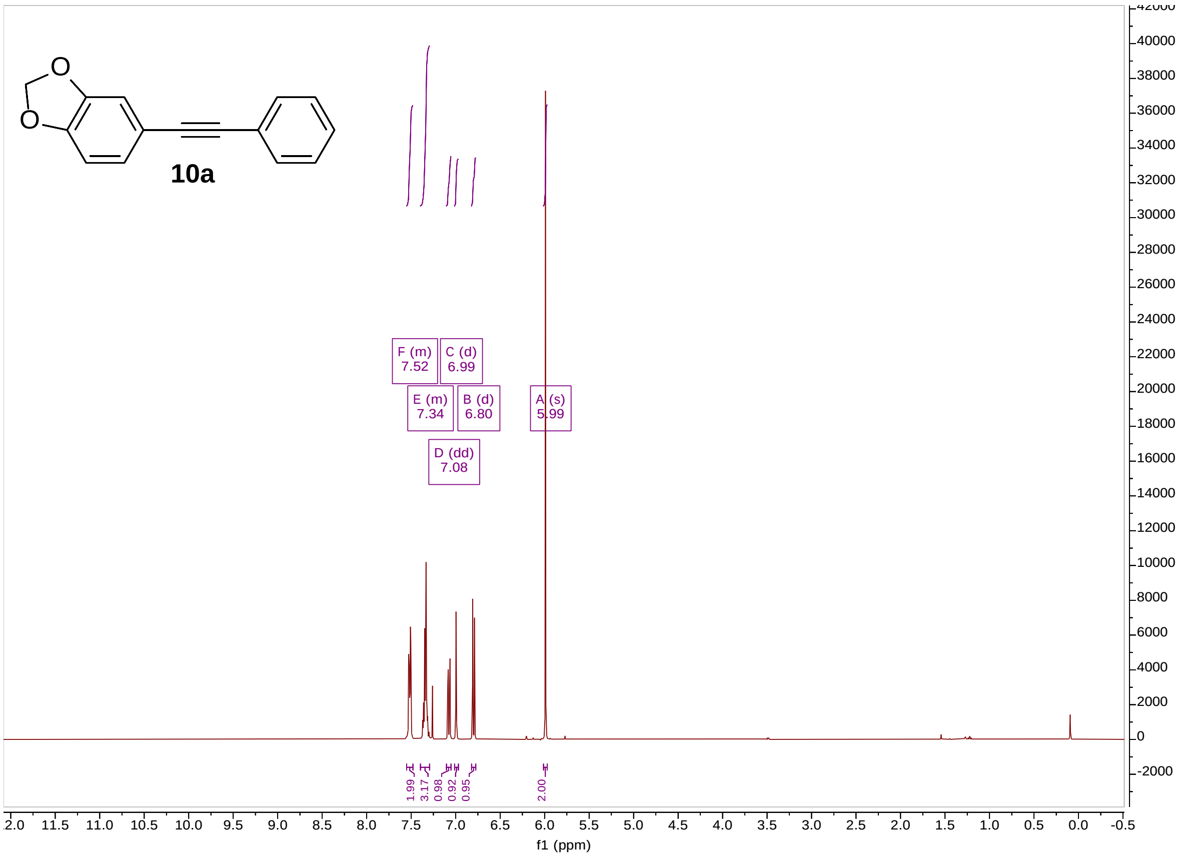

Figure S25. ^{1}H-NMR spectra of compound (**10a**).

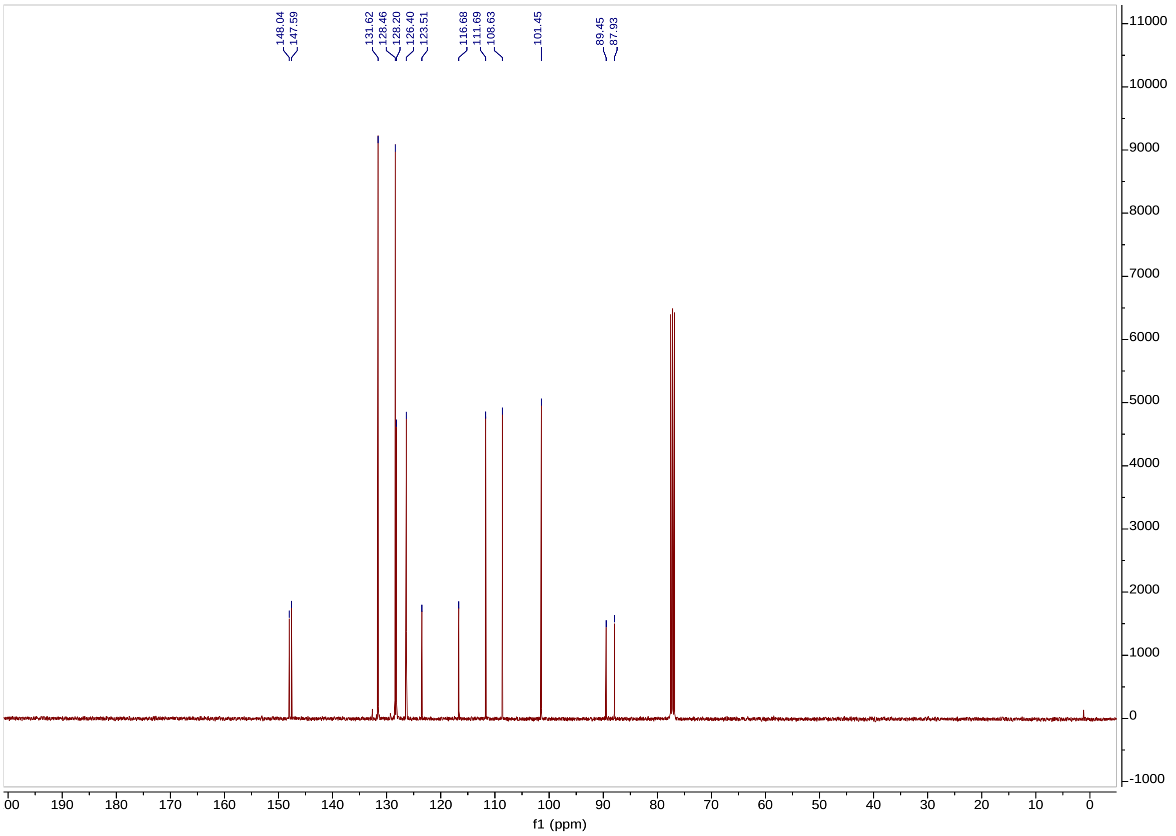

Figure S26. ^{13}C-NMR spectra of compound (**10a**).

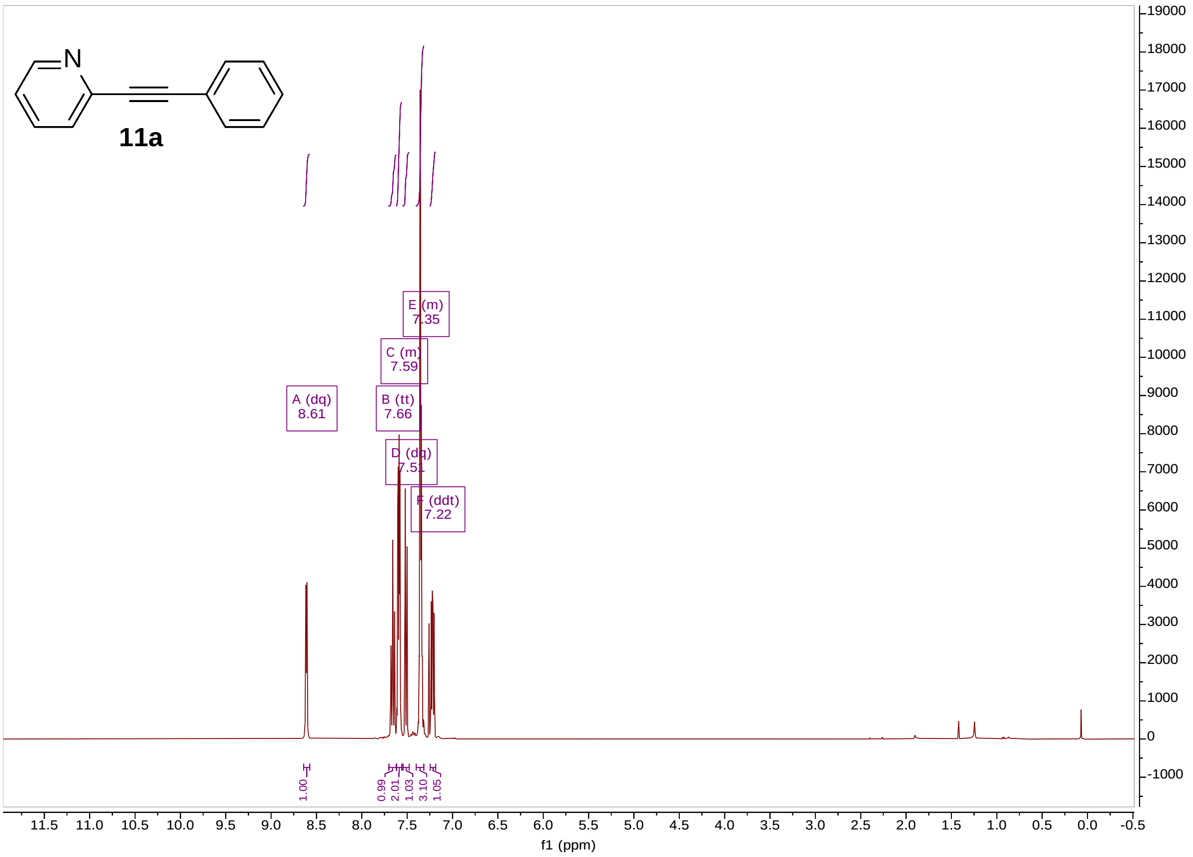

Figure S27. ^{1}H-NMR spectra of compound (**11a**).

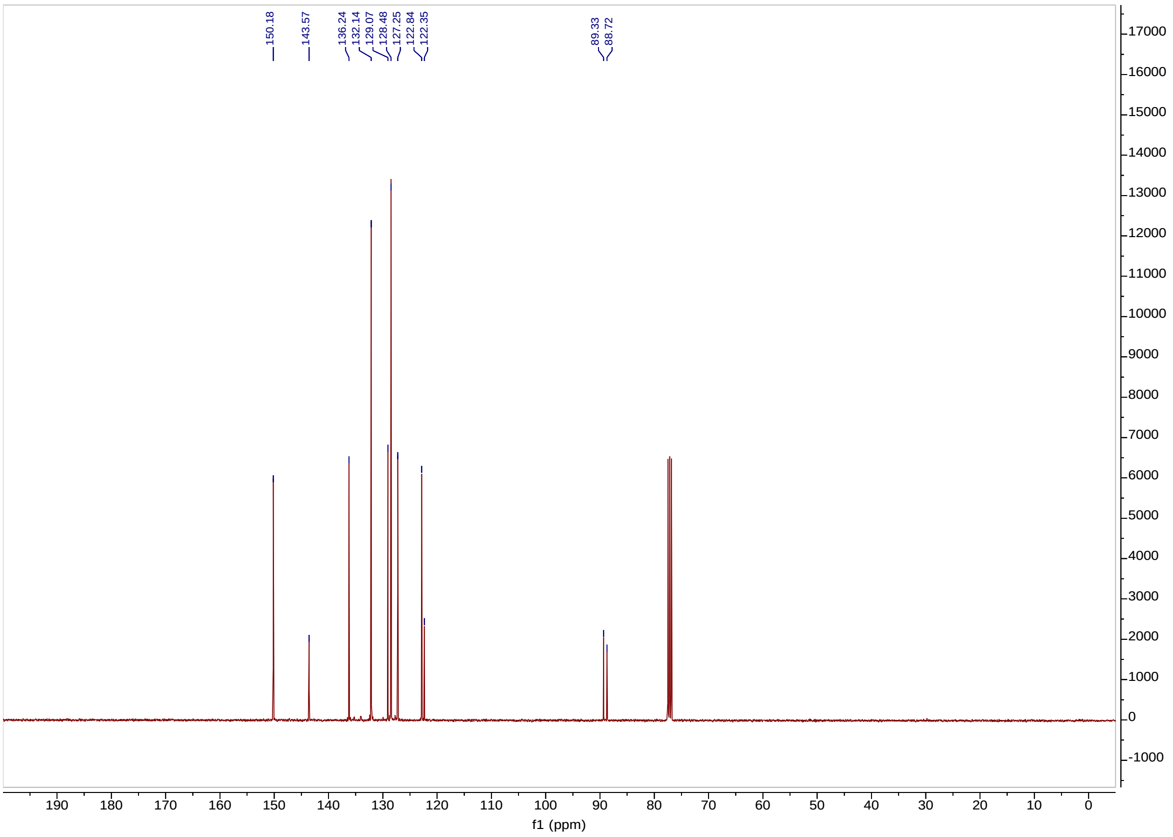

Figure S28. ^{13}C-NMR spectra of compound (**11a**).

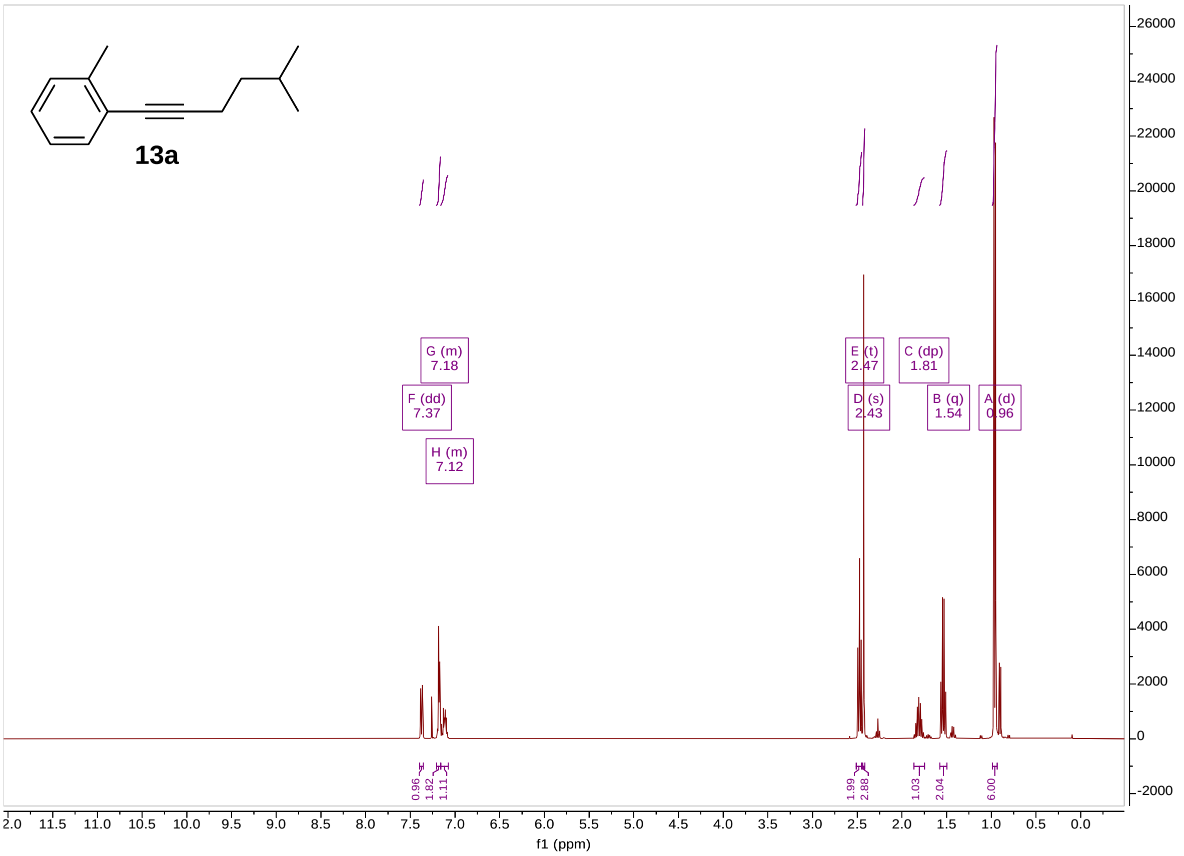

Figure S29. ^{1}H-NMR spectra of compound (**13a**).

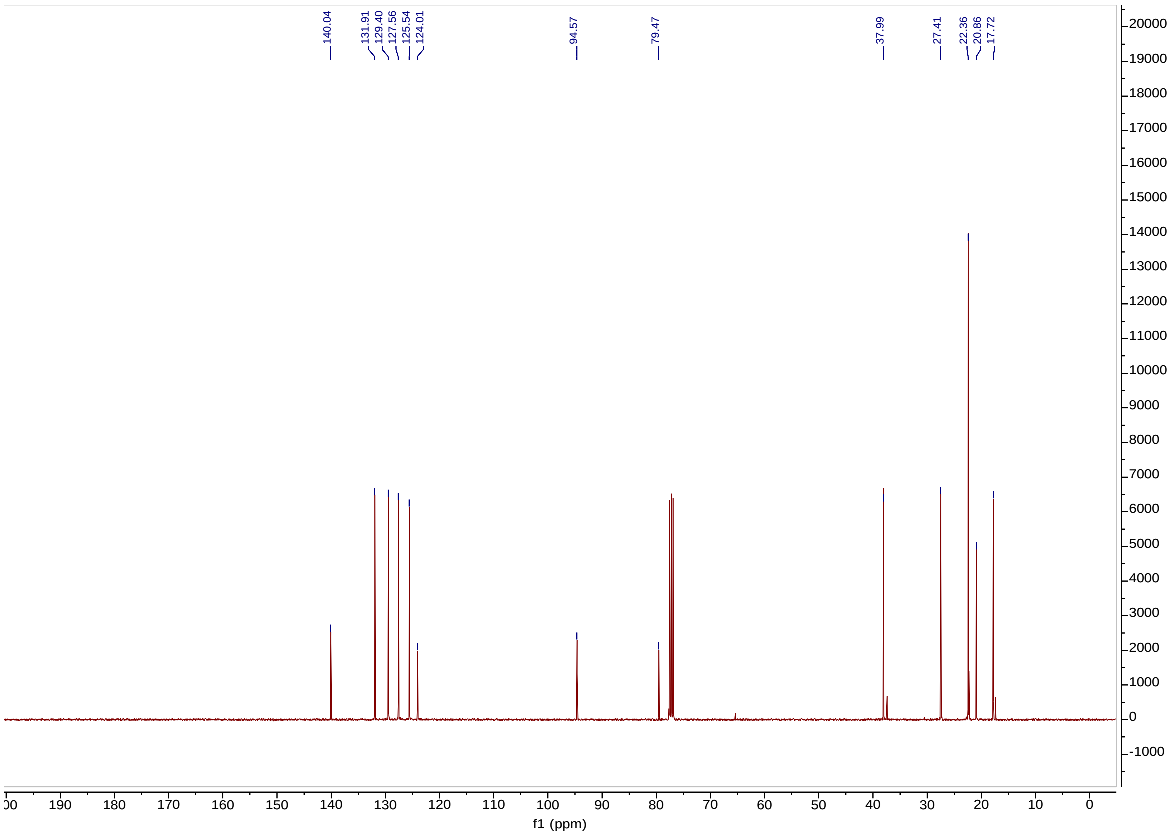

Figure S30. ^{13}C-NMR spectra of compound (**13a**).

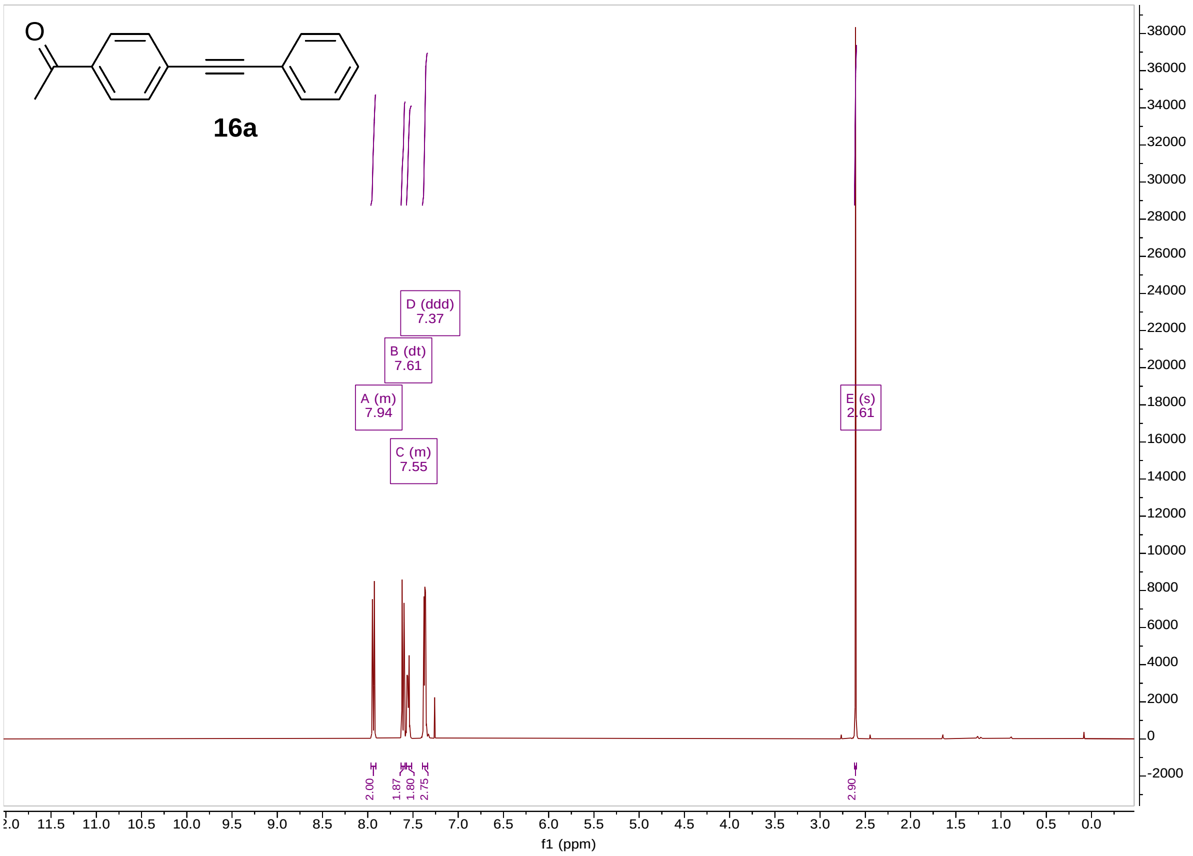

Figure S31. ^{1}H-NMR spectra of compound (**16a**).

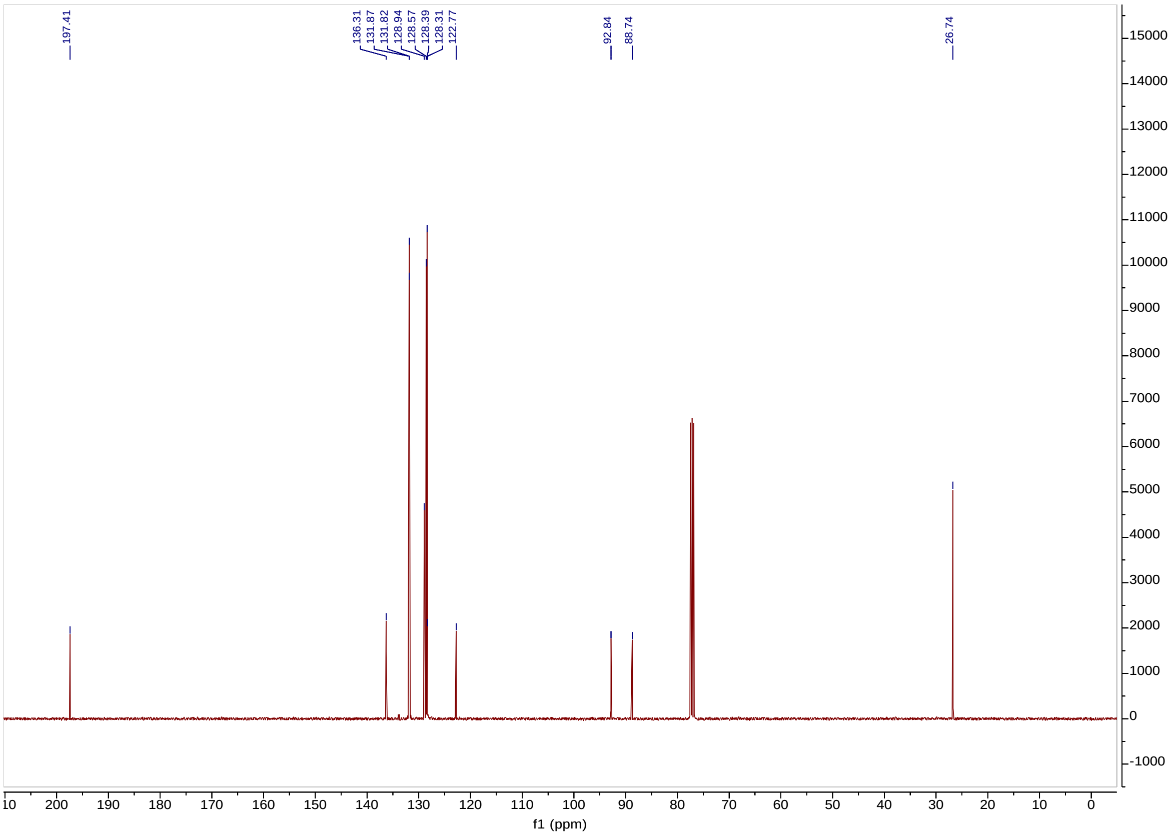

Figure S32. ^{13}C-NMR spectra of compound (**16a**).

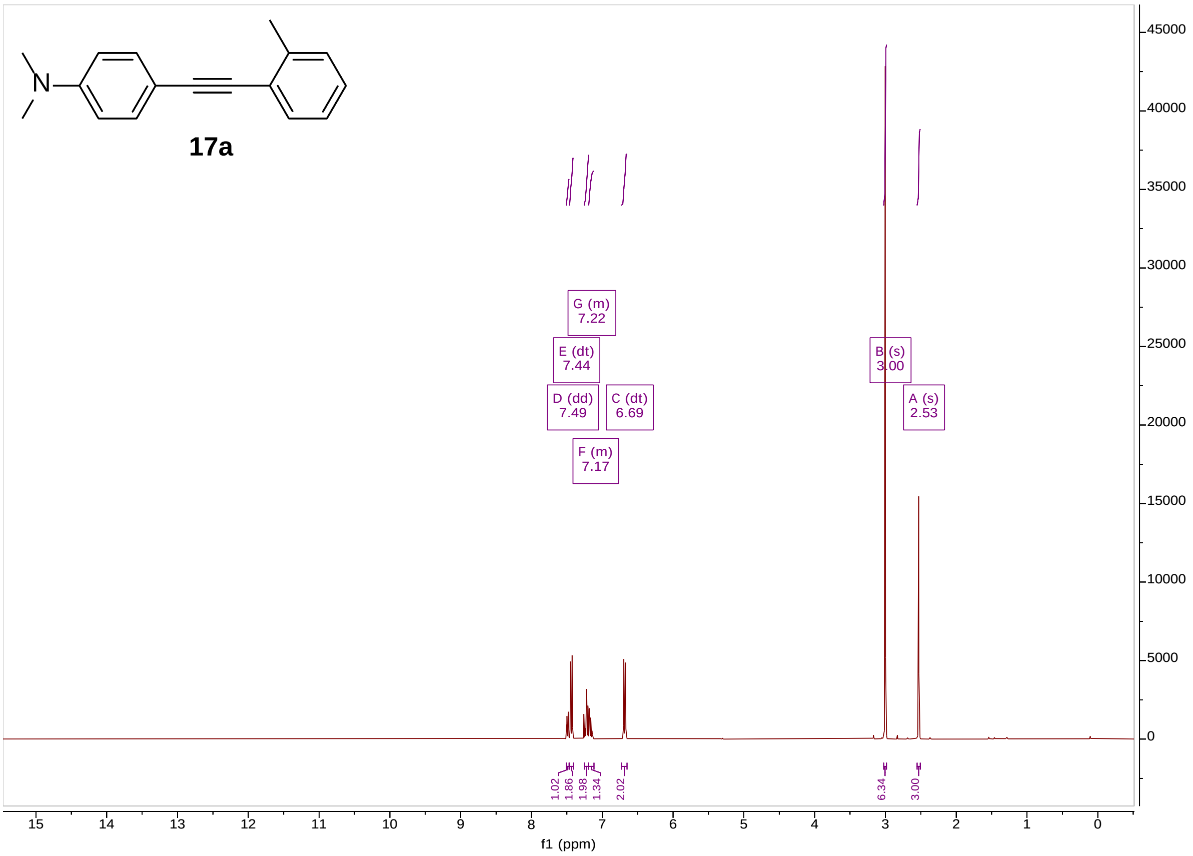

Figure S33. ^{1}H-NMR spectra of compound (**17a**).

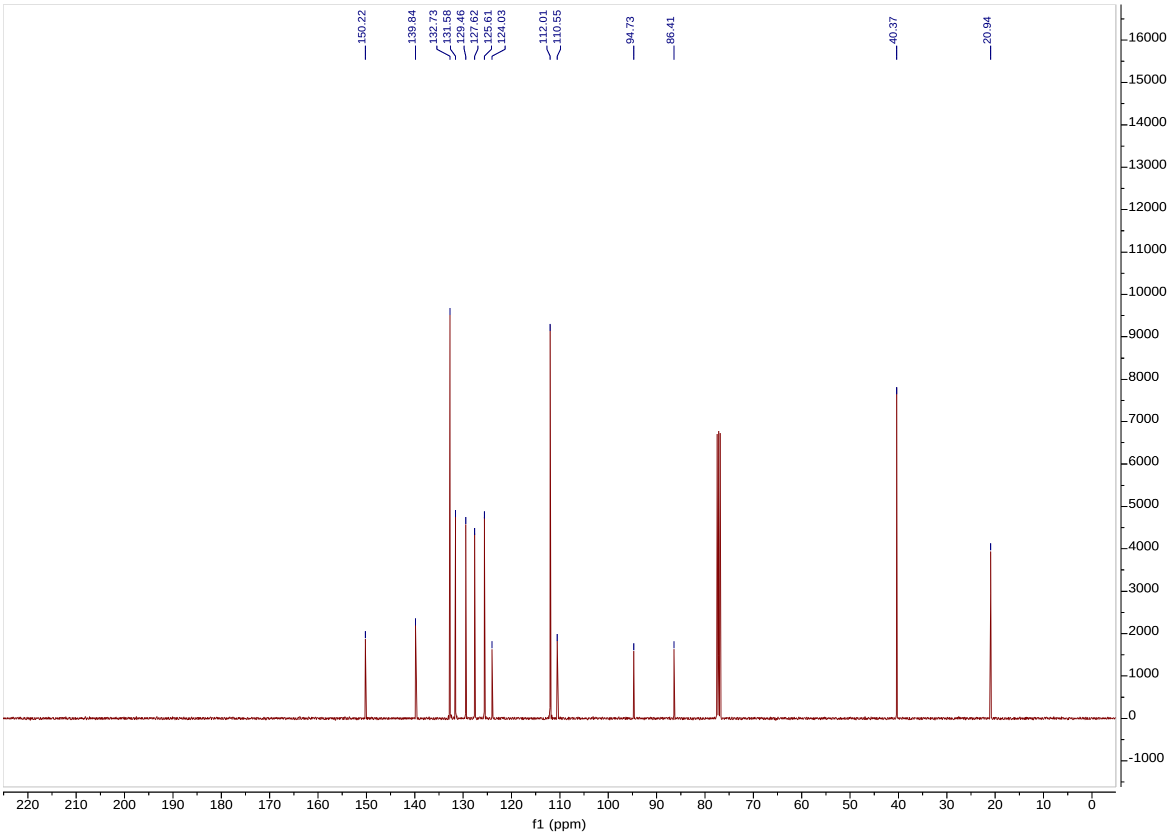

Figure S34. ^{13}C-NMR spectra of compound (**17a**).

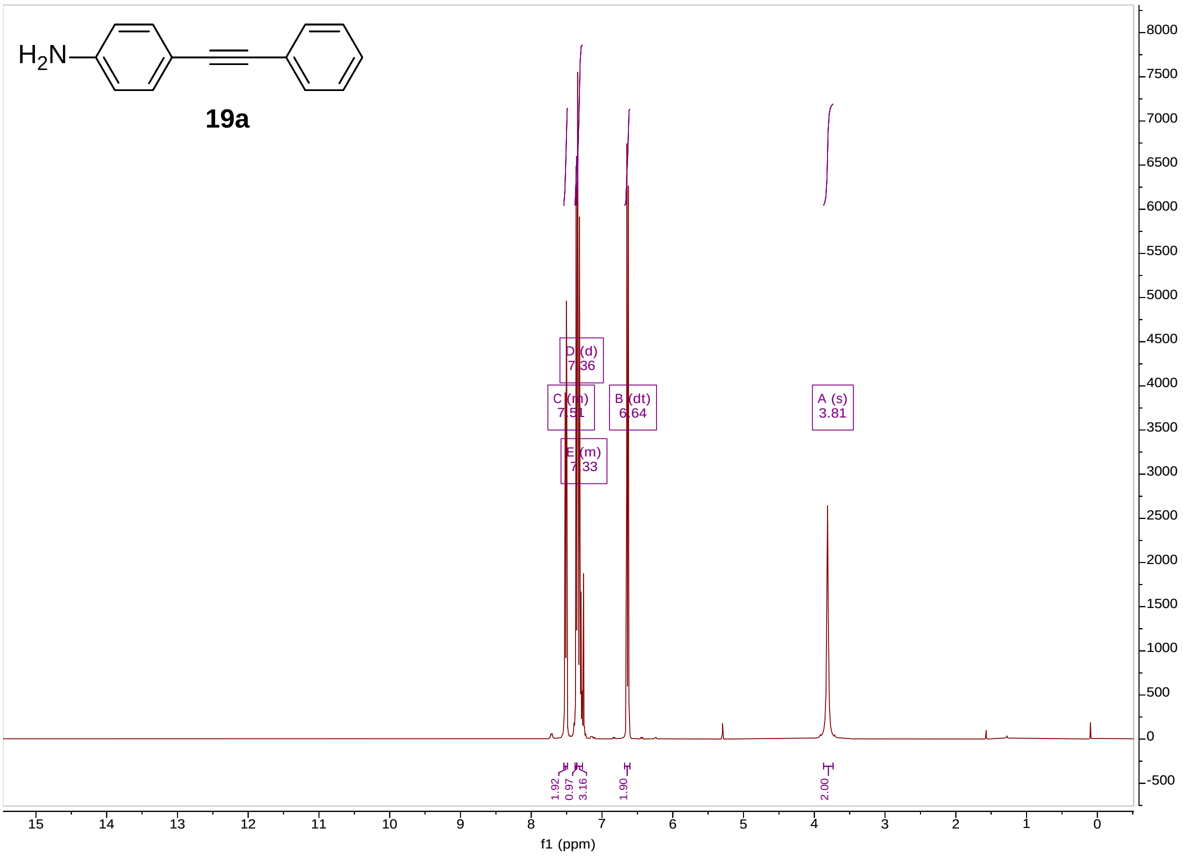

Figure S35. ^{1}H-NMR spectra of compound (**19a**).

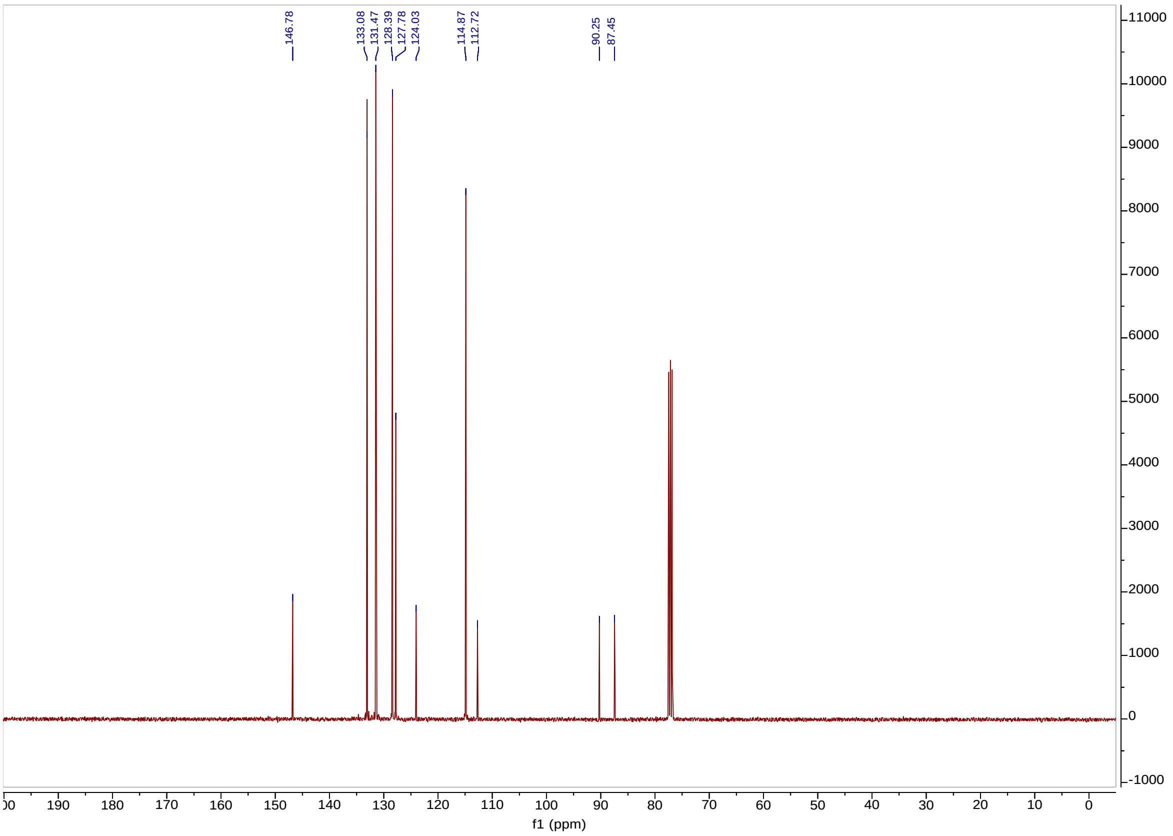

Figure S36. ^{13}C-NMR spectra of compound (**14a**).

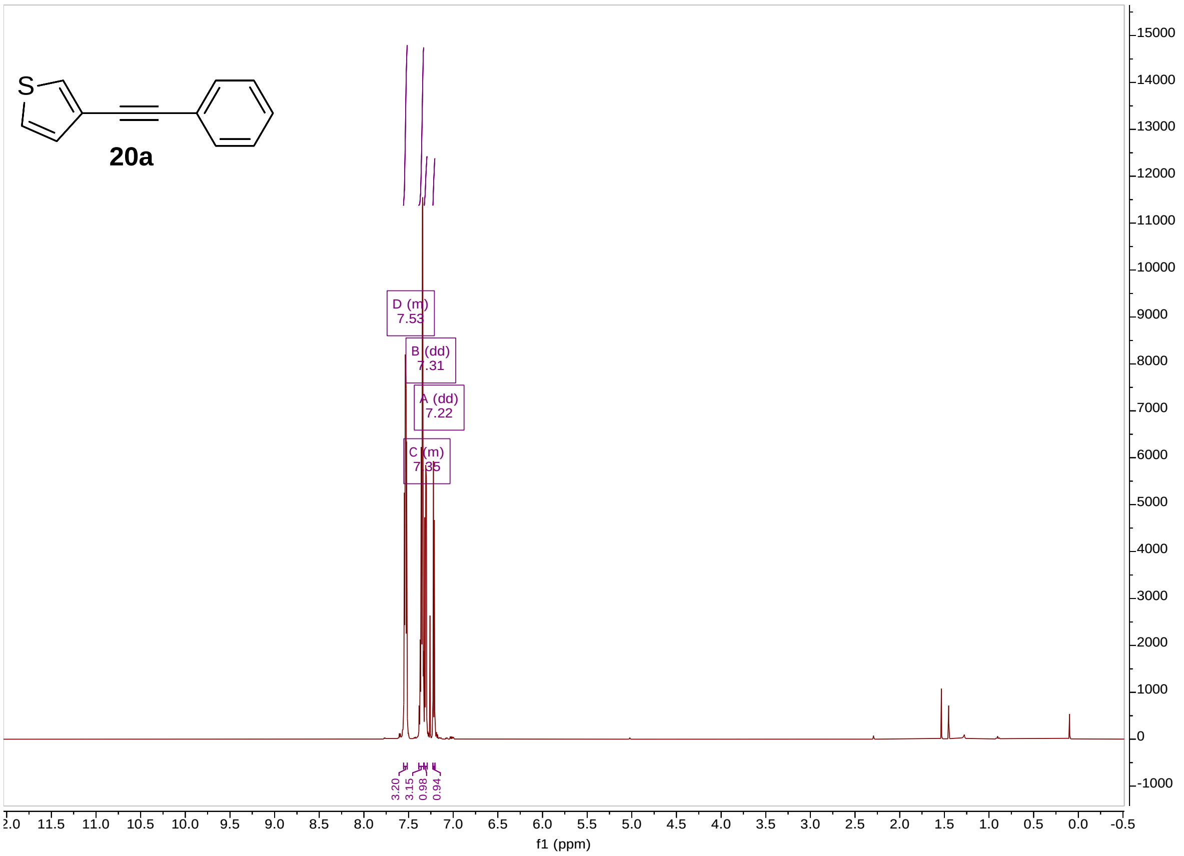

Figure S37. ^{1}H-NMR spectra of compound (**20a**).

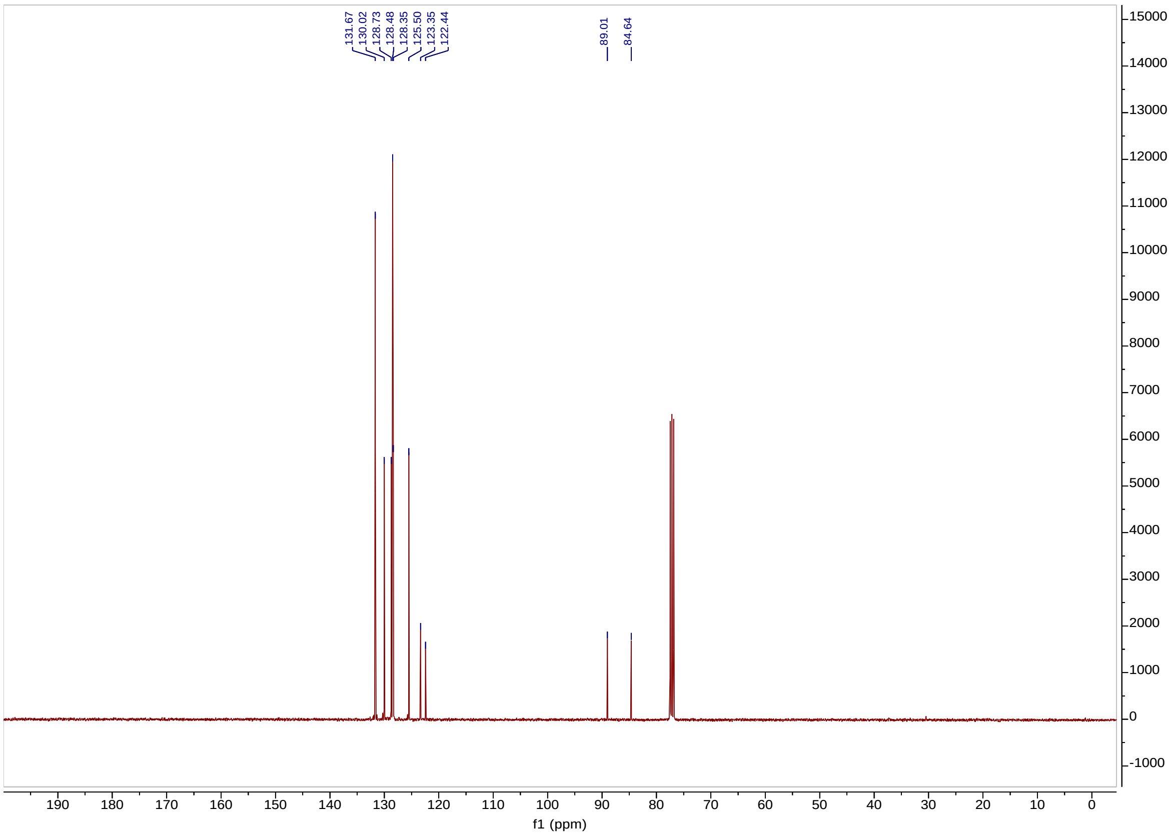

Figure S38. ^{13}C-NMR spectra of compound (**20a**).

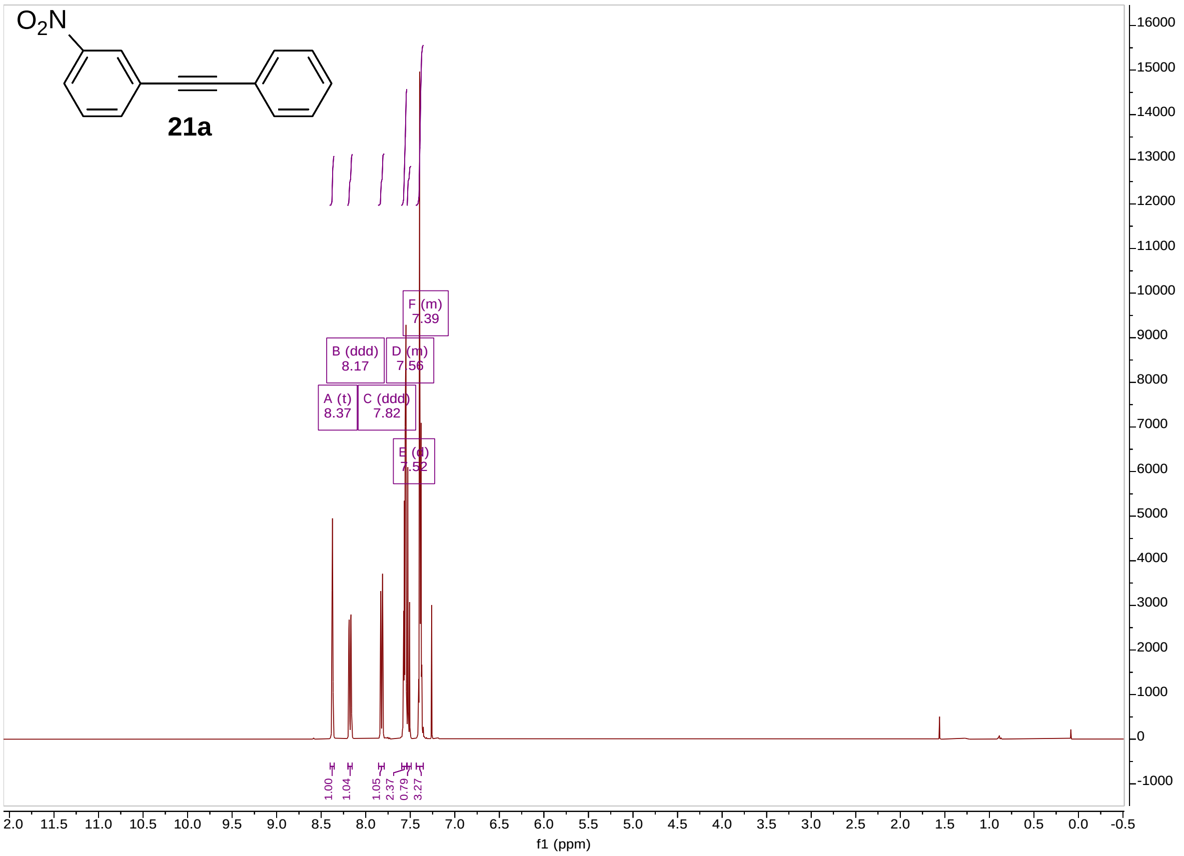

Figure S39. ^{1}H-NMR spectra of compound (**21a**).

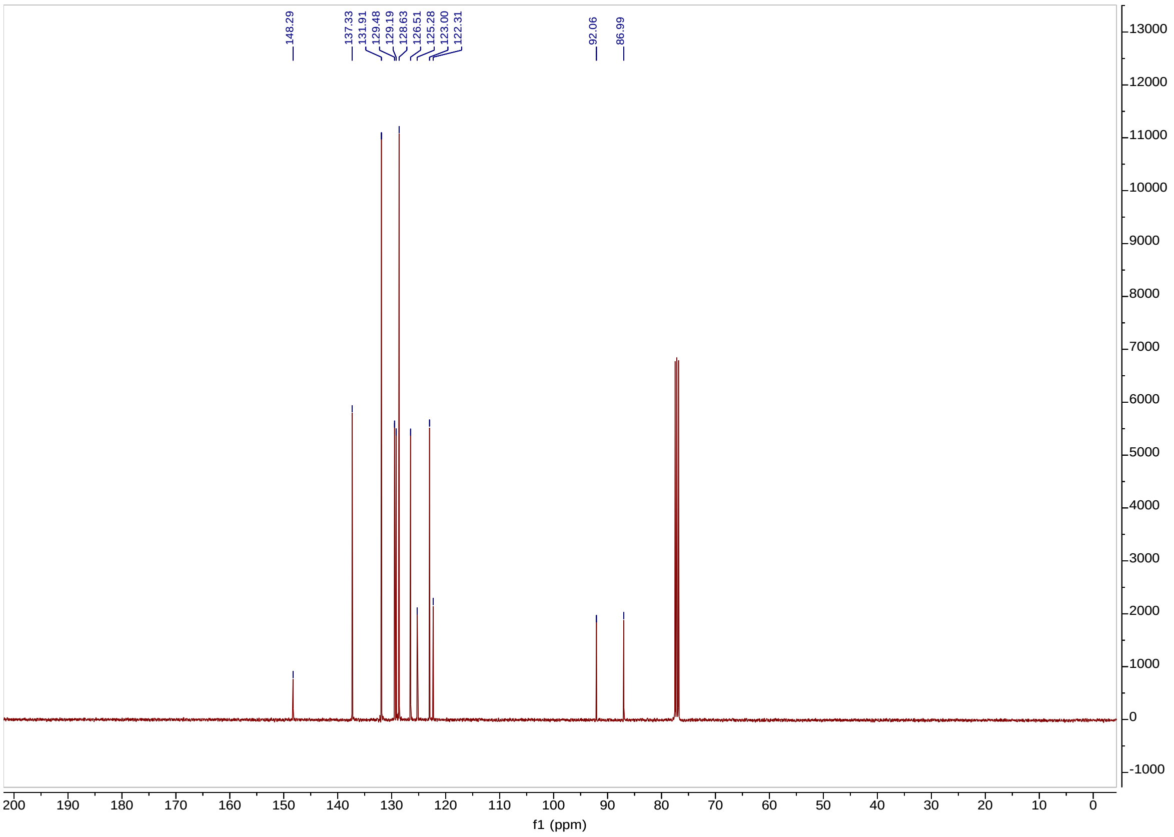

Figure S40. ^{13}C-NMR spectra of compound (**21a**).

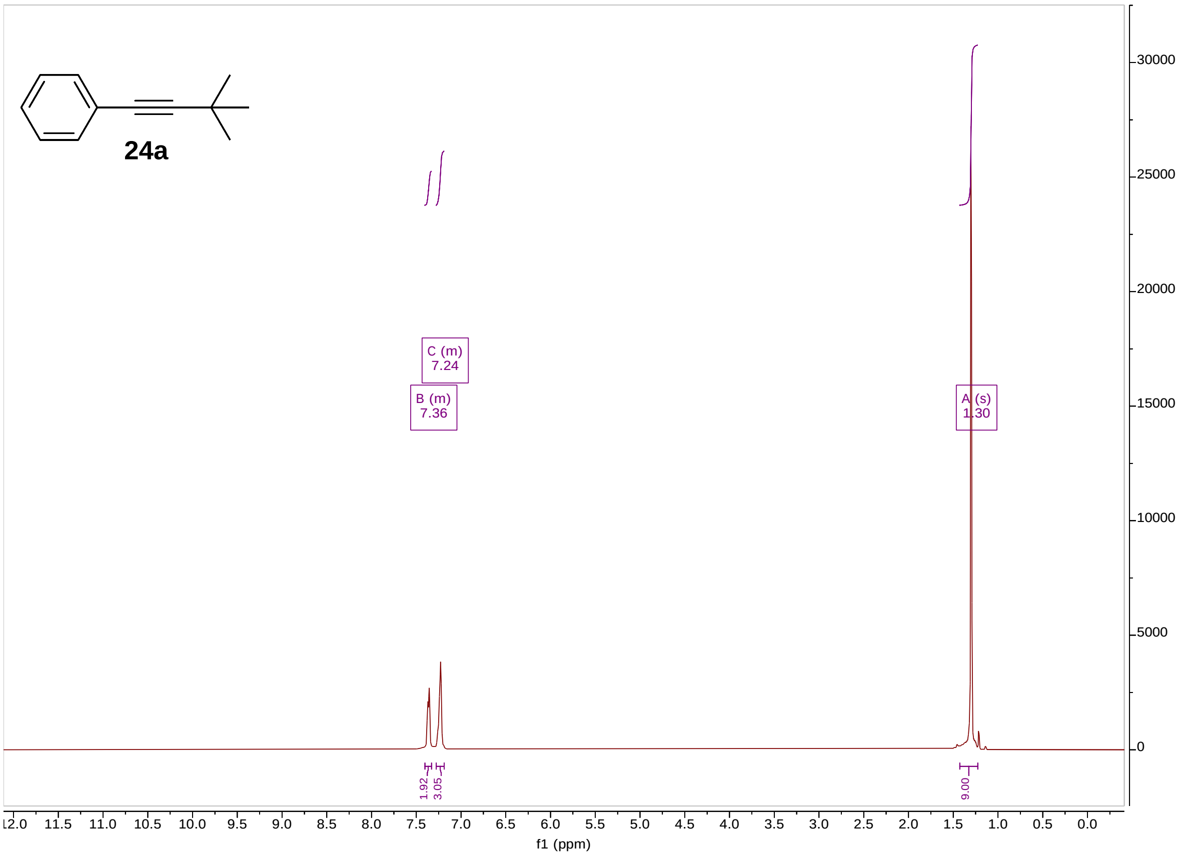

Figure S41. ^{1}H-NMR spectra of compound (**24a**).

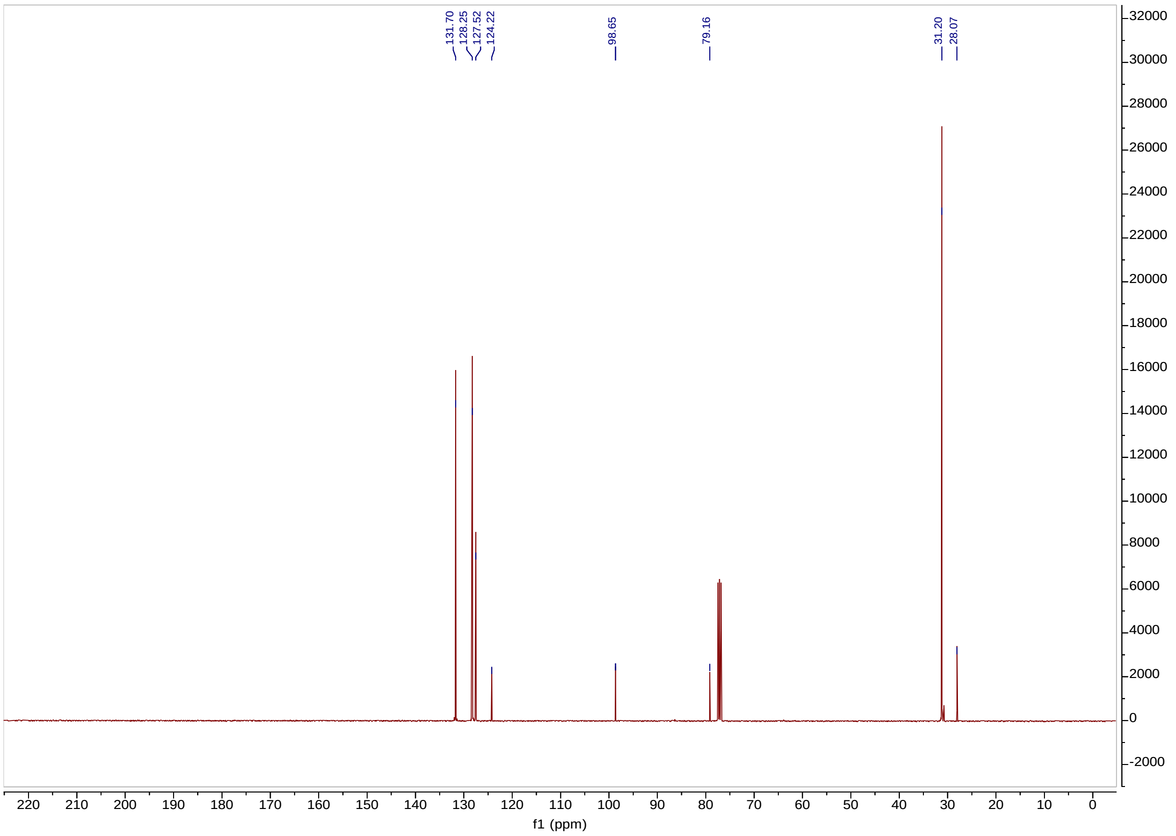

Figure S42. ^{13}C-NMR spectra of compound (**24a**).

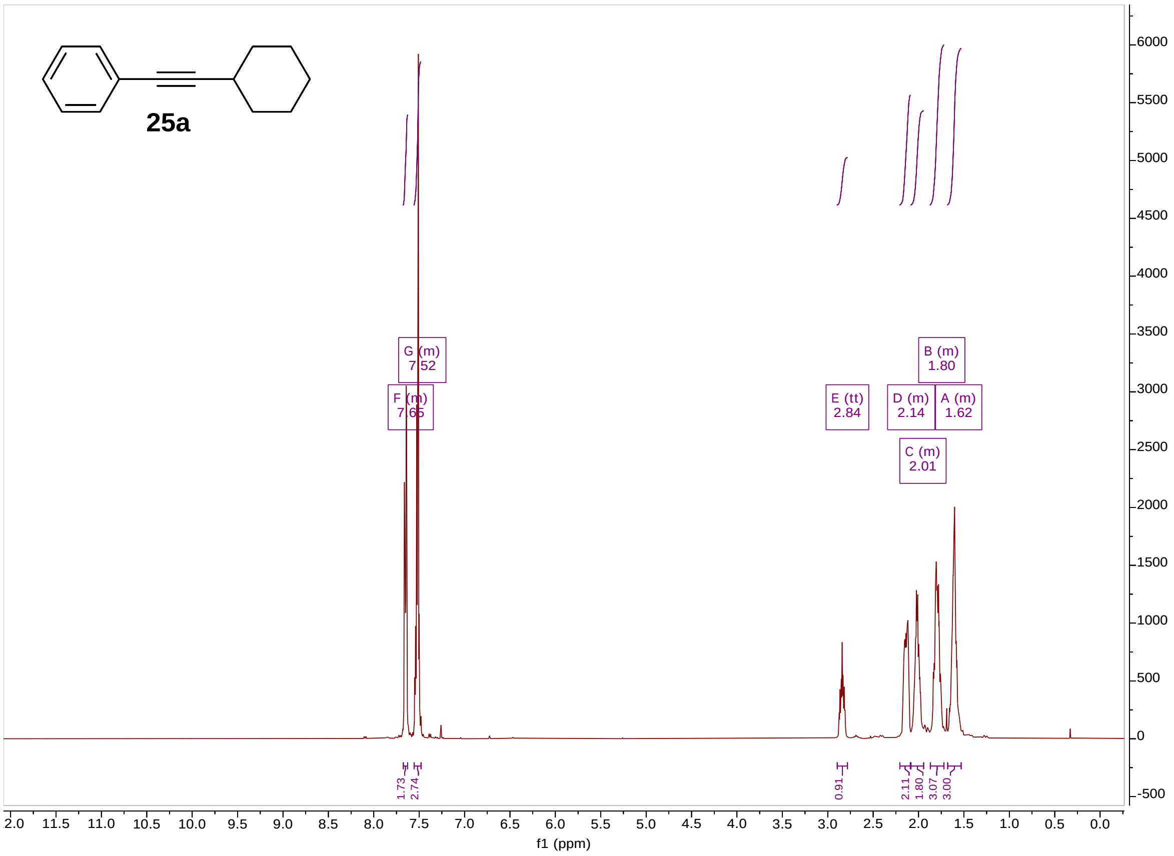

Figure S43. ^{1}H-NMR spectra of compound (**25a**).

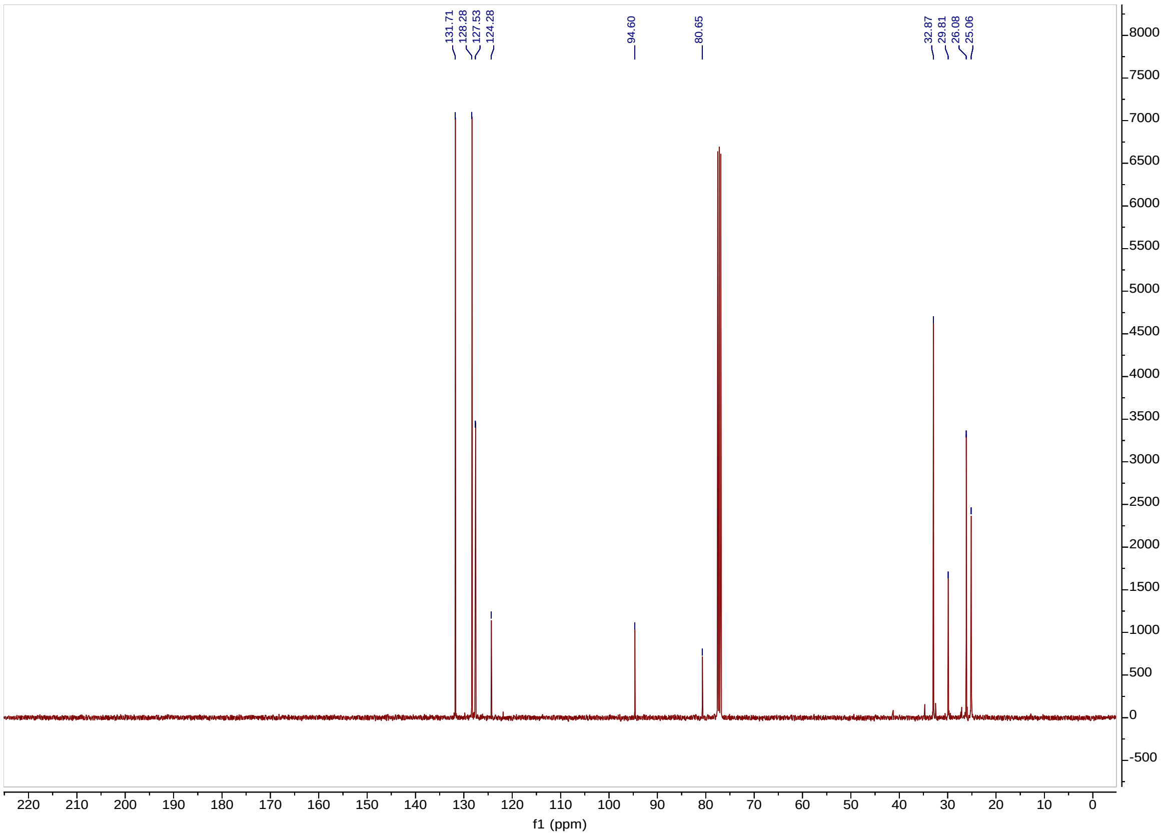

Figure S44. ^{13}C-NMR spectra of compound (**25a**).

Alkenes Spectra:

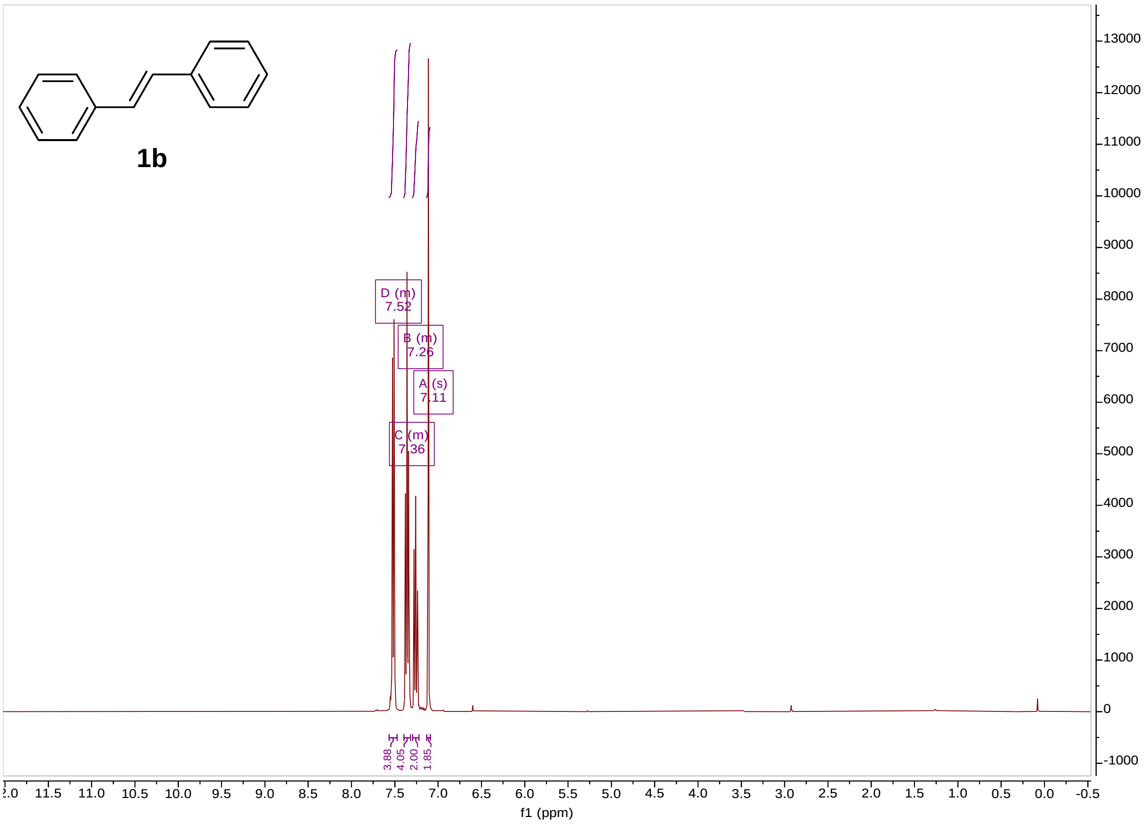

Figure S45. ^{1}H-NMR spectra of compound (**1b**).

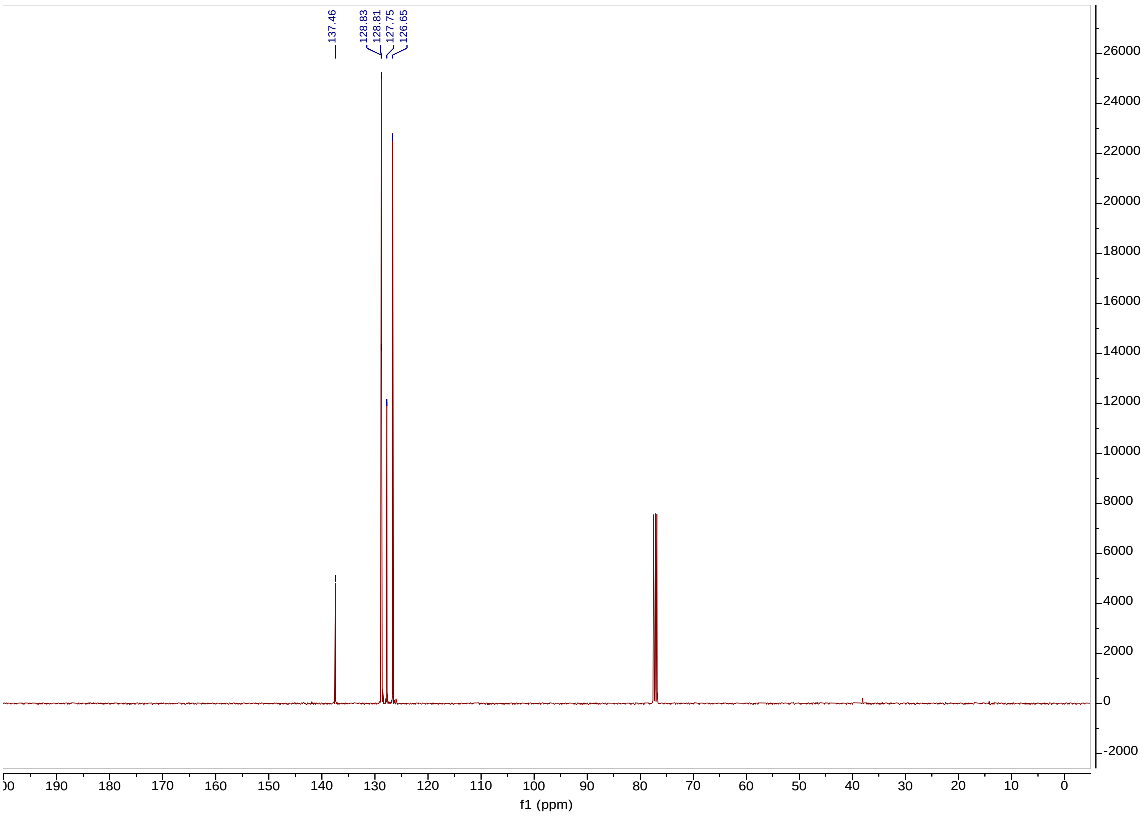

Figure S46. ^{13}C-NMR spectra of compound (**1b**).

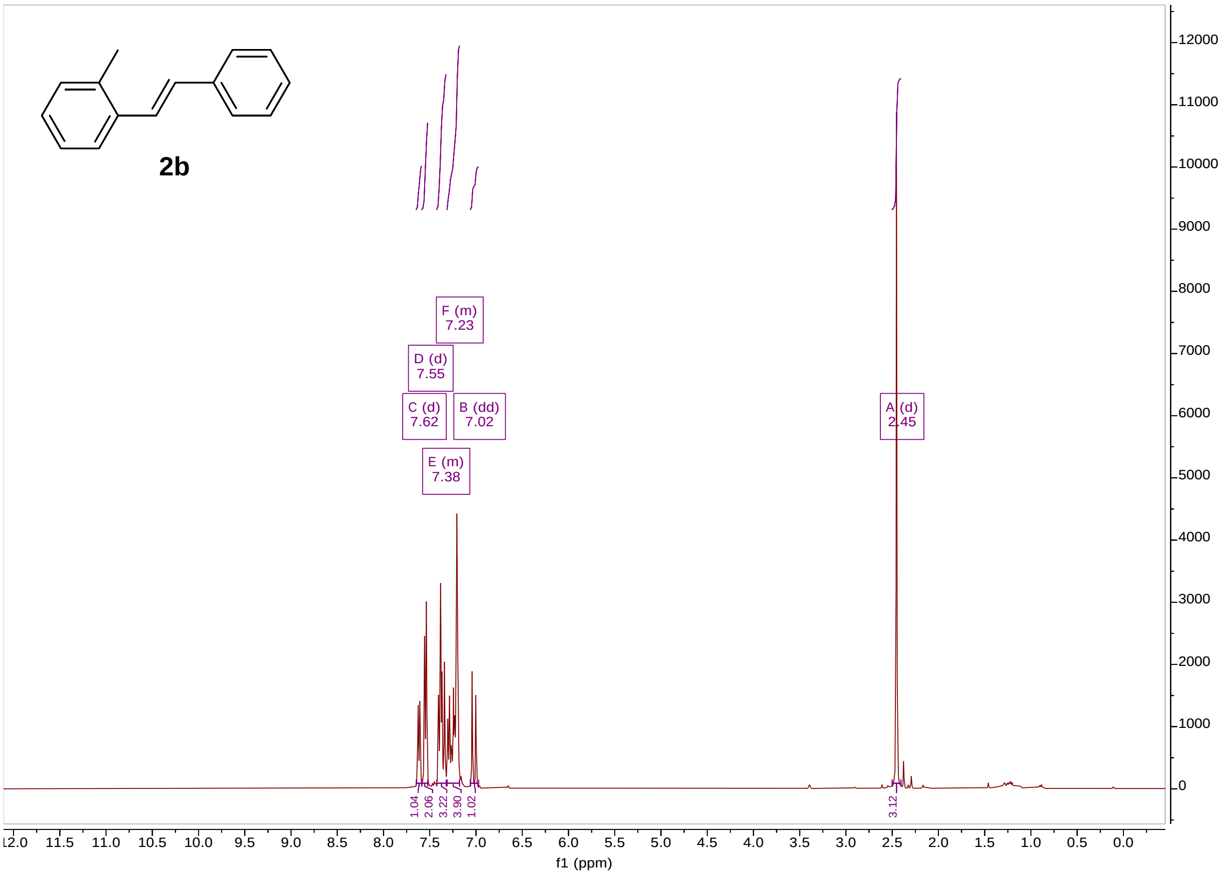

Figure S47. ^{1}H-NMR spectra of compound (**2b**).

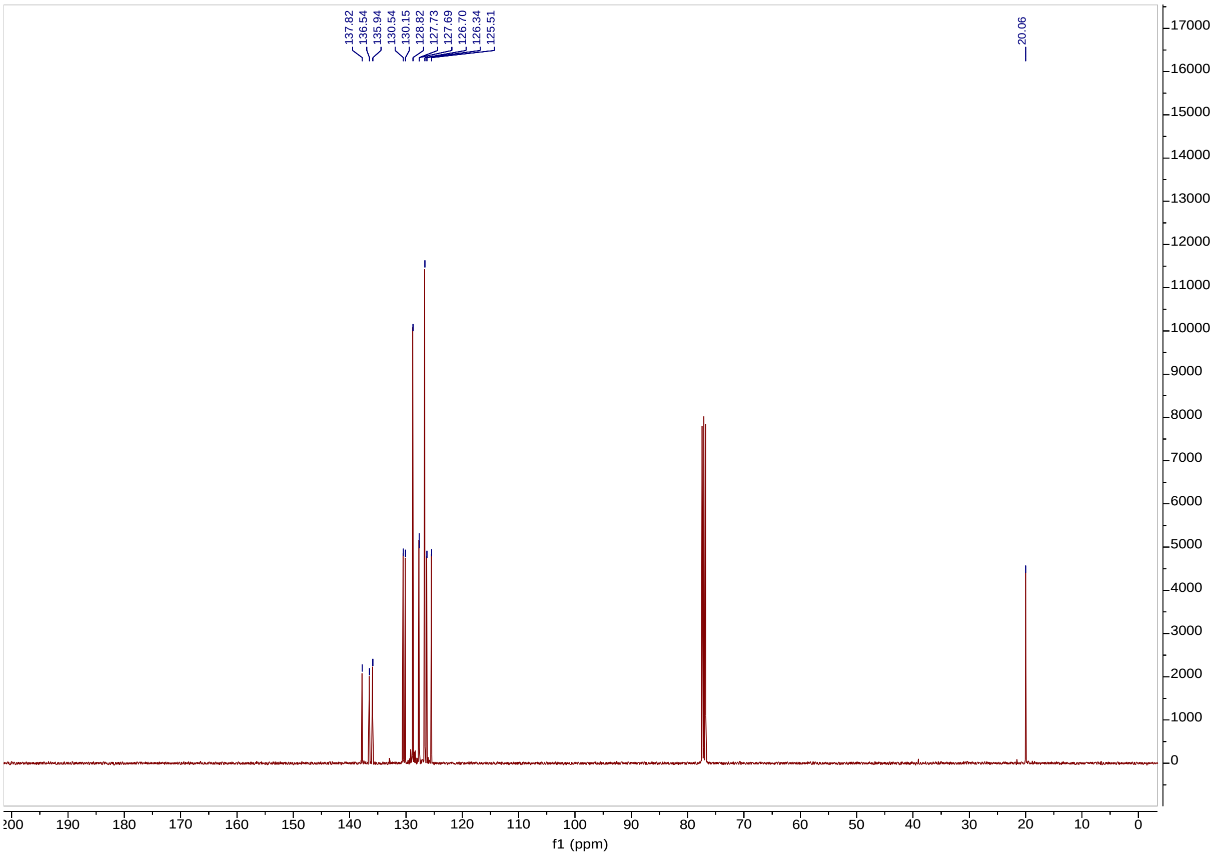

Figure S48. ^{13}C-NMR spectra of compound (**2b**).

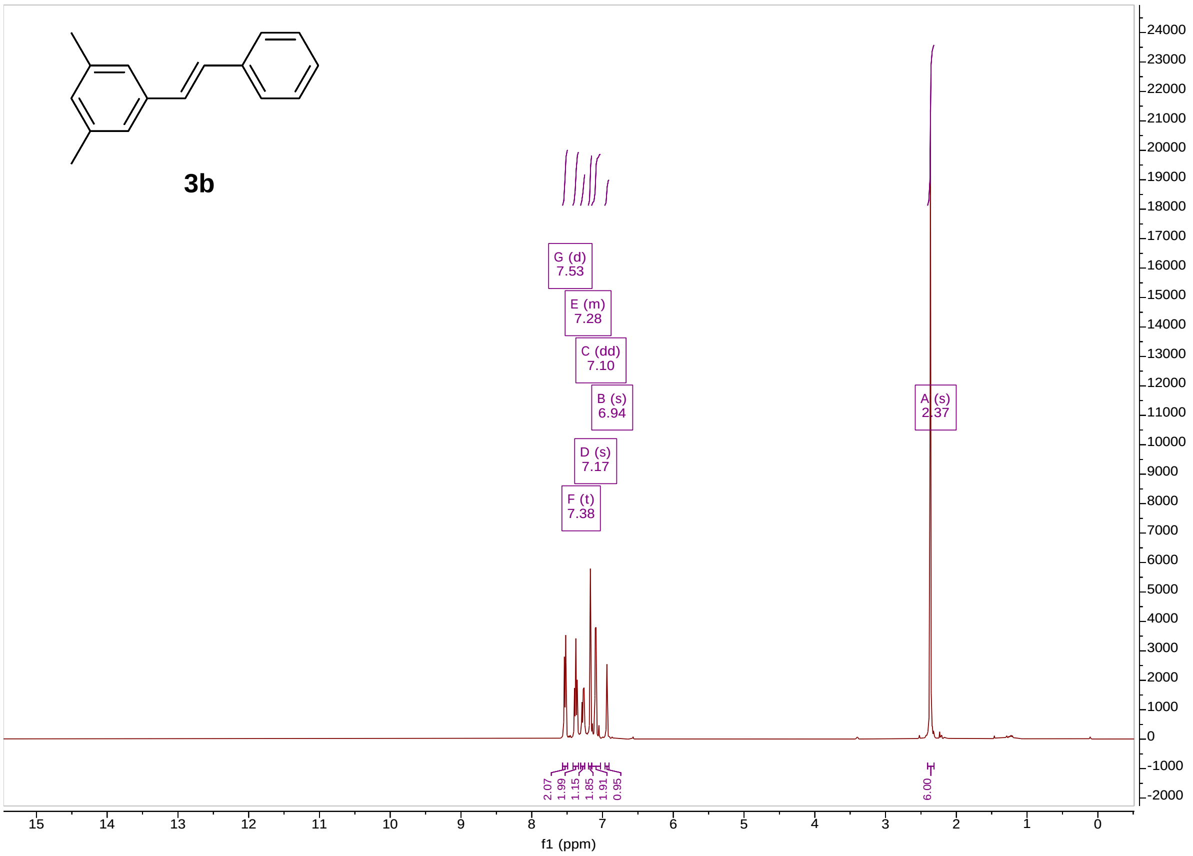

Figure S49. ^{1}H-NMR spectra of compound (**3b**).

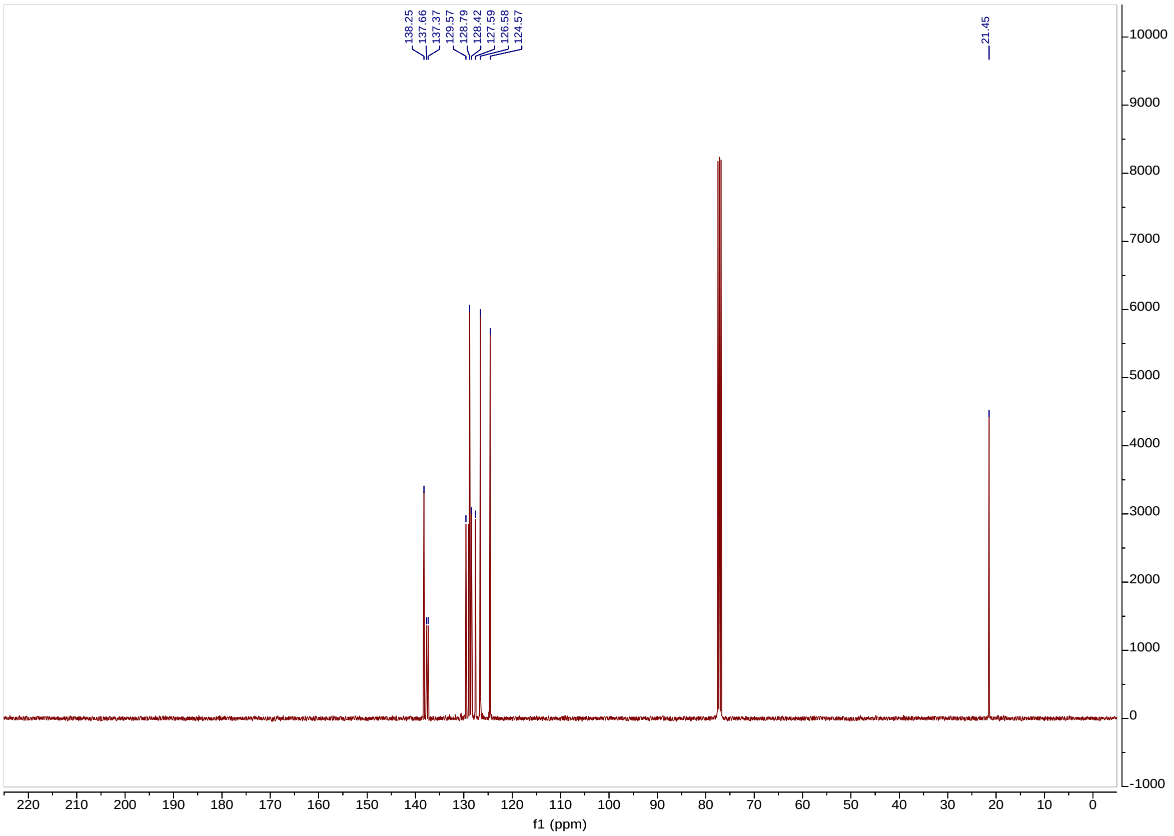

Figure S50. ^{13}C-NMR spectra of compound (**3b**).

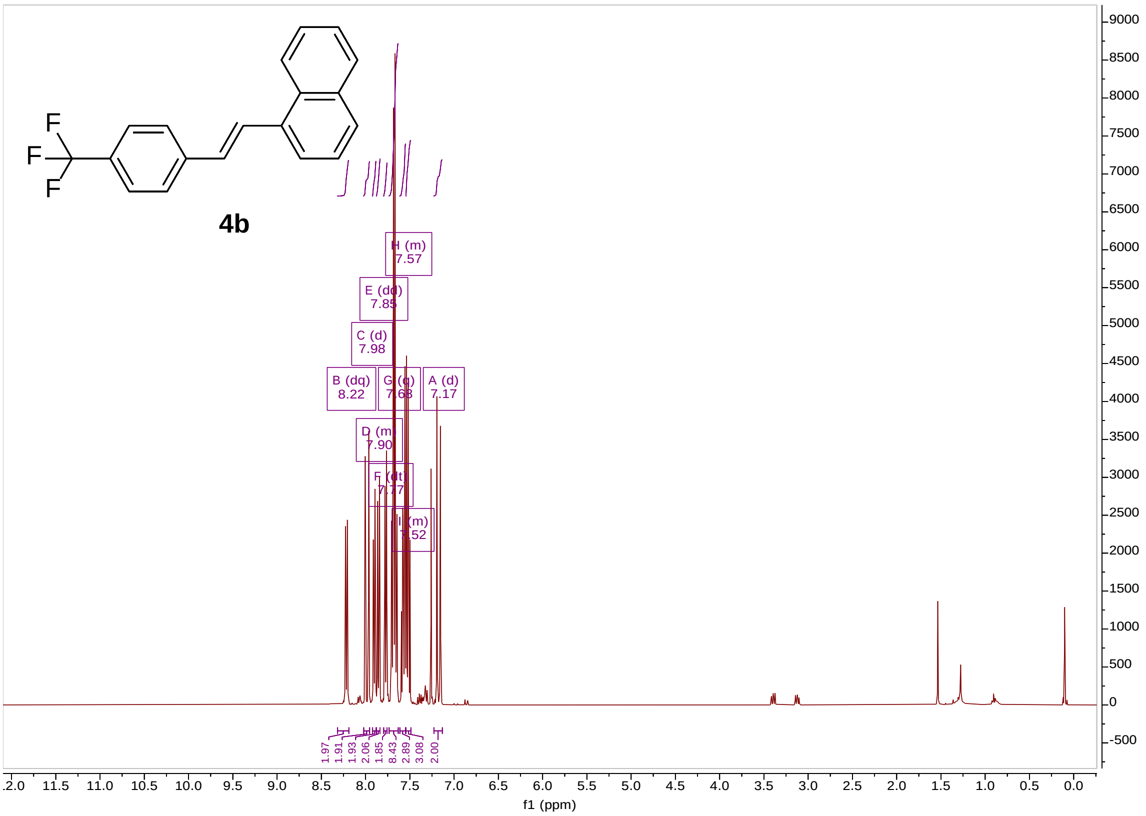

Figure S51. ^{1}H-NMR spectra of compound (**4b**).

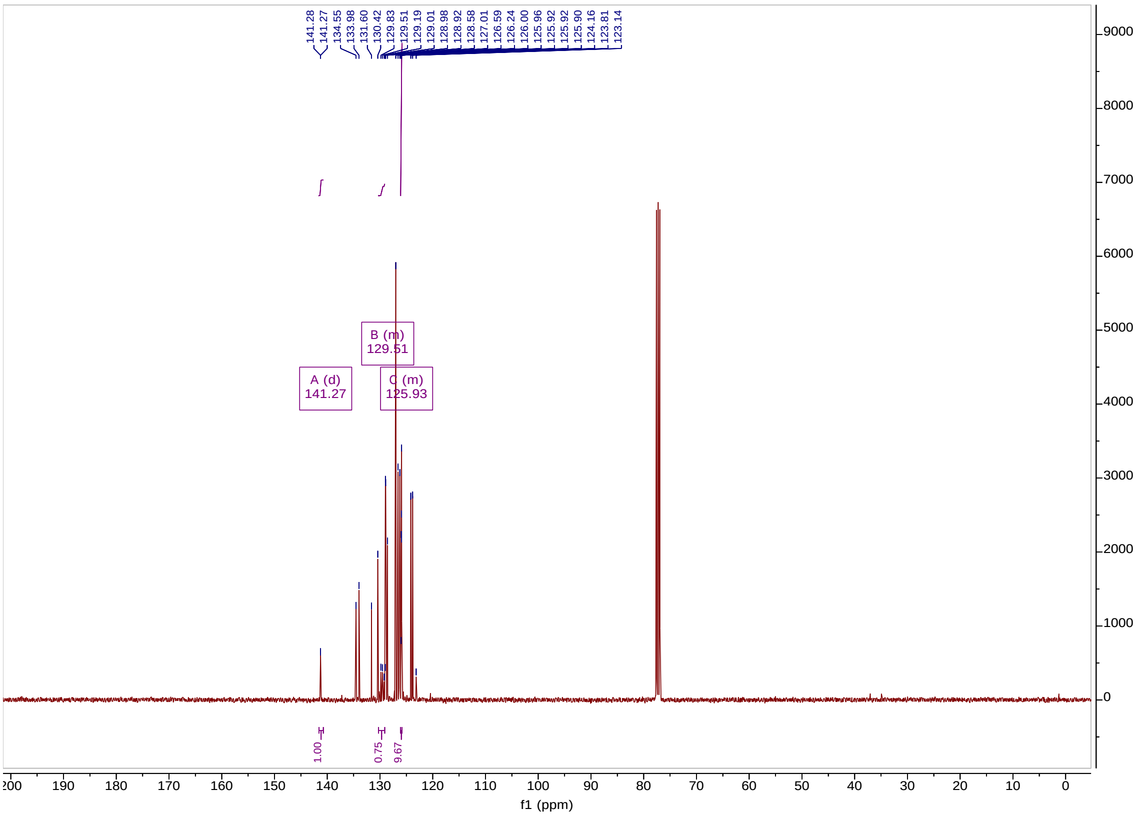

Figure S52. ^{13}C-NMR spectra of compound (**4b**).

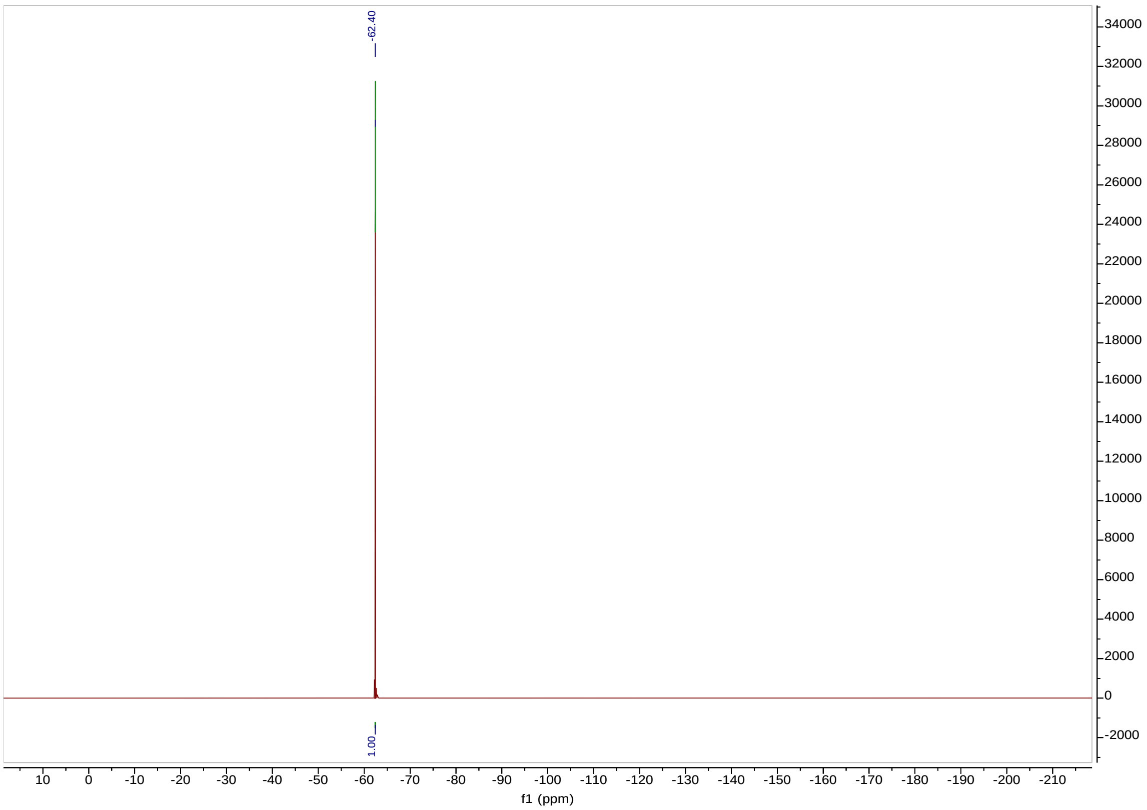

Figure S53. **^{11}F-NMR spectra of compound (**4b**).**

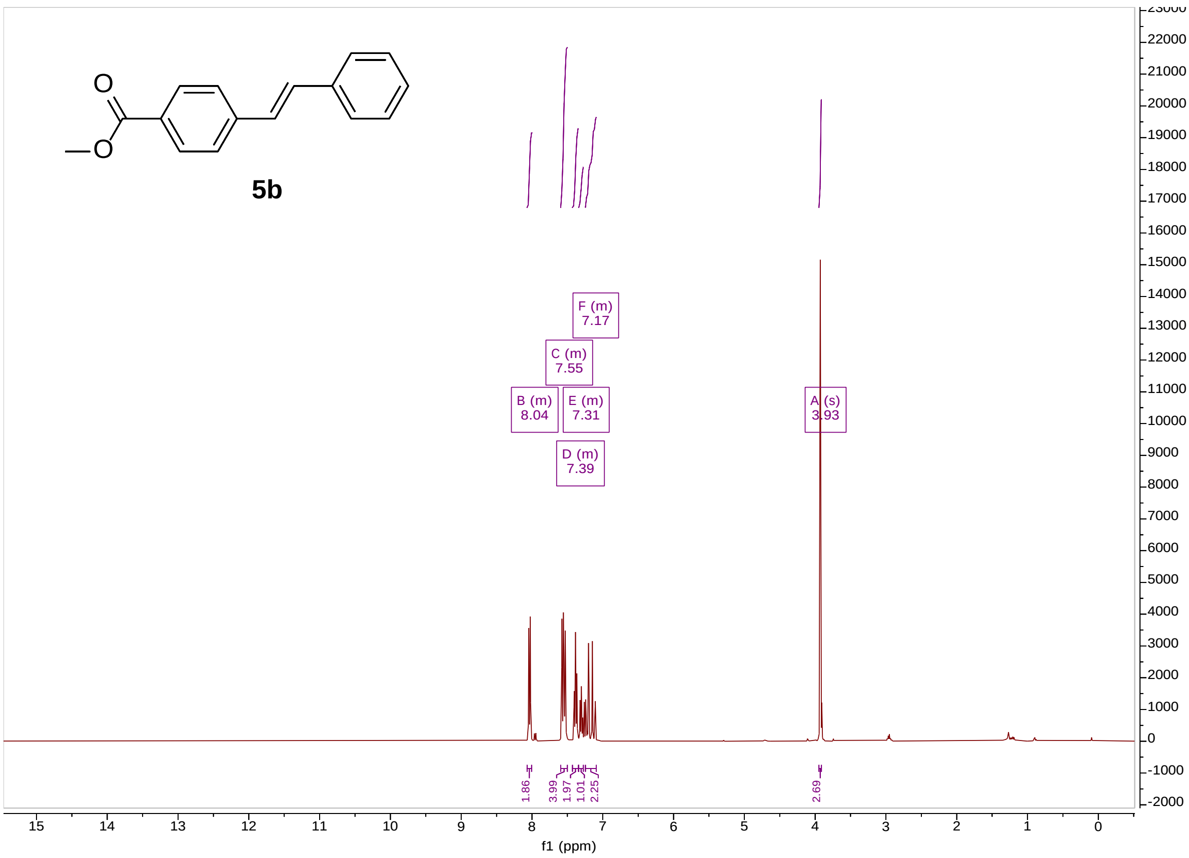

Figure S54. ^{1}H-NMR spectra of compound (**5b**).

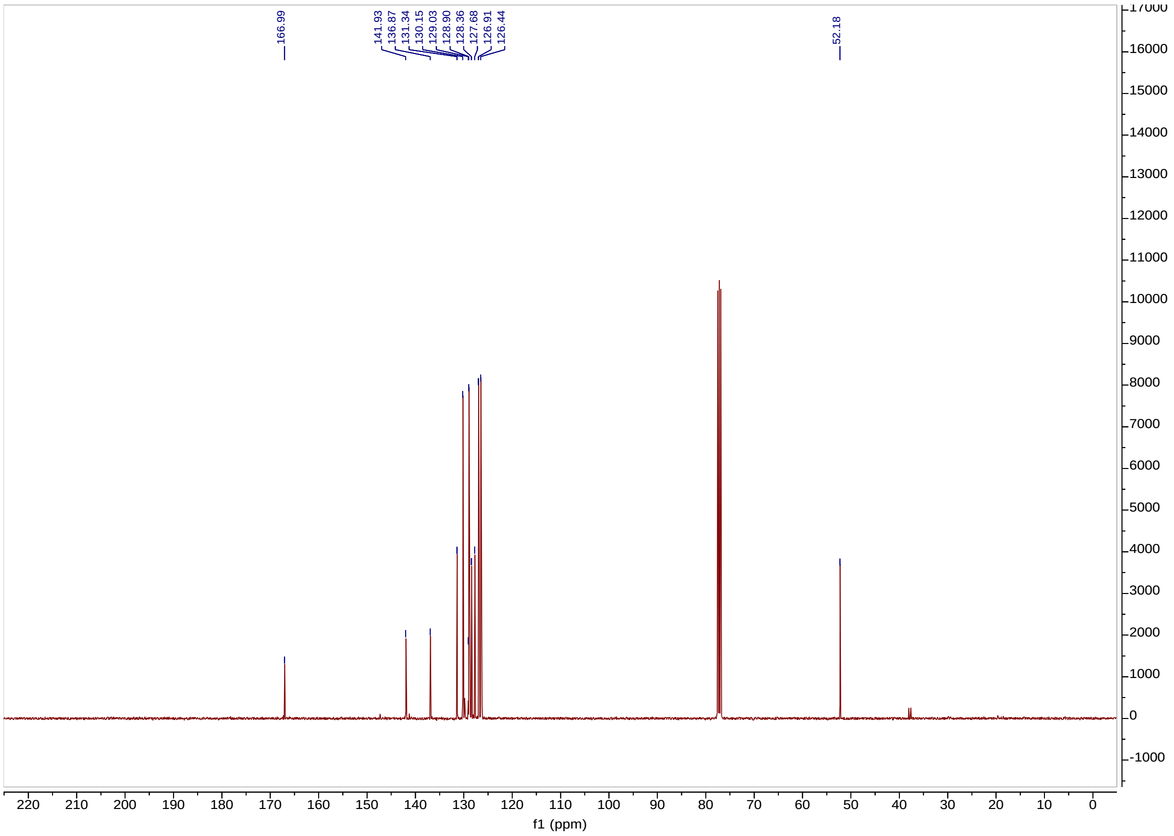

Figure S55. ^{13}C-NMR spectra of compound (**5b**).

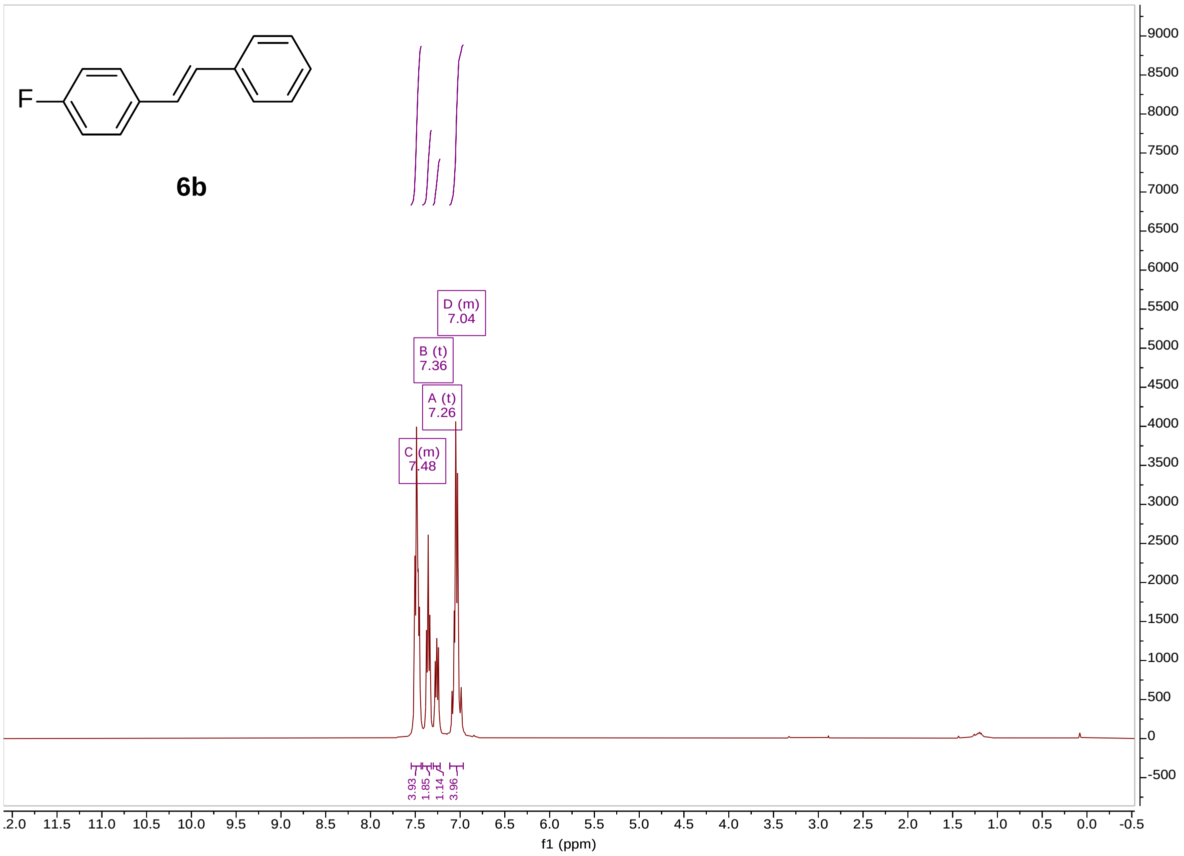

Figure S56. ^{1}H-NMR spectra of compound (**6b**).

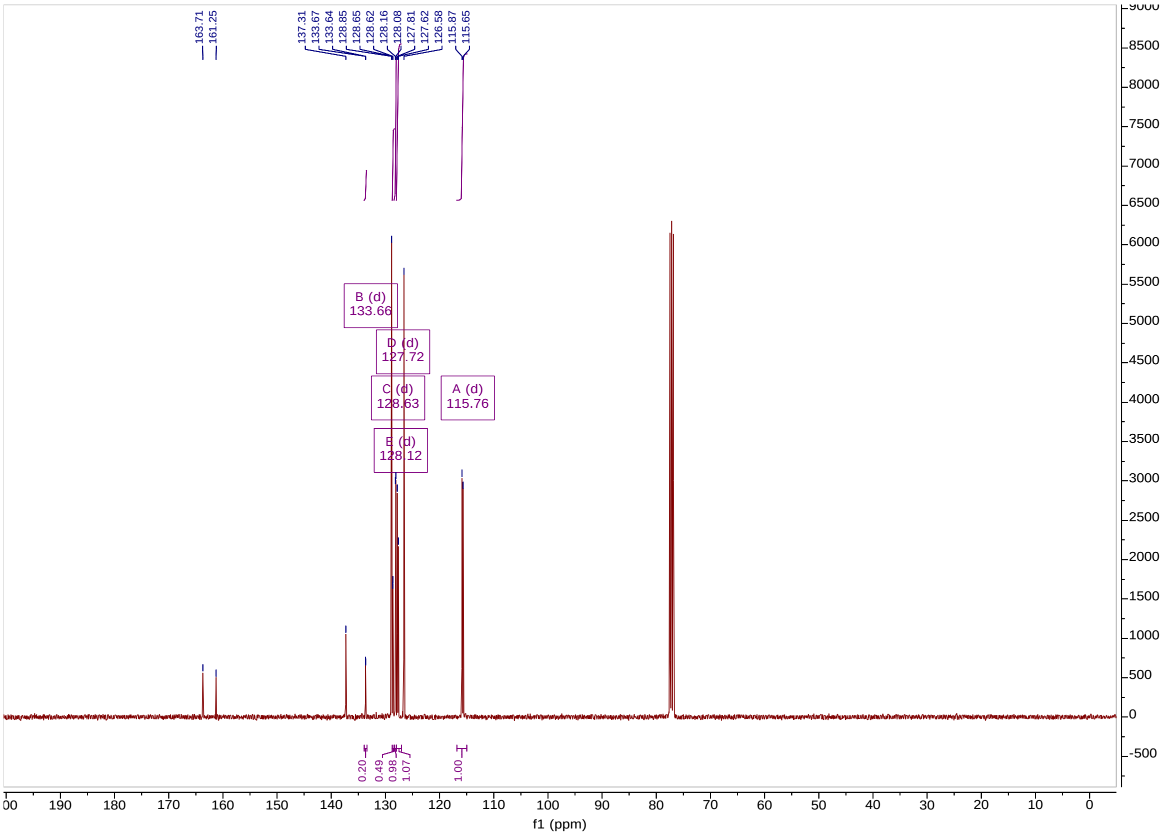

Figure S57. ^{13}C-NMR spectra of compound (**6b**).

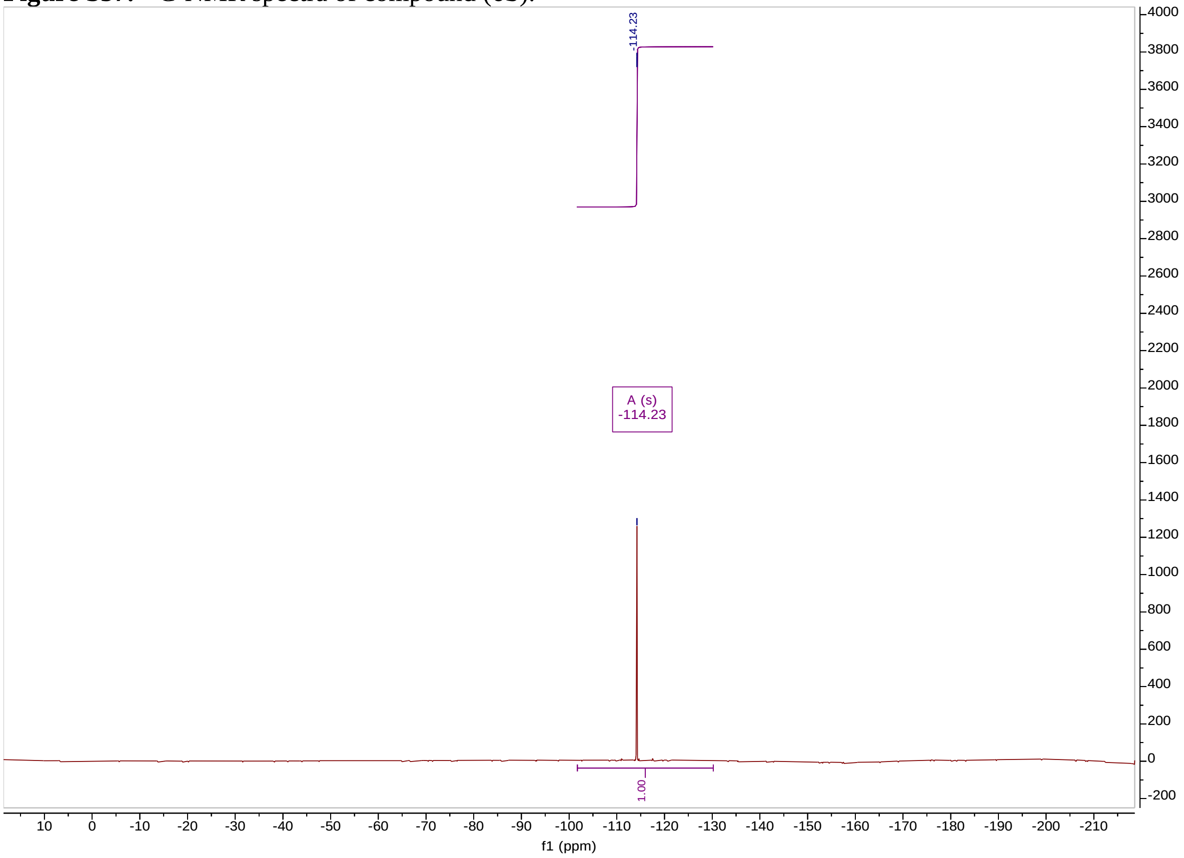

Figure S58. ^{11}F-NMR spectra of compound (**6b**).

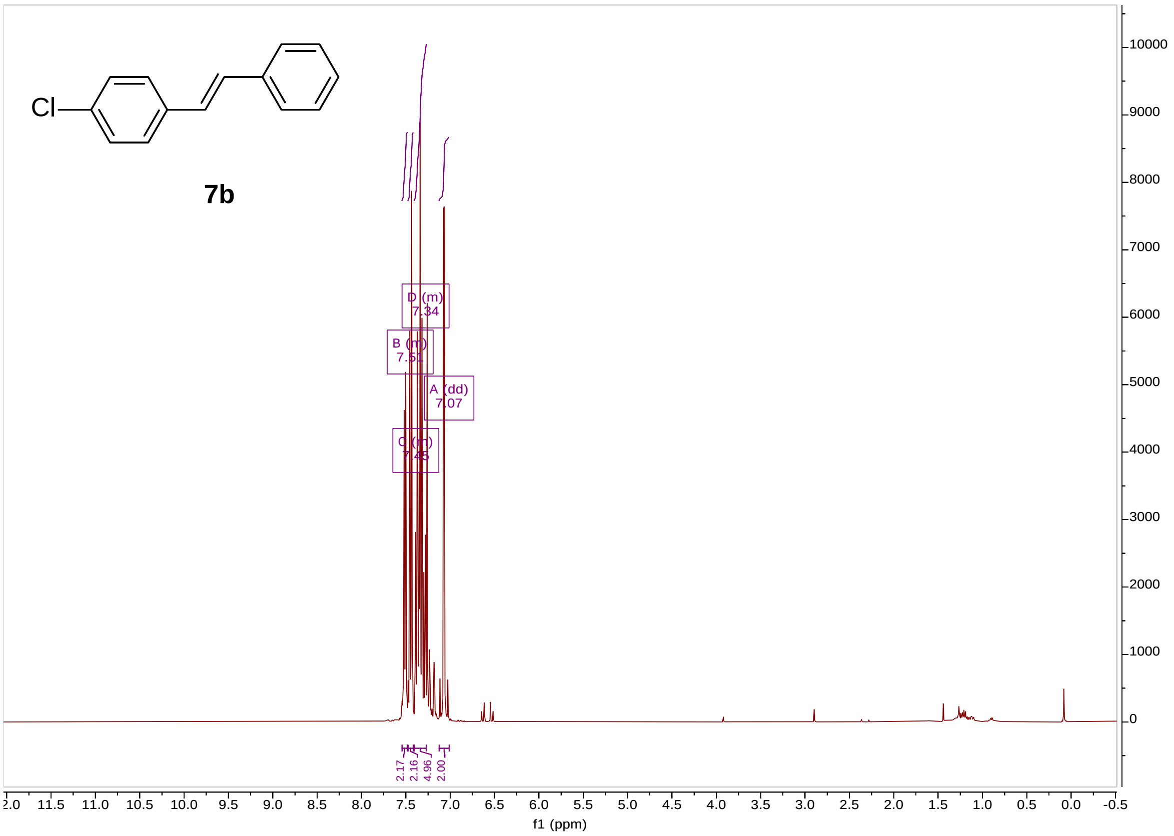

Figure S59. ^{1}H-NMR spectra of compound (**7b**).

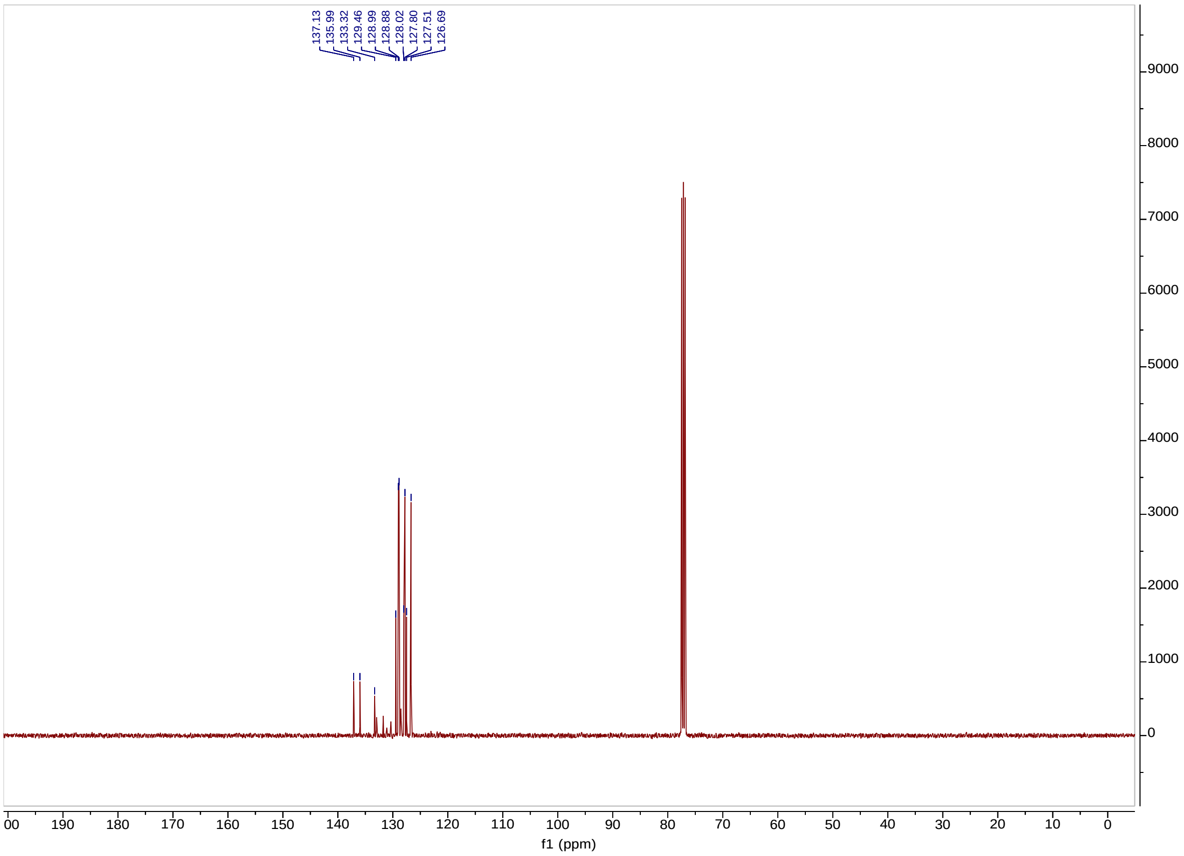

Figure S60. ^{13}C-NMR spectra of compound (**7b**).

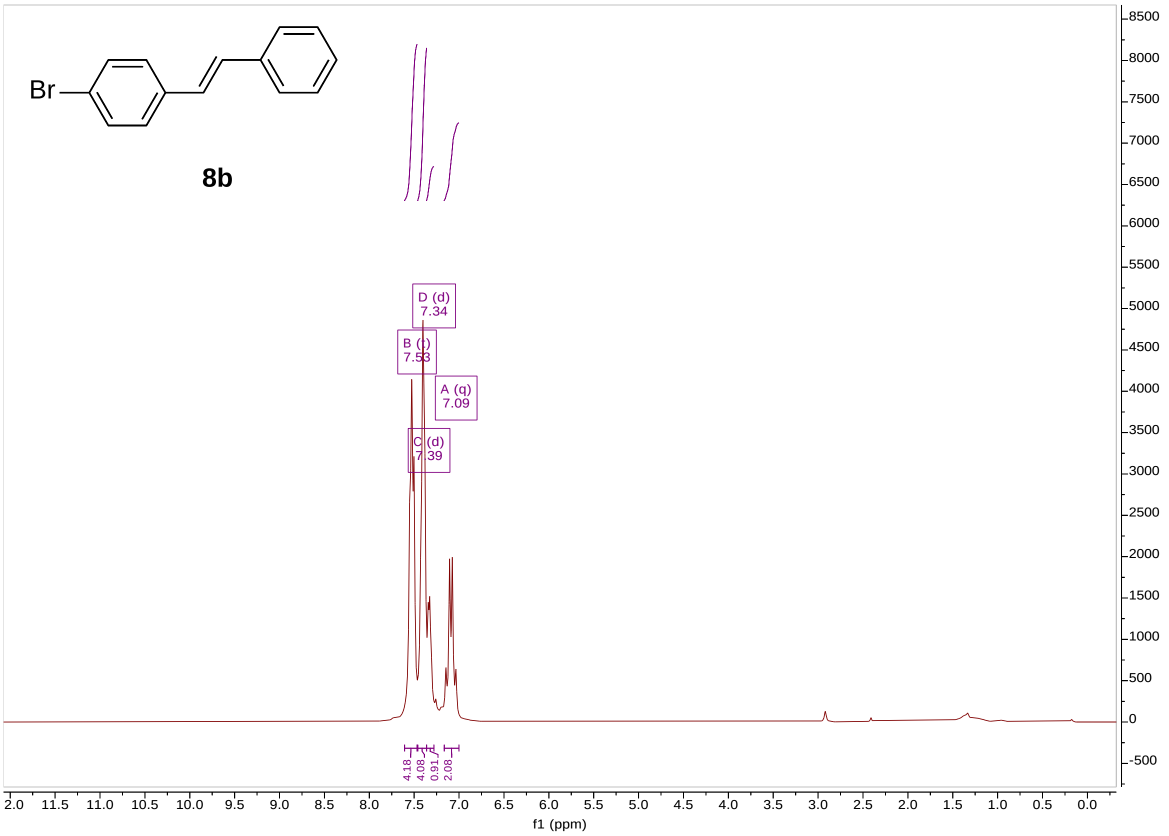

Figure S61. ^{1}H-NMR spectra of compound (**8b**).

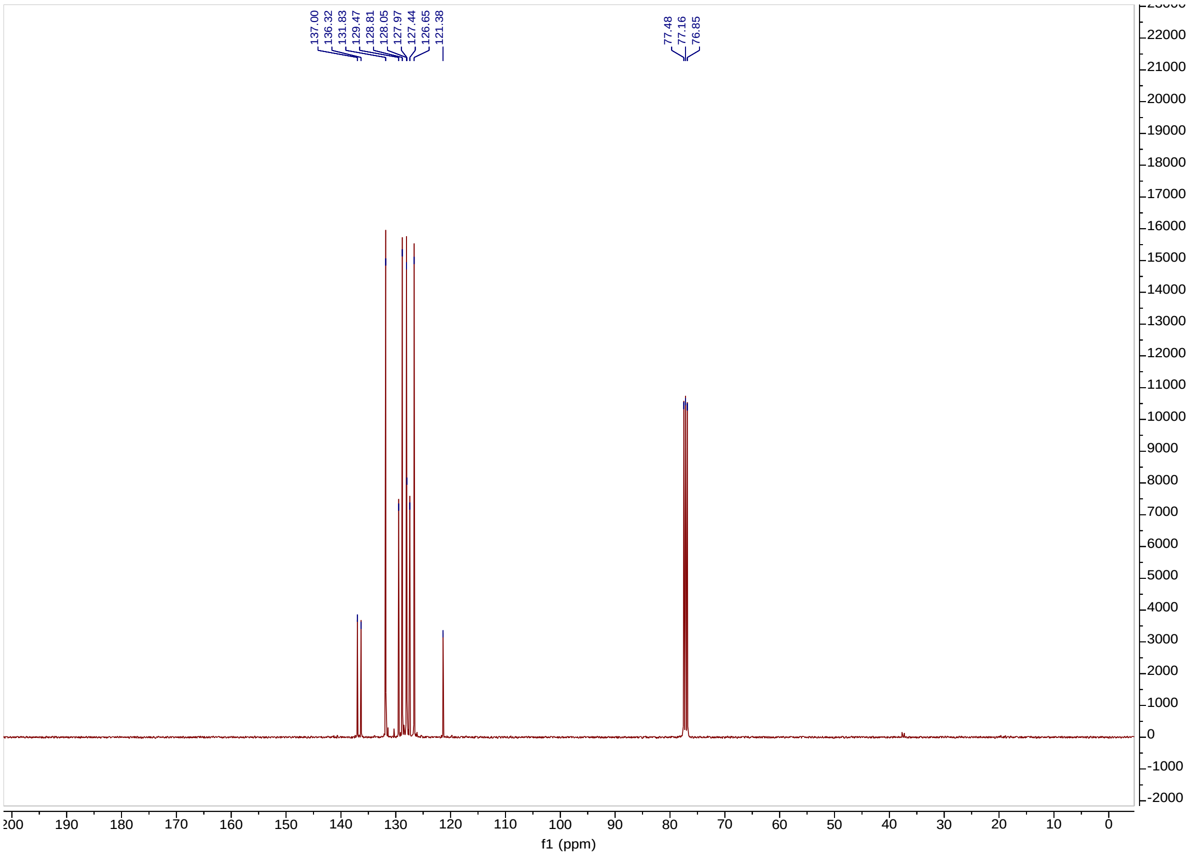

Figure S62. ^{13}C-NMR spectra of compound (**8b**).

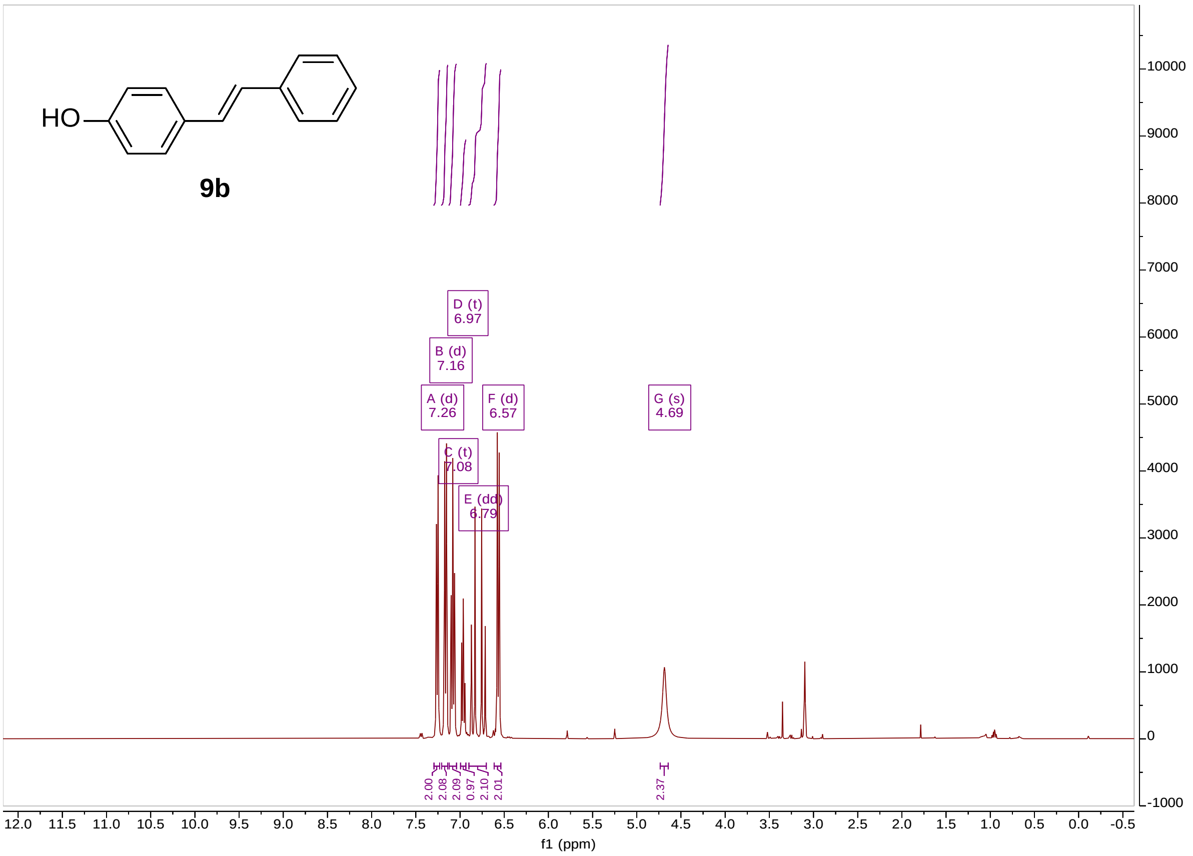

Figure S63. ^{1}H-NMR spectra of compound (**9b**).

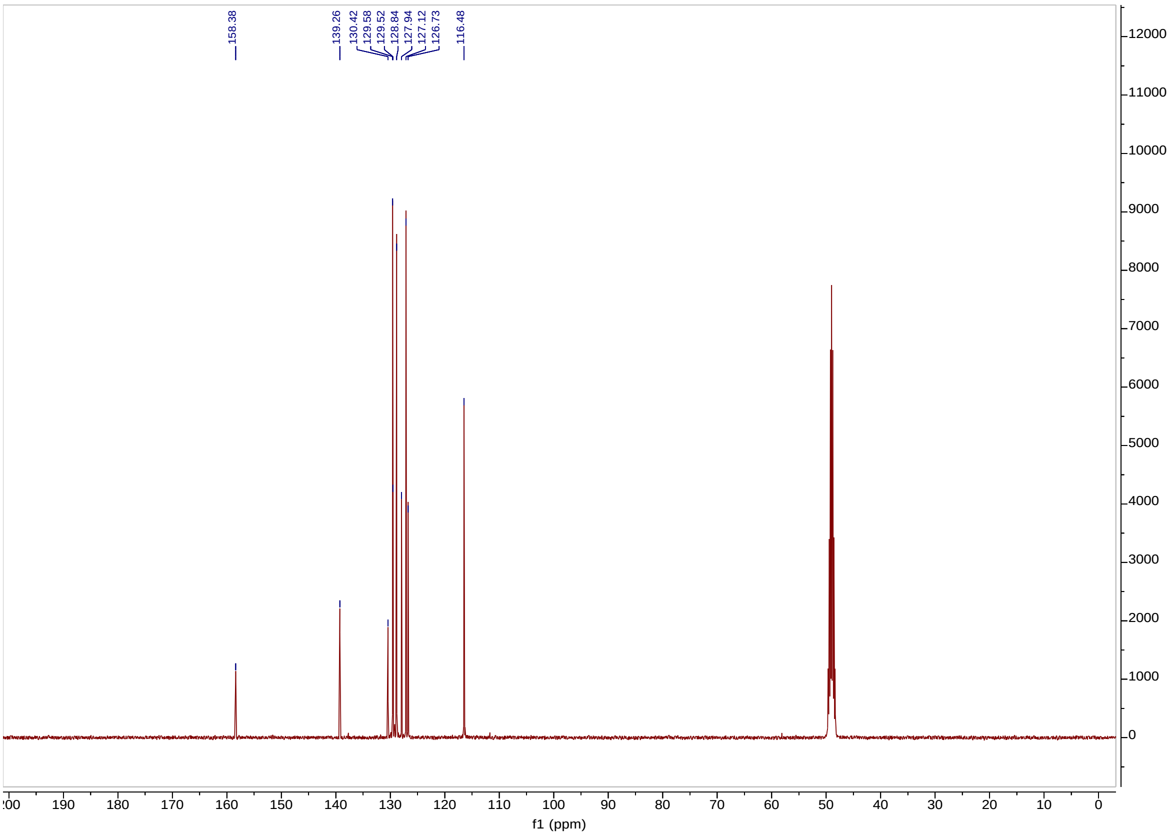

Figure S64. ^{13}C-NMR spectra of compound (**9b**).

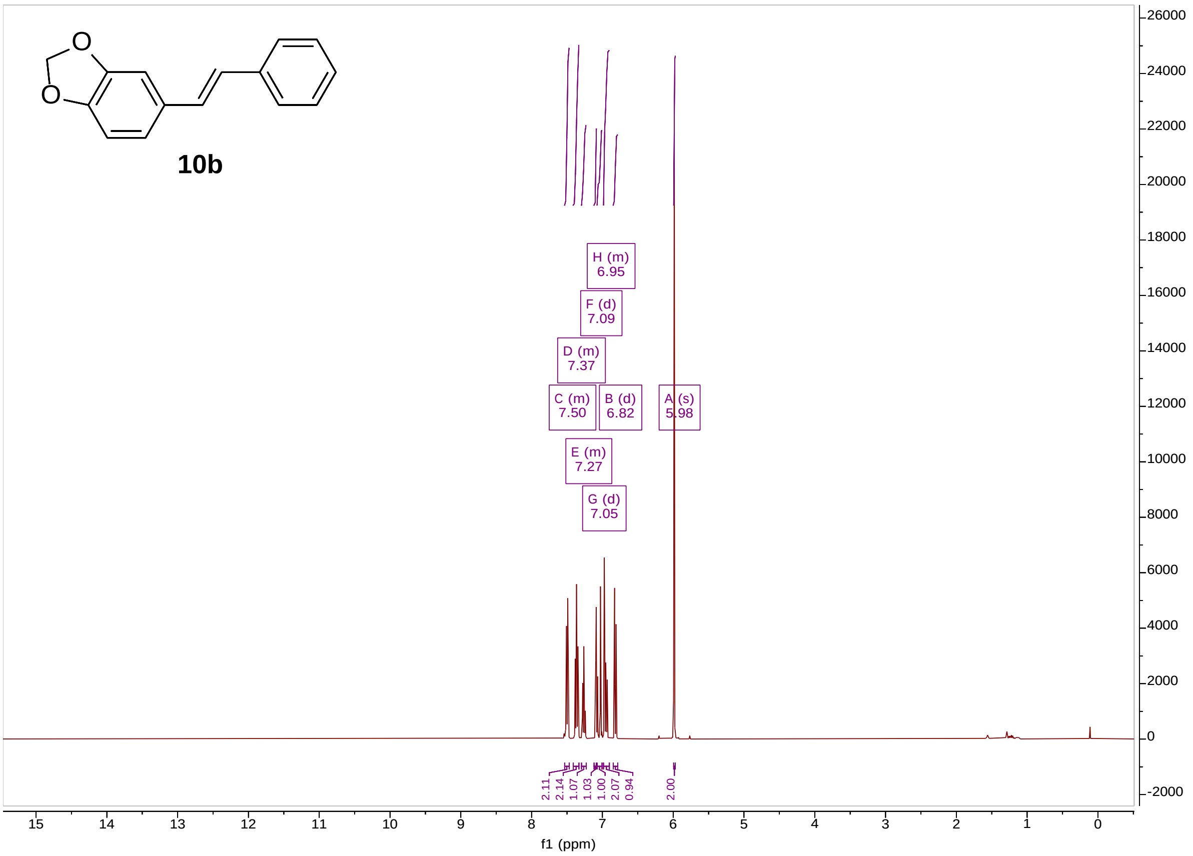

Figure S65. ^{1}H-NMR spectra of compound (**10b**).

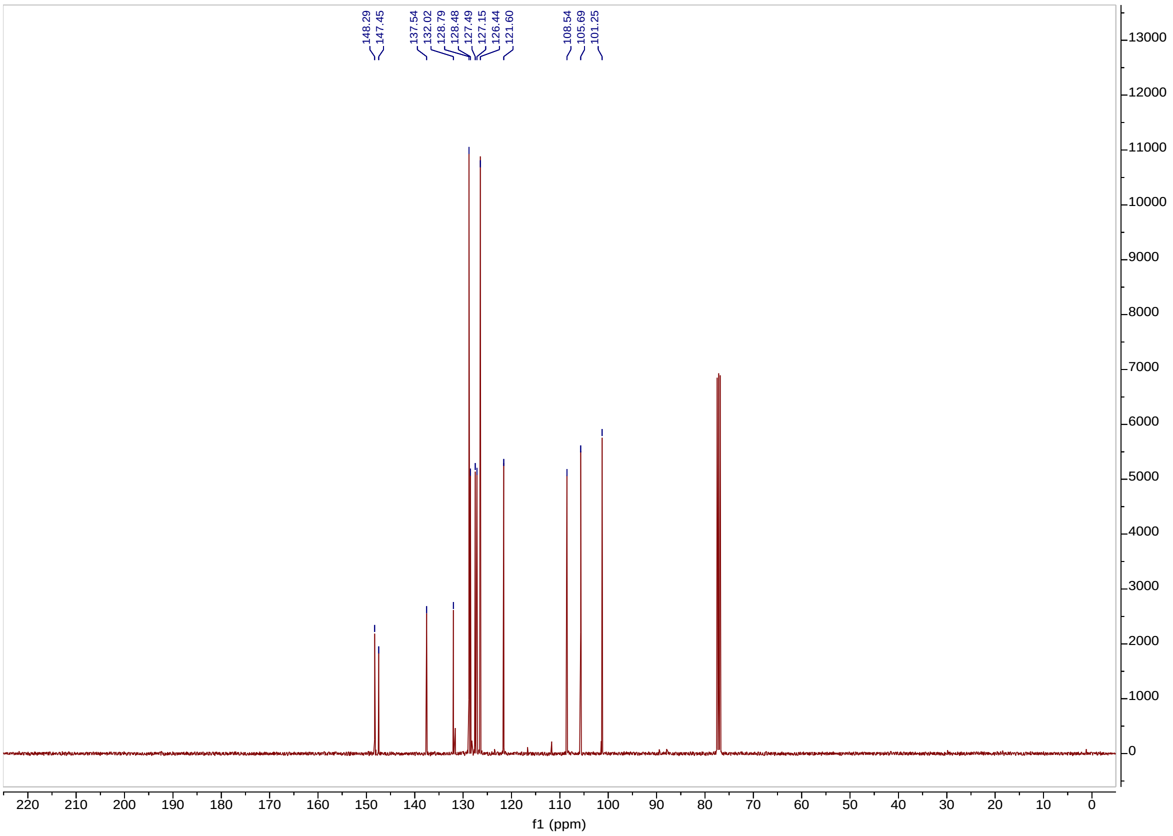

Figure S66. ^{13}C-NMR spectra of compound (**10b**).

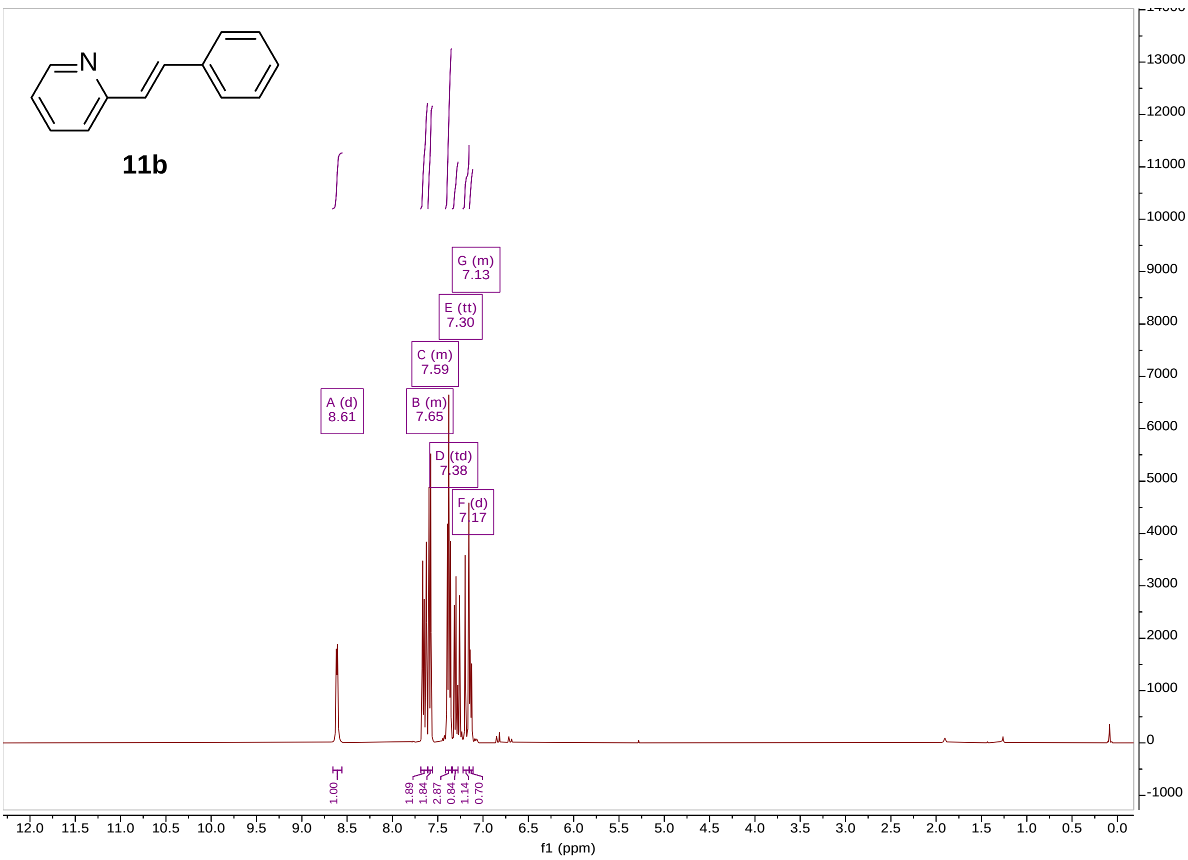

Figure S67. ^{1}H-NMR spectra of compound (**11b**).

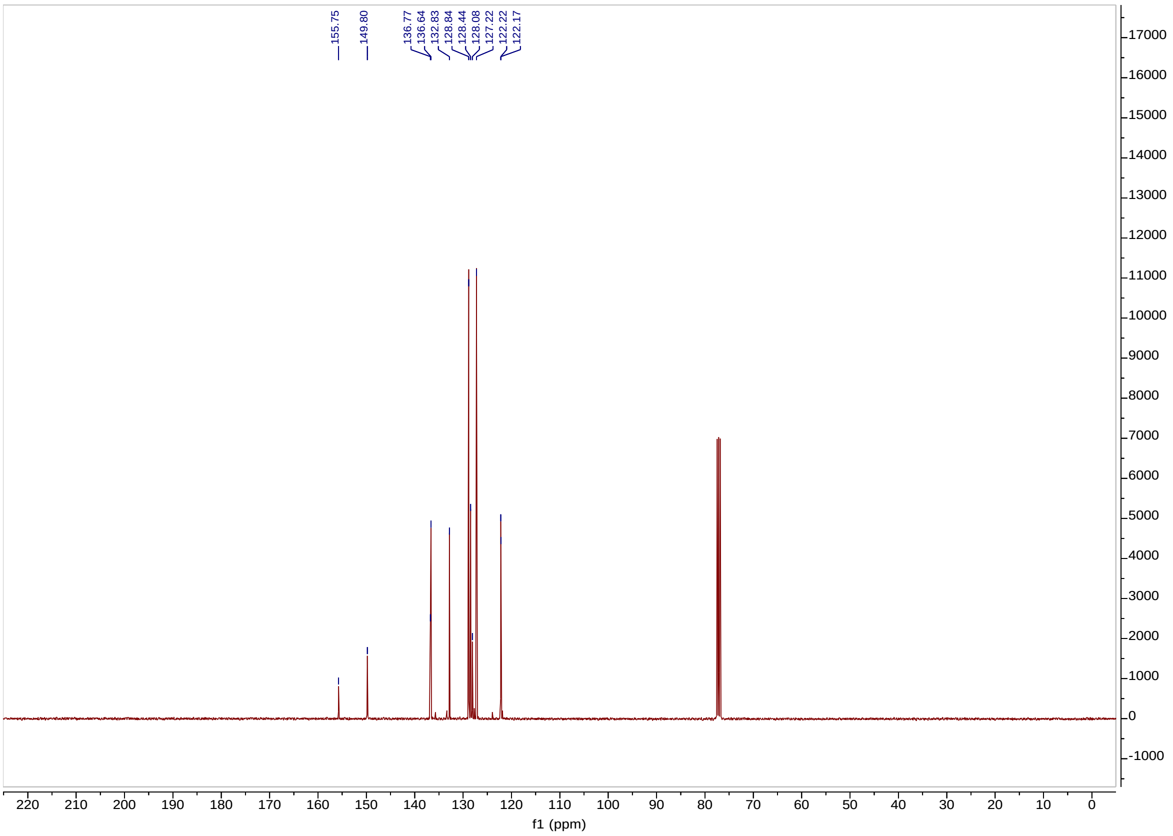

Figure S68. ^{13}C-NMR spectra of compound (**11b**).

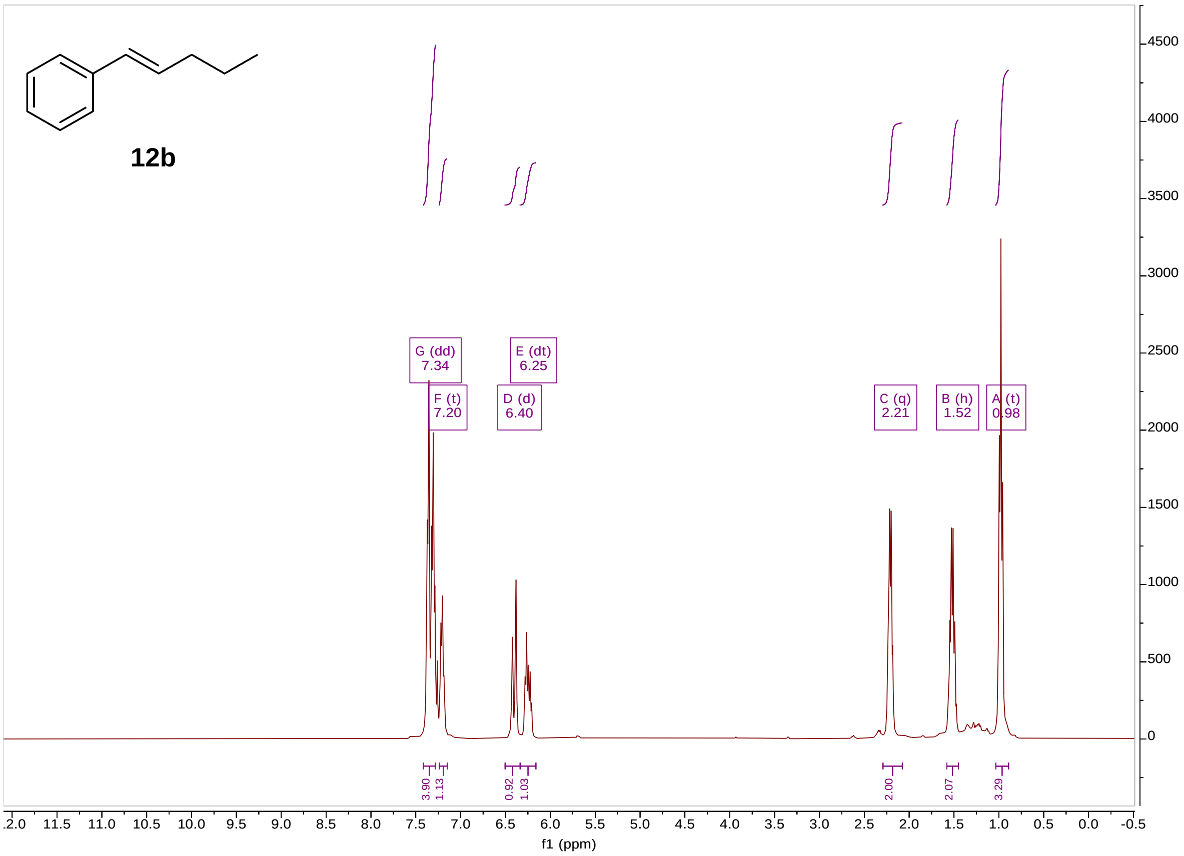

Figure S69. ^{1}H-NMR spectra of compound (**12b**).

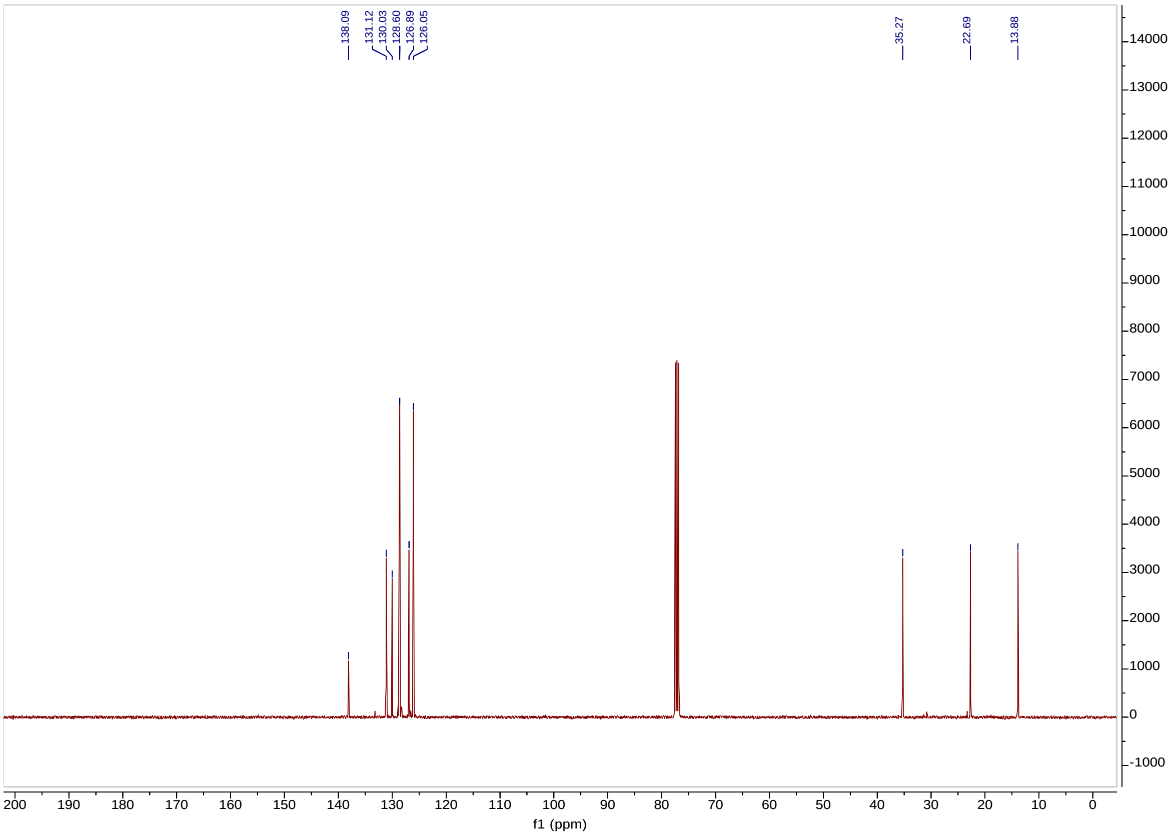

Figure S70. ^{13}C-NMR spectra of compound (**12b**).

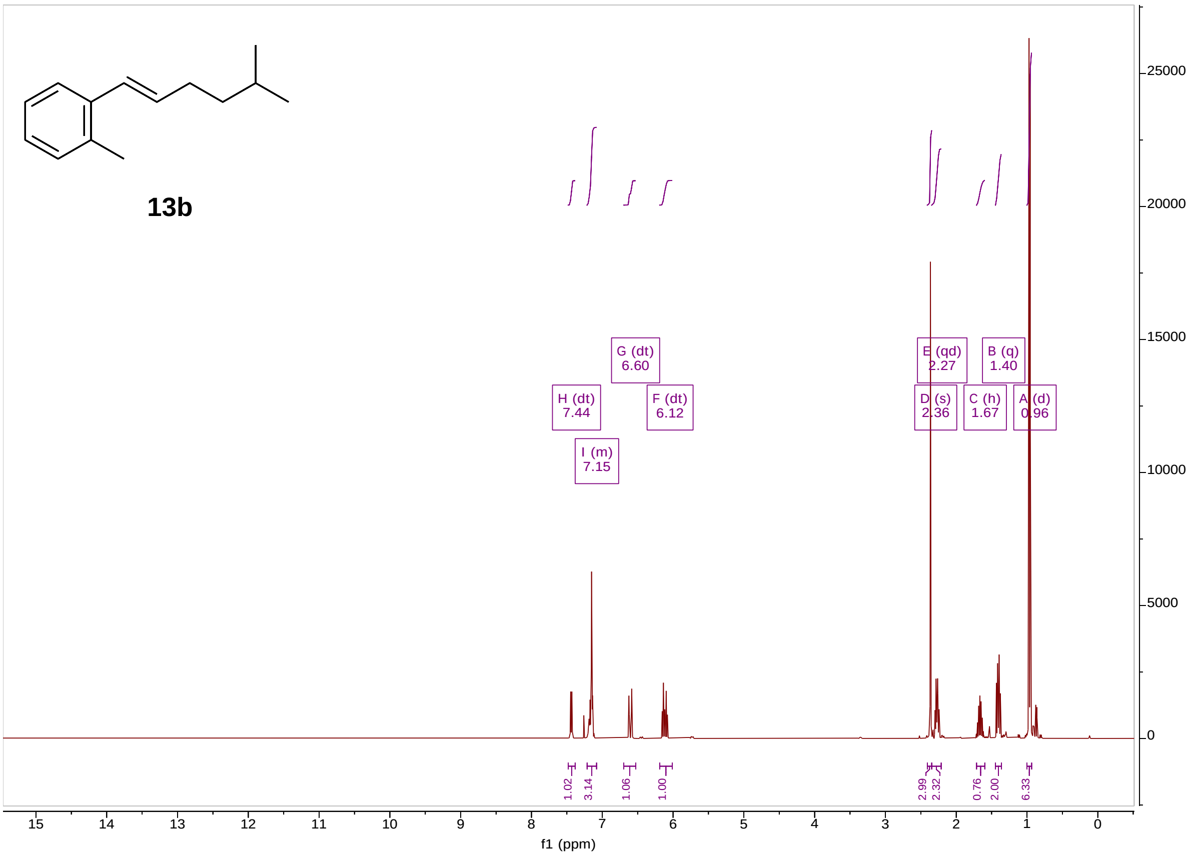

Figure S71. ^{1}H-NMR spectra of compound (**13b**).

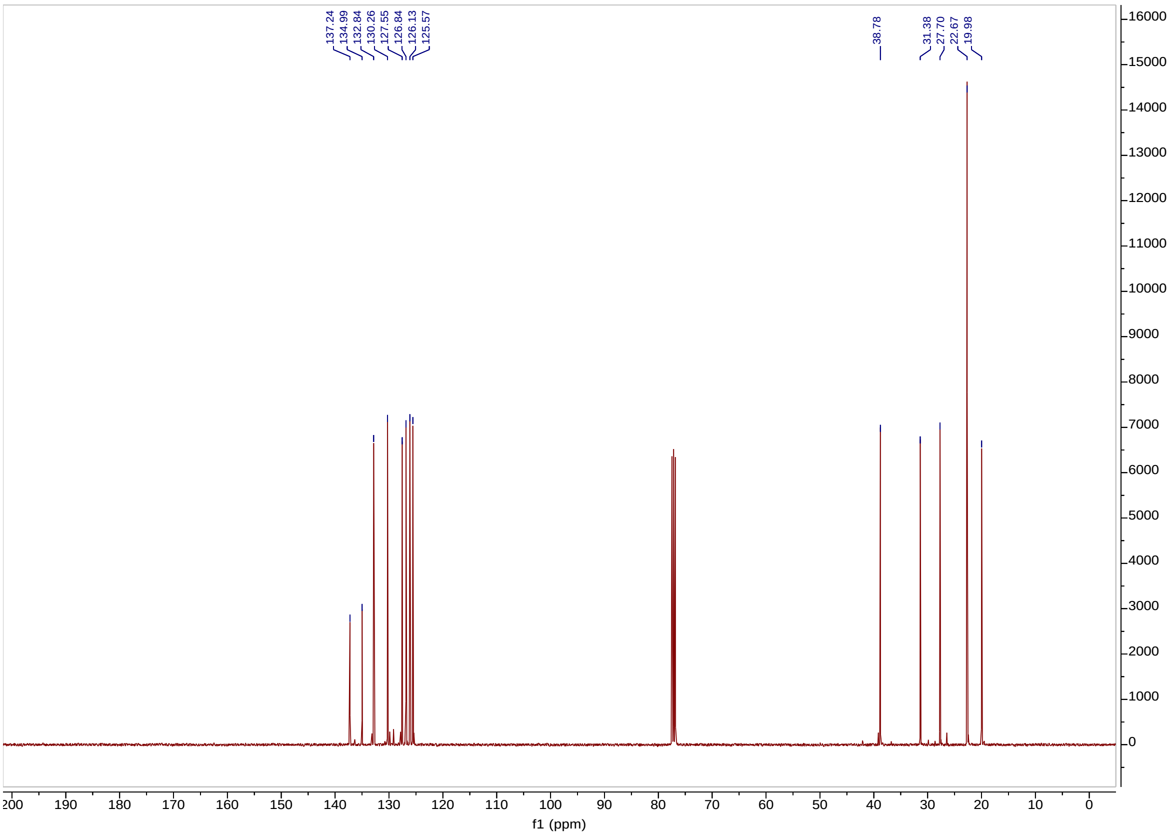

Figure S72. ^{13}C-NMR spectra of compound (**13b**).

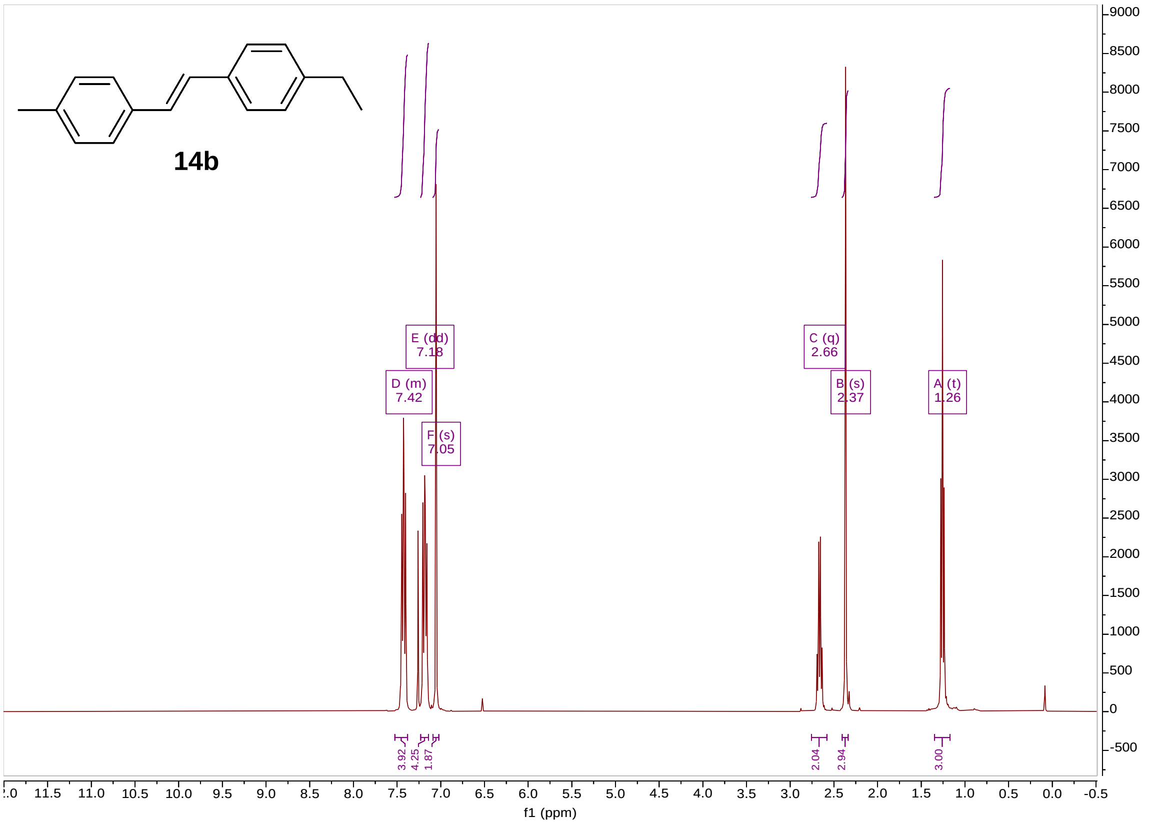

Figure S73. ^{1}H-NMR spectra of compound (**14b**).

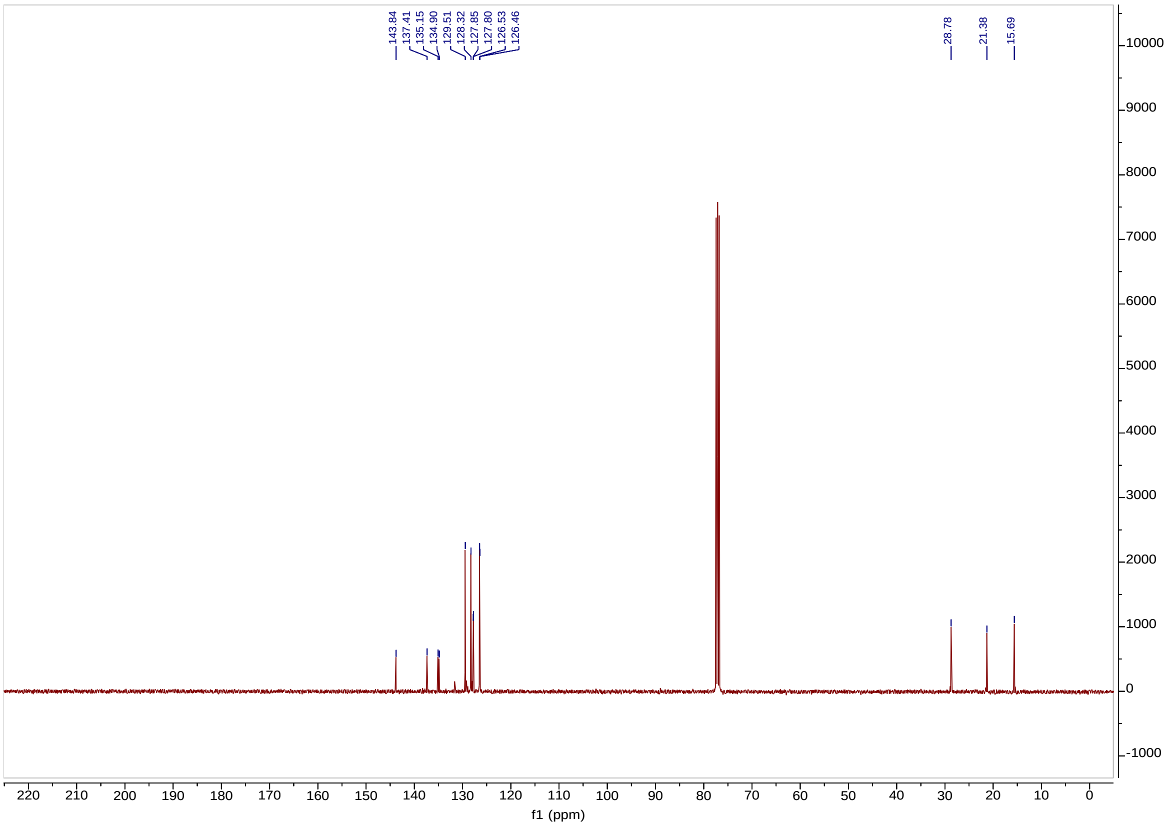

Figure S74. ^{13}C-NMR spectra of compound (**14b**).

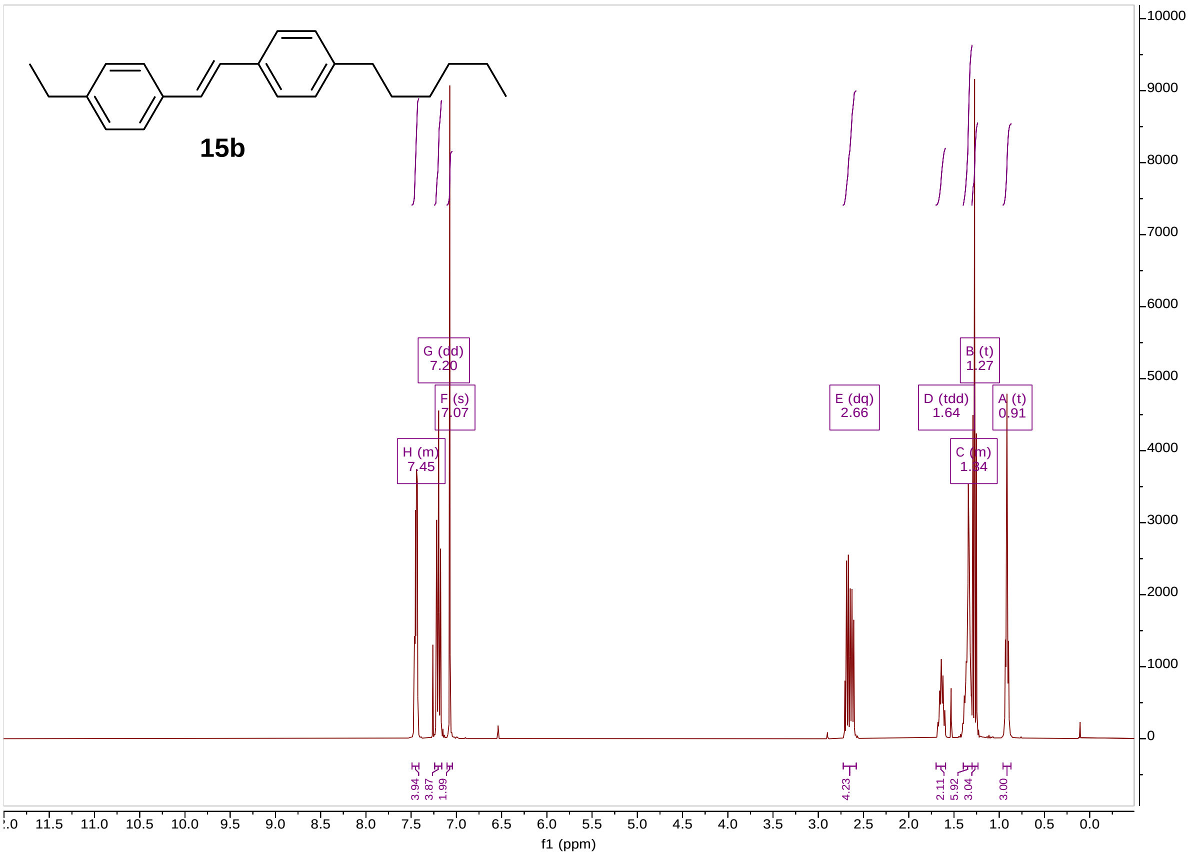

Figure S75. ^{1}H-NMR spectra of compound (**15b**).

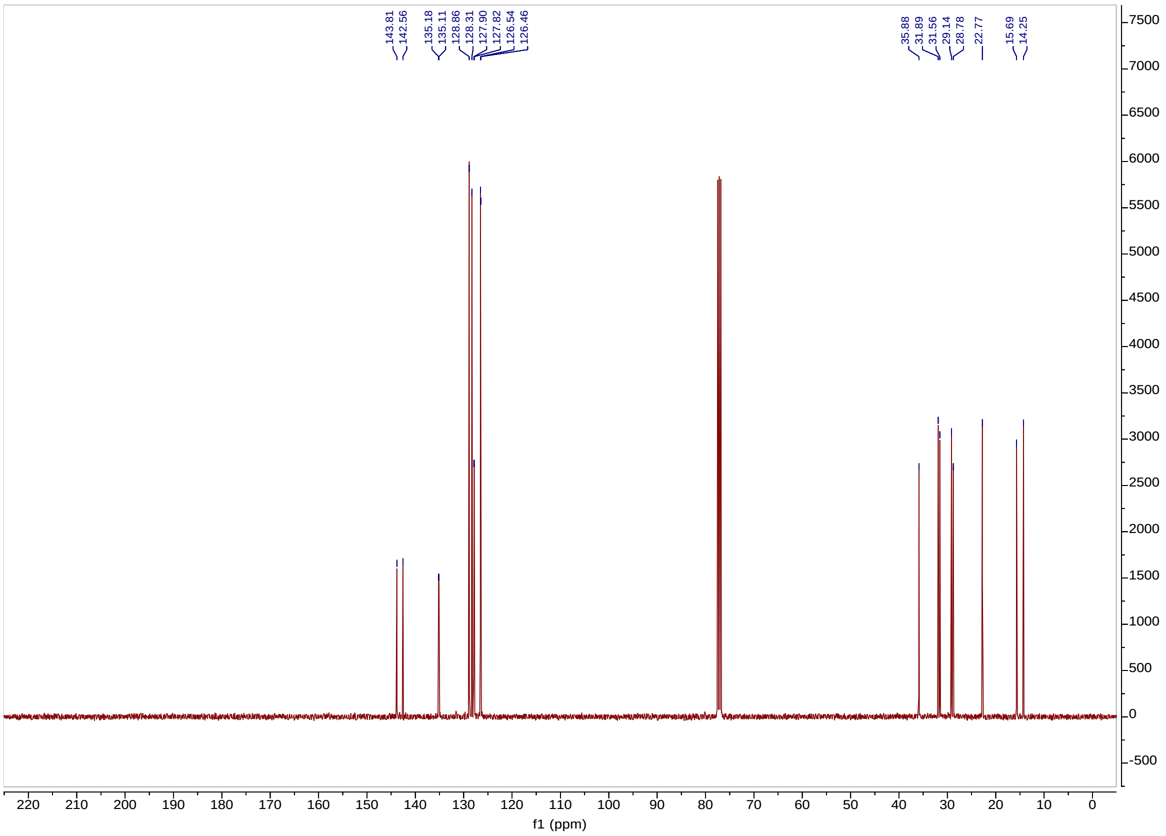

Figure S76. ^{13}C-NMR spectra of compound (**15b**).

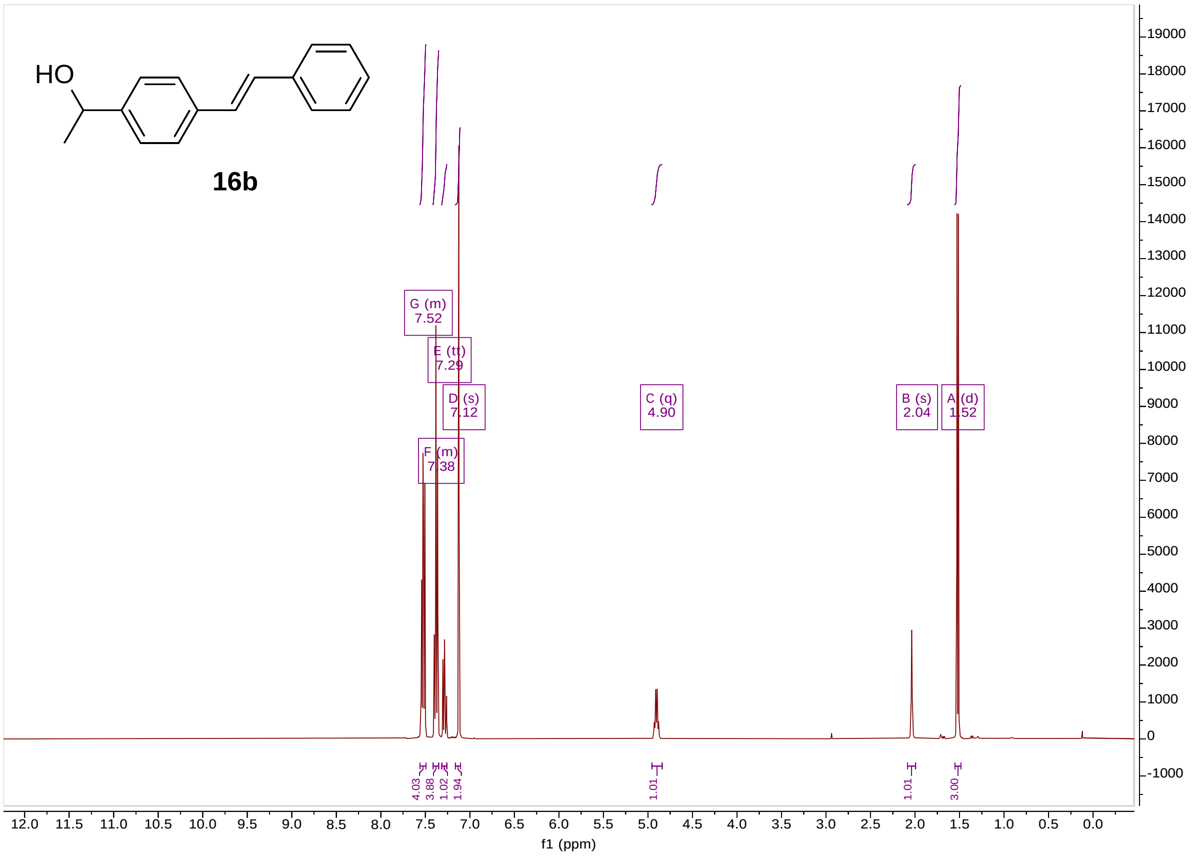

Figure S77. ^{1}H-NMR spectra of compound (**16b**).

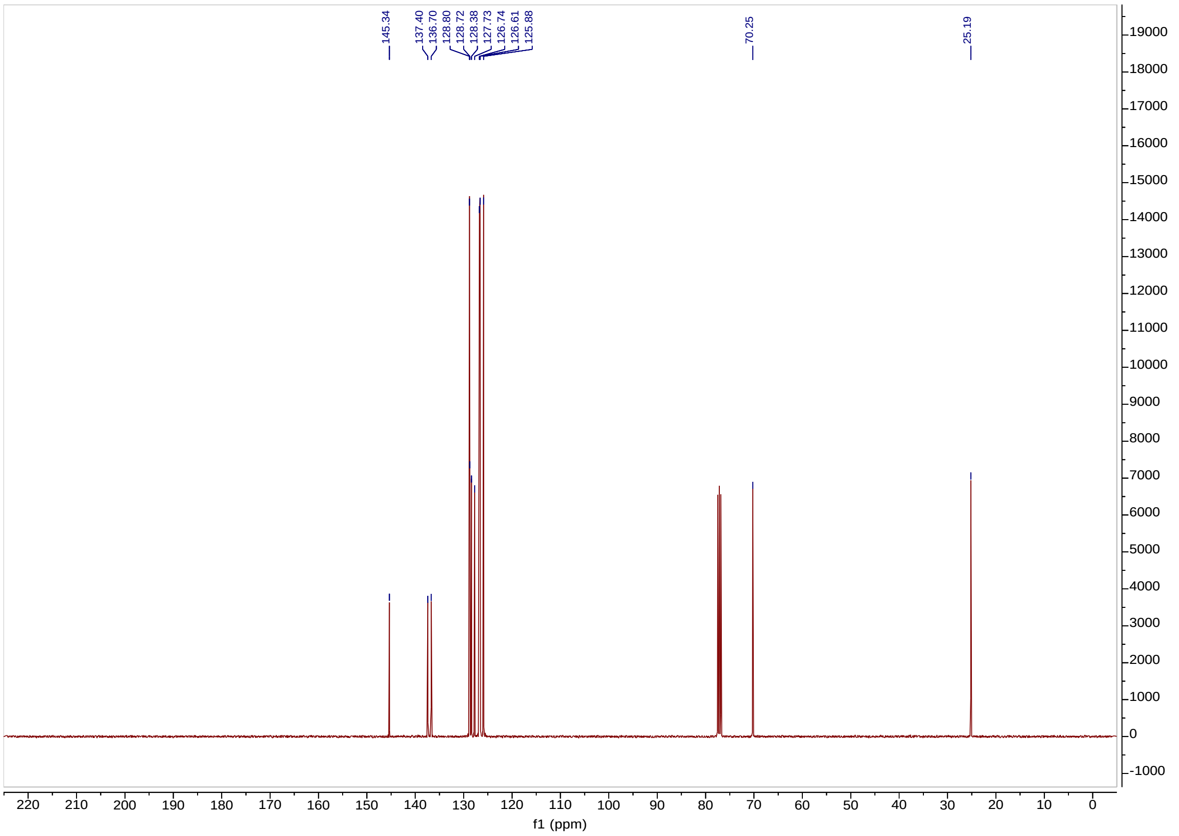

Figure S78. ^{13}C-NMR spectra of compound (**16b**).

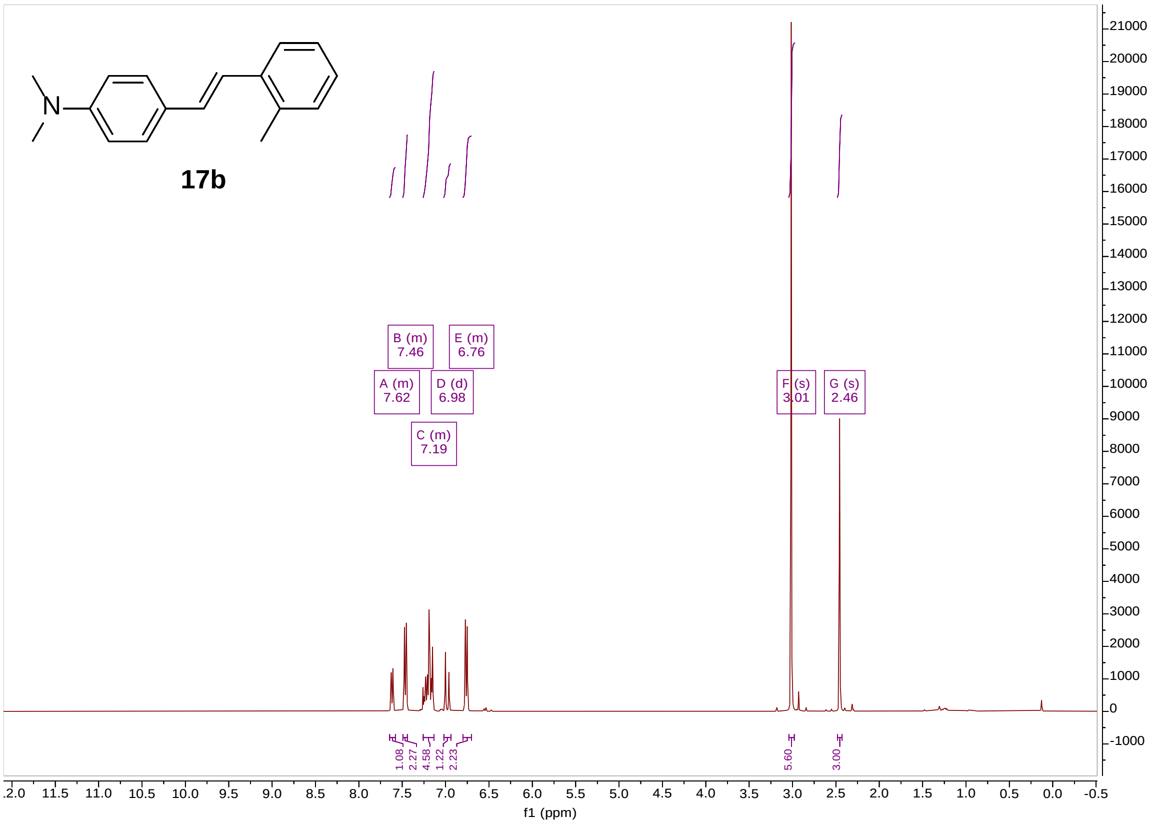

Figure S79. ^{1}H-NMR spectra of compound (**17b**).

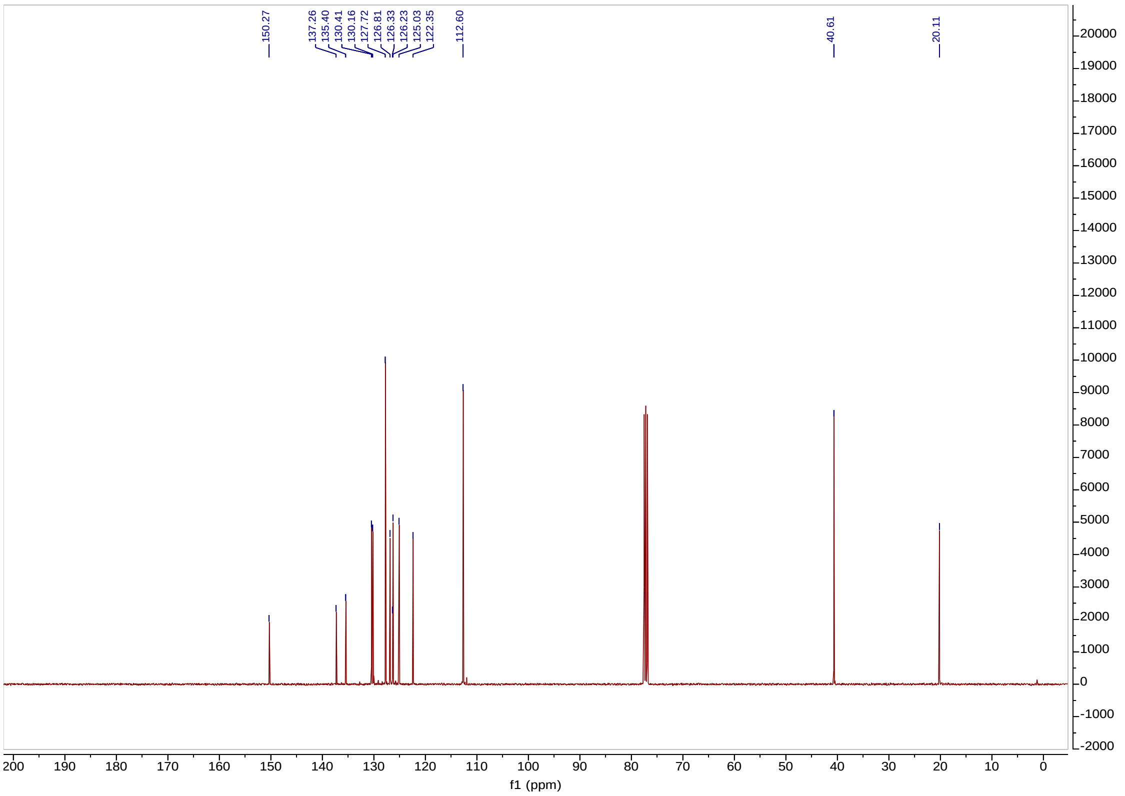

Figure S80. ^{13}C-NMR spectra of compound (**17b**).

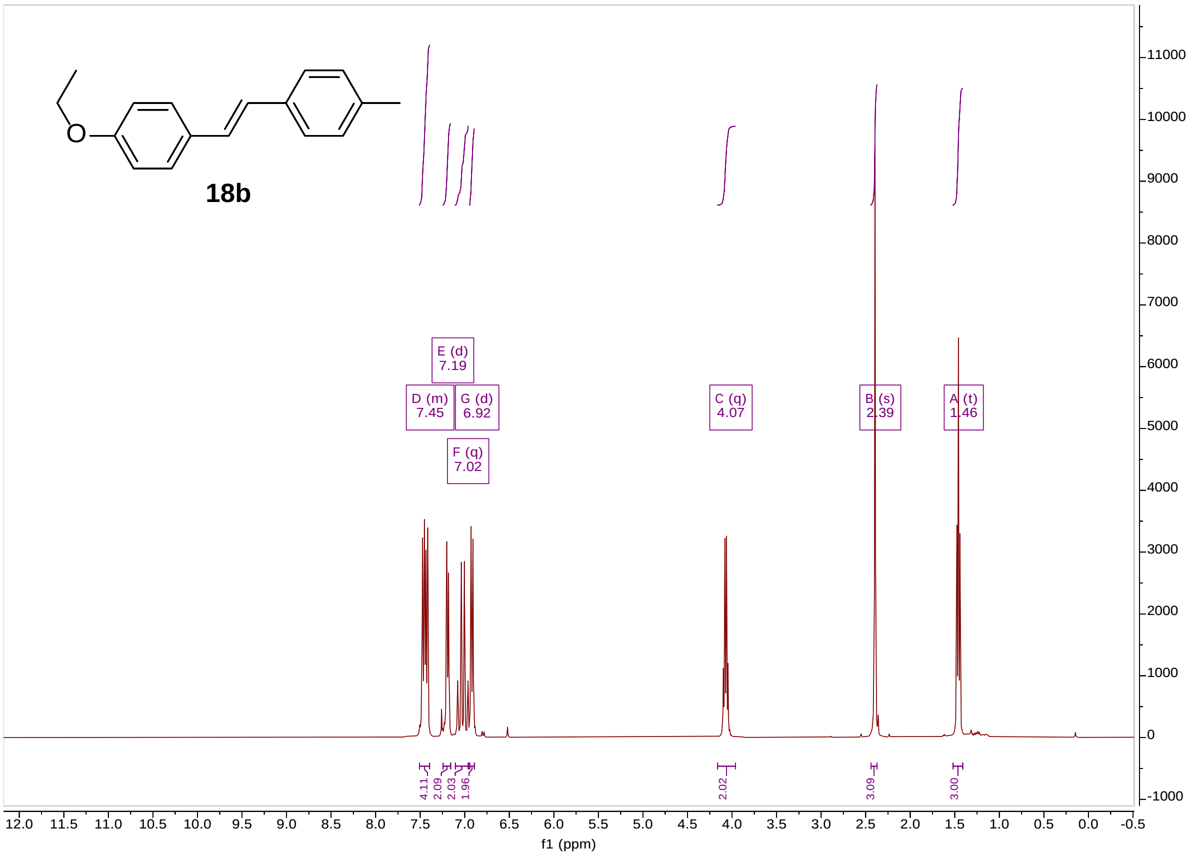

Figure S81. ^{1}H-NMR spectra of compound (**18b**).

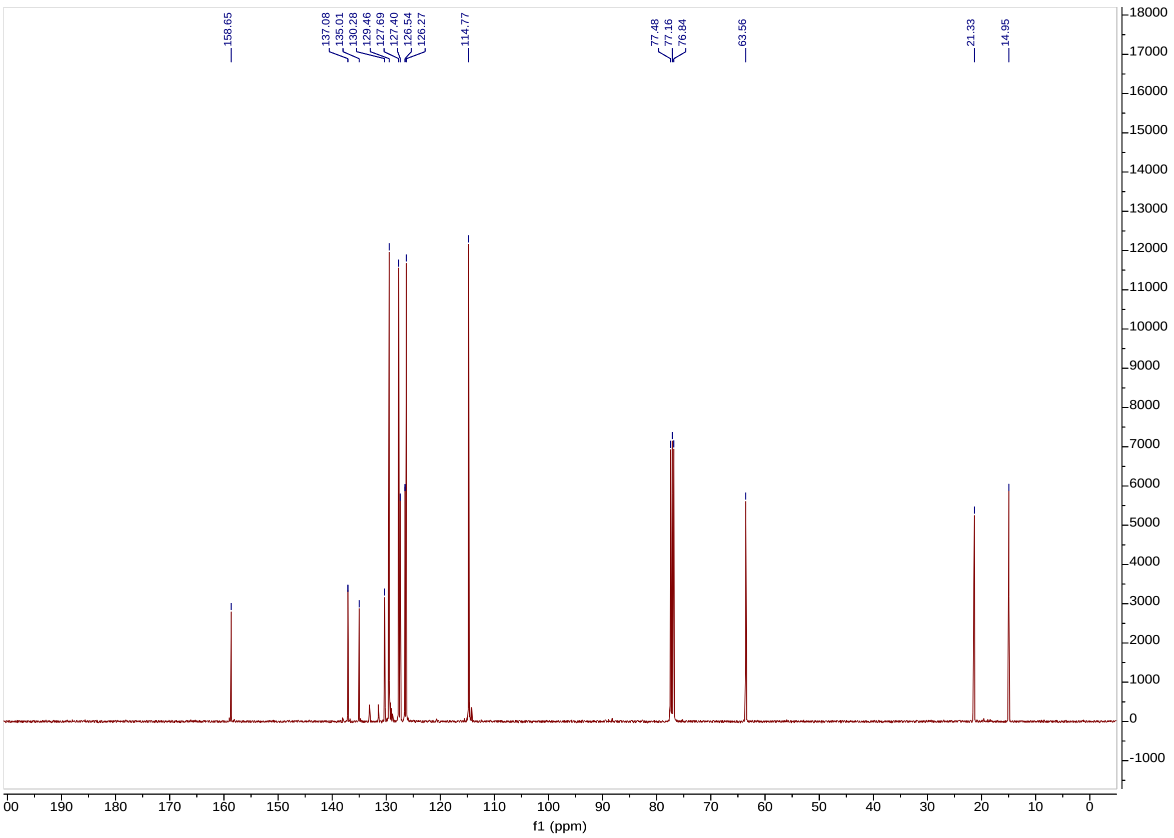

Figure S82. ^{13}C-NMR spectra of compound (**18b**).

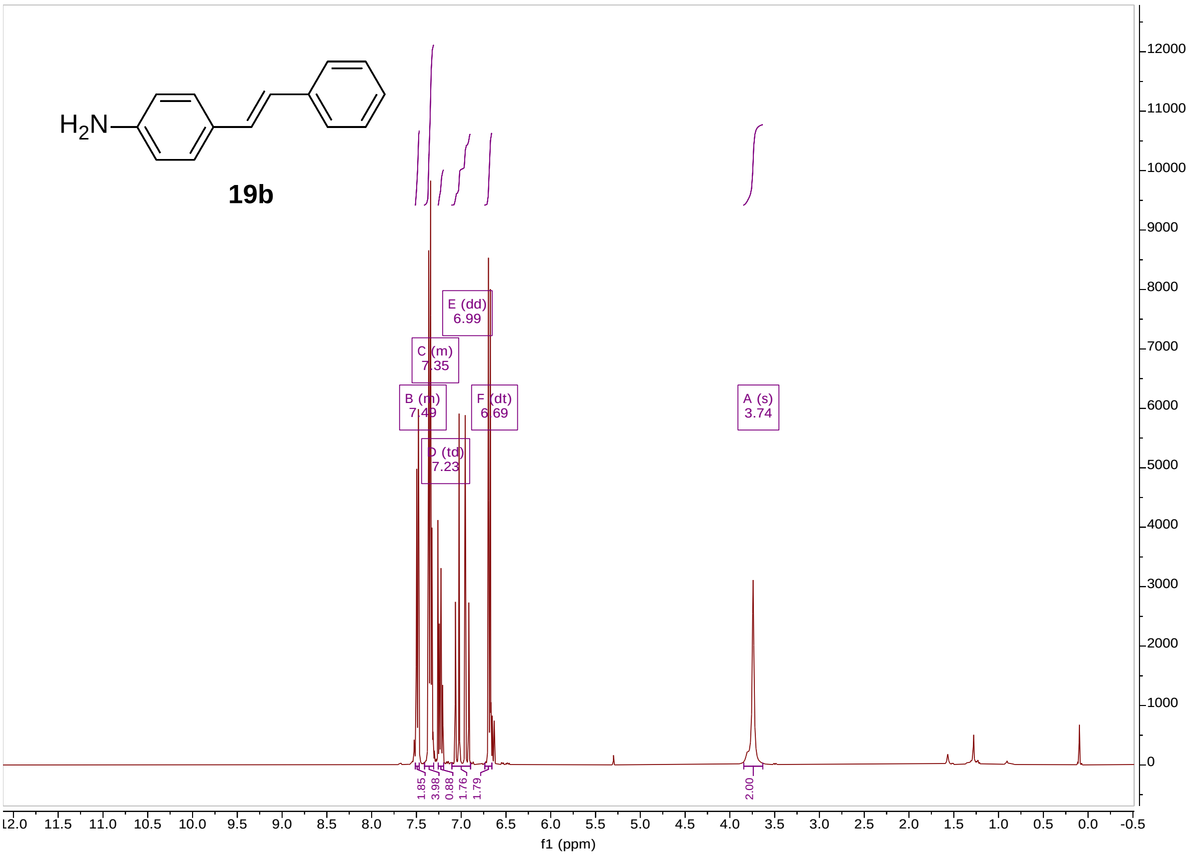

Figure S83. ^{1}H-NMR spectra of compound (**19b**).

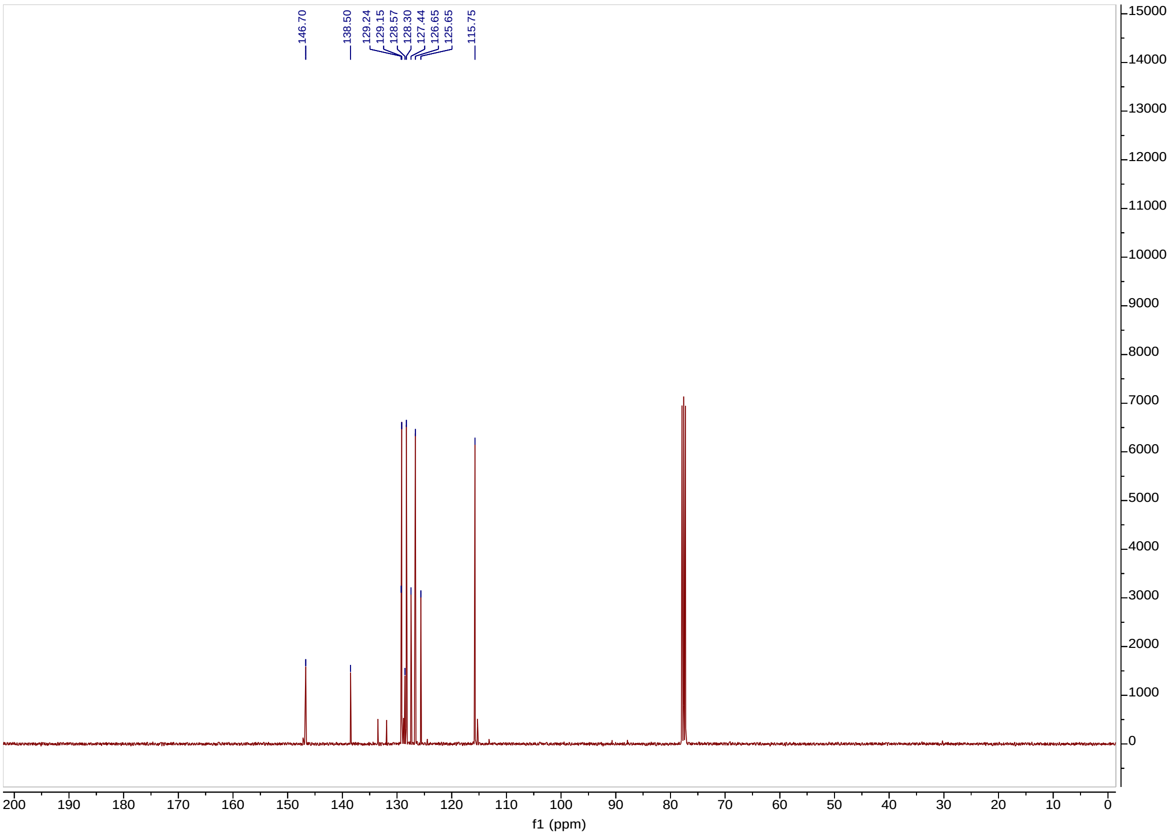

Figure S84. ^{13}C-NMR spectra of compound (**19b**).

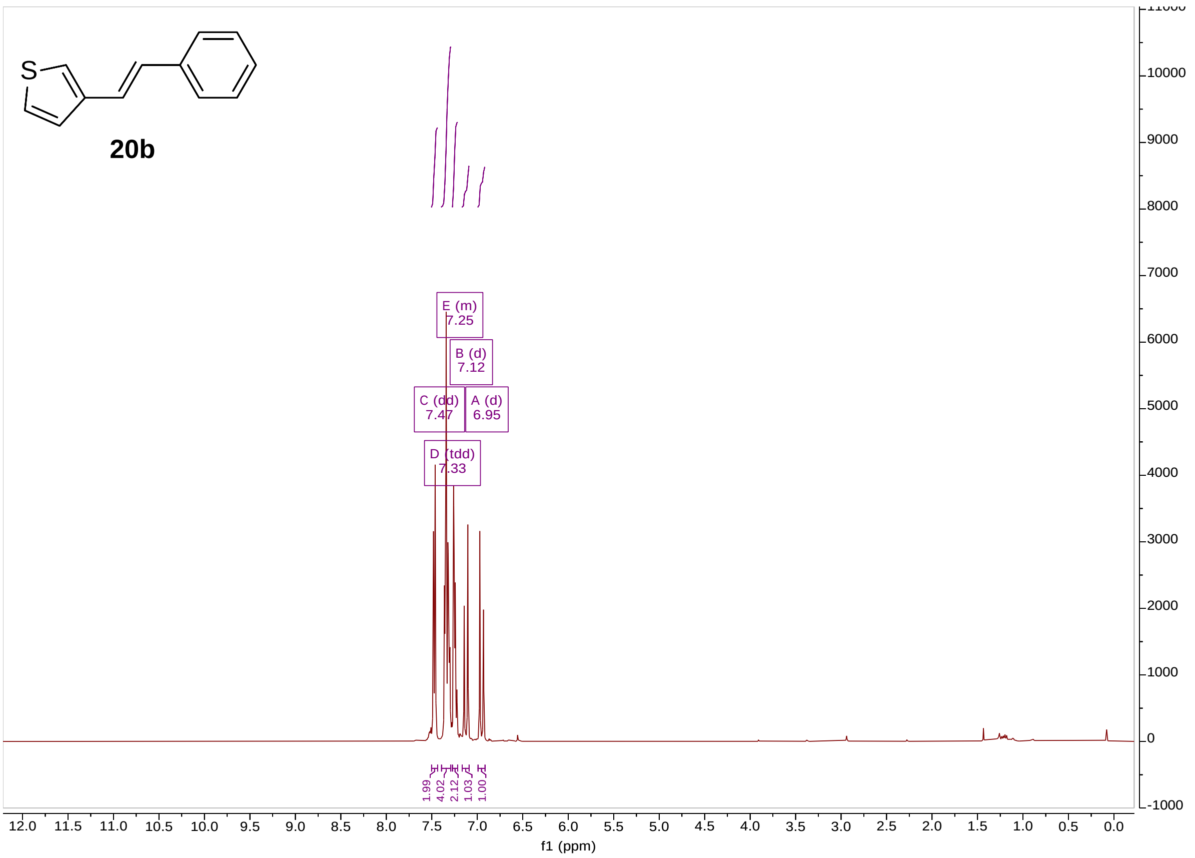

Figure S85. ^{1}H-NMR spectra of compound (**20b**).

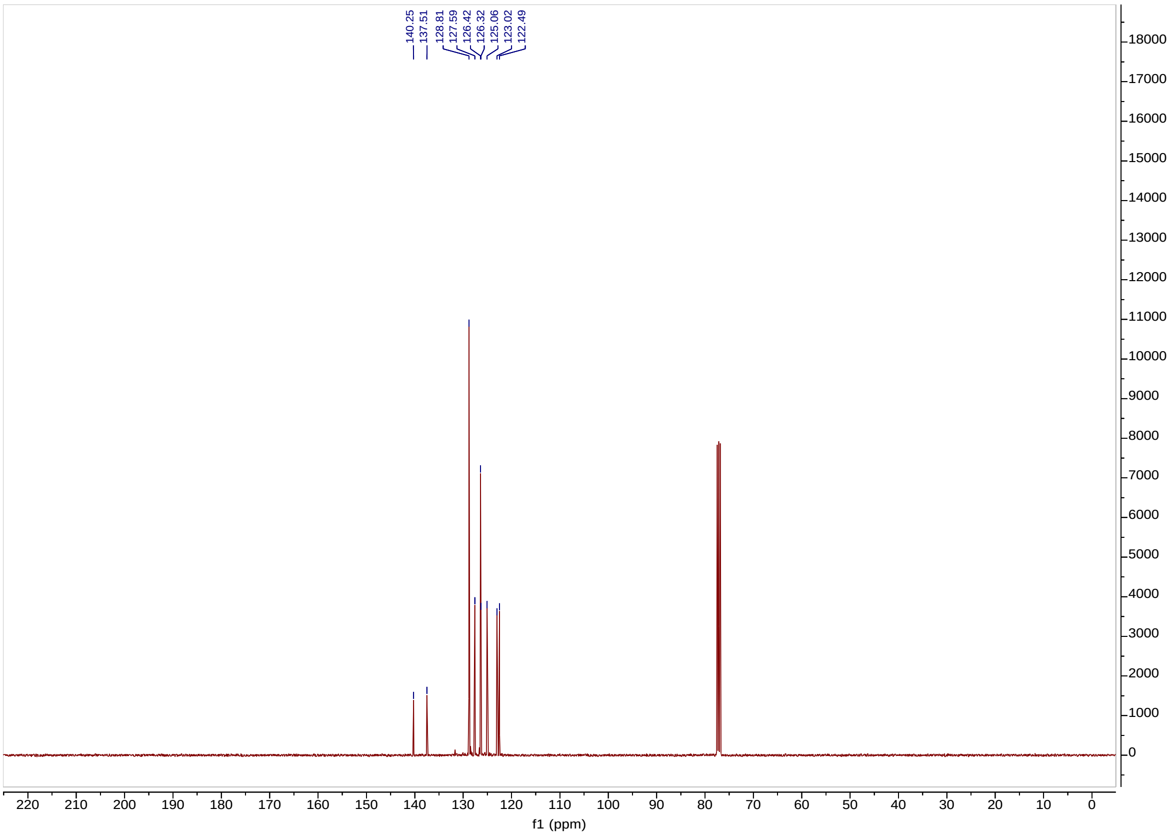

Figure S86. ^{13}C-NMR spectra of compound (**20b**).

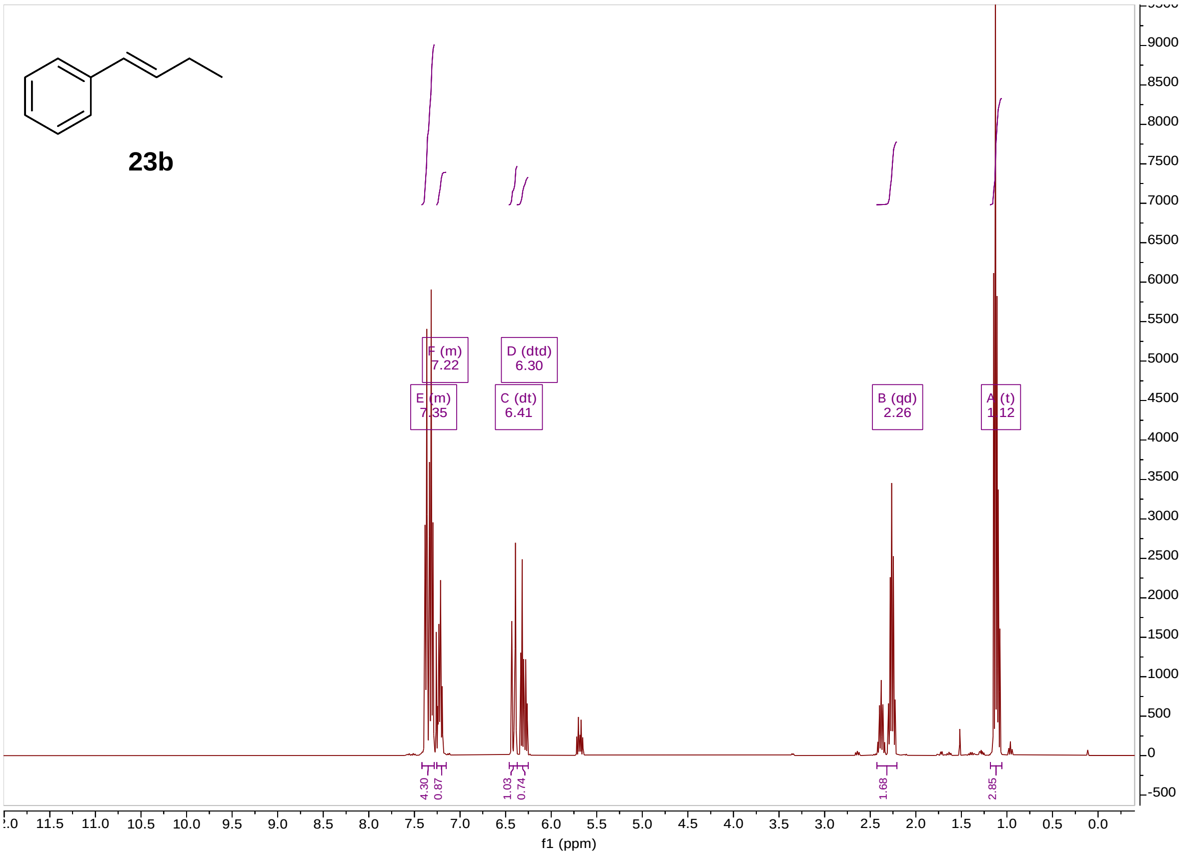

Figure S87. ^{1}H-NMR spectra of compound (**23b**).

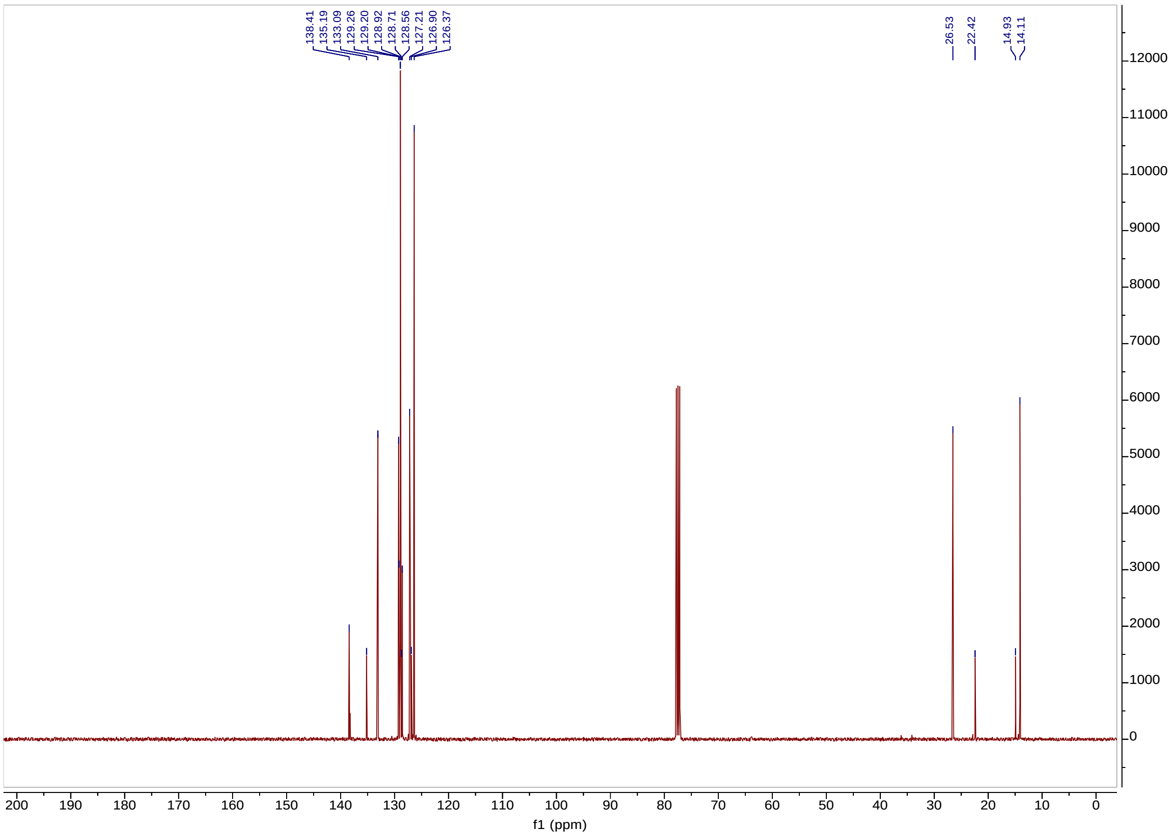

Figure S88. ^{13}C-NMR spectra of compound (**23b**).

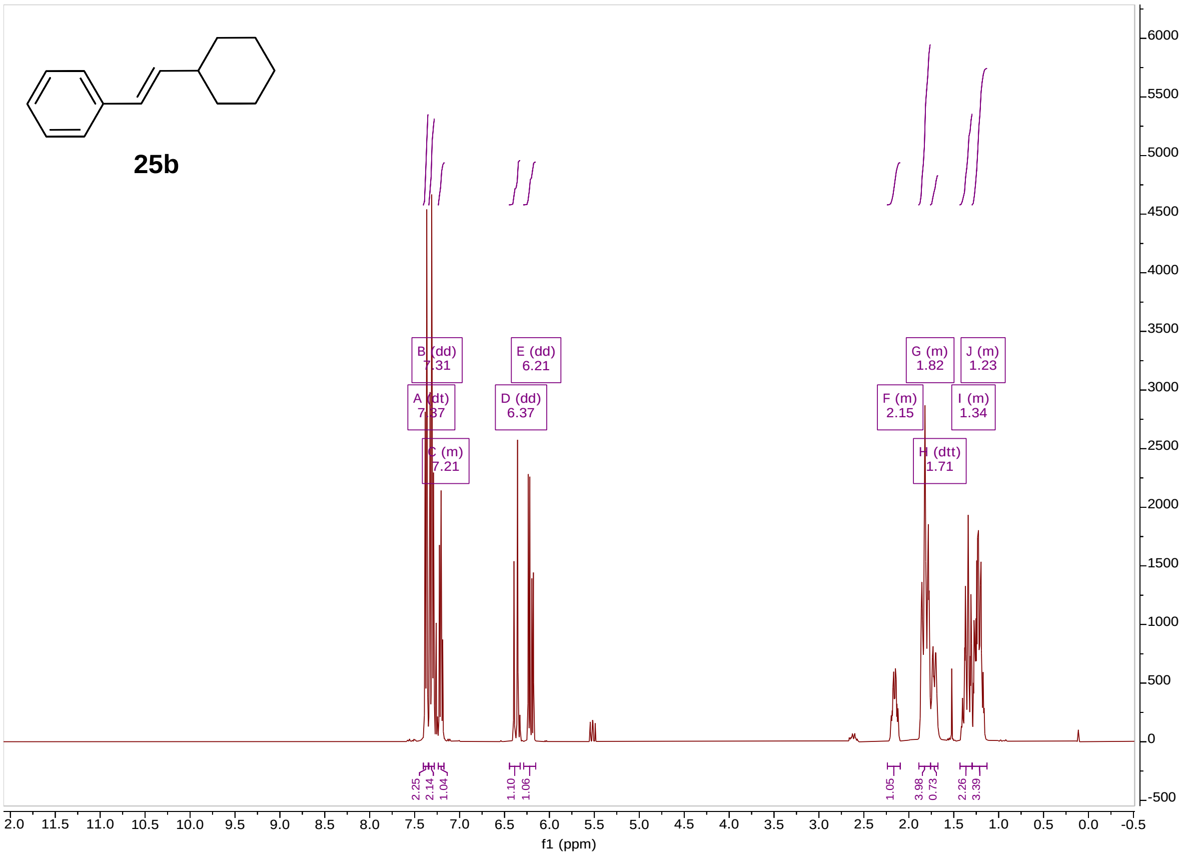

Figure S89. ^{1}H-NMR spectra of compound (**25b**).

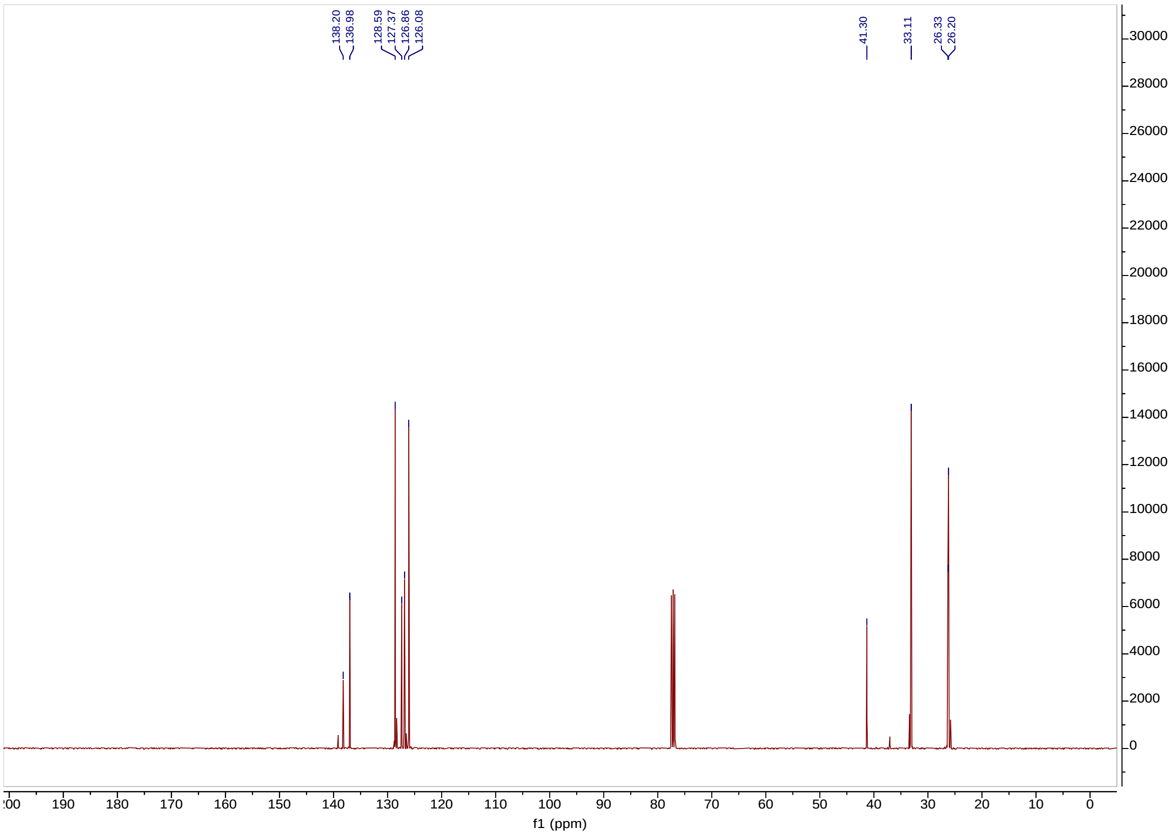

Figure S90. ^{13}C-NMR spectra of compound (**25b**).

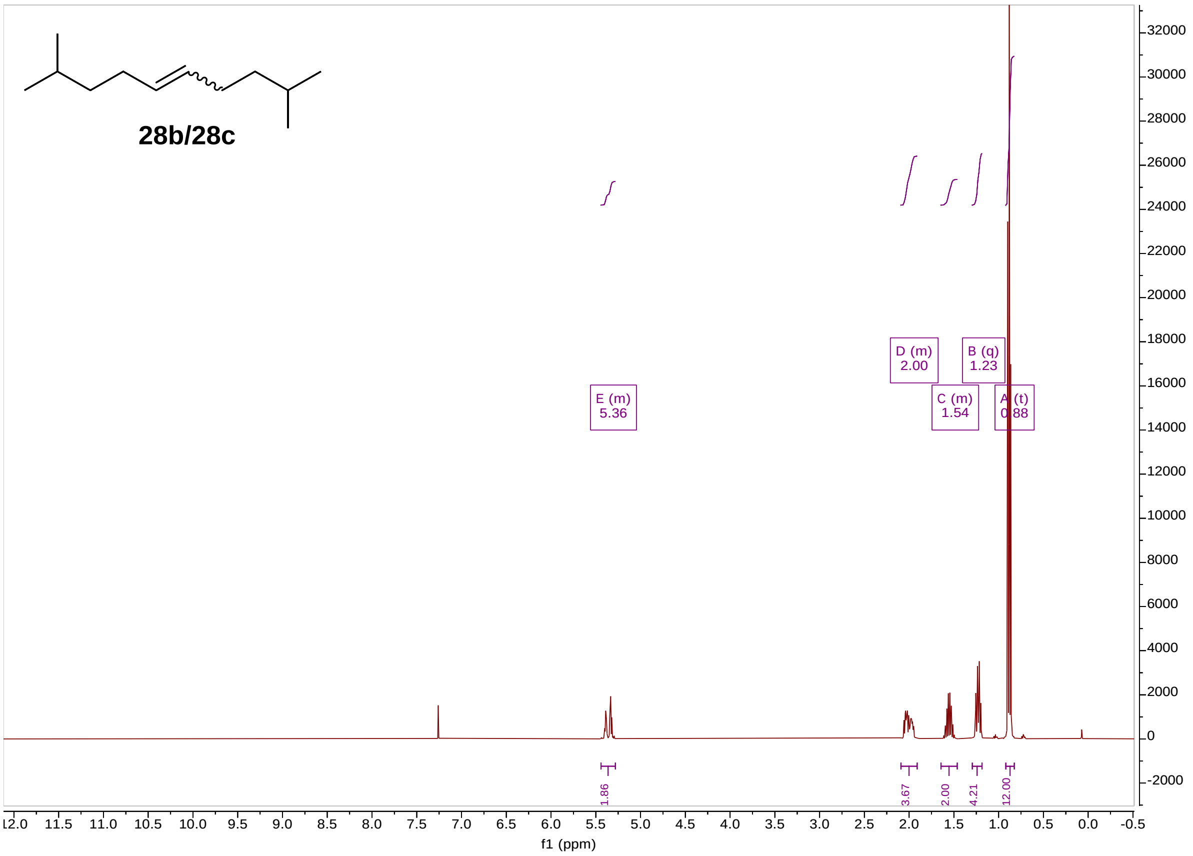

Figure S91. ^{1}H-NMR spectra of compound (**28b/c**).

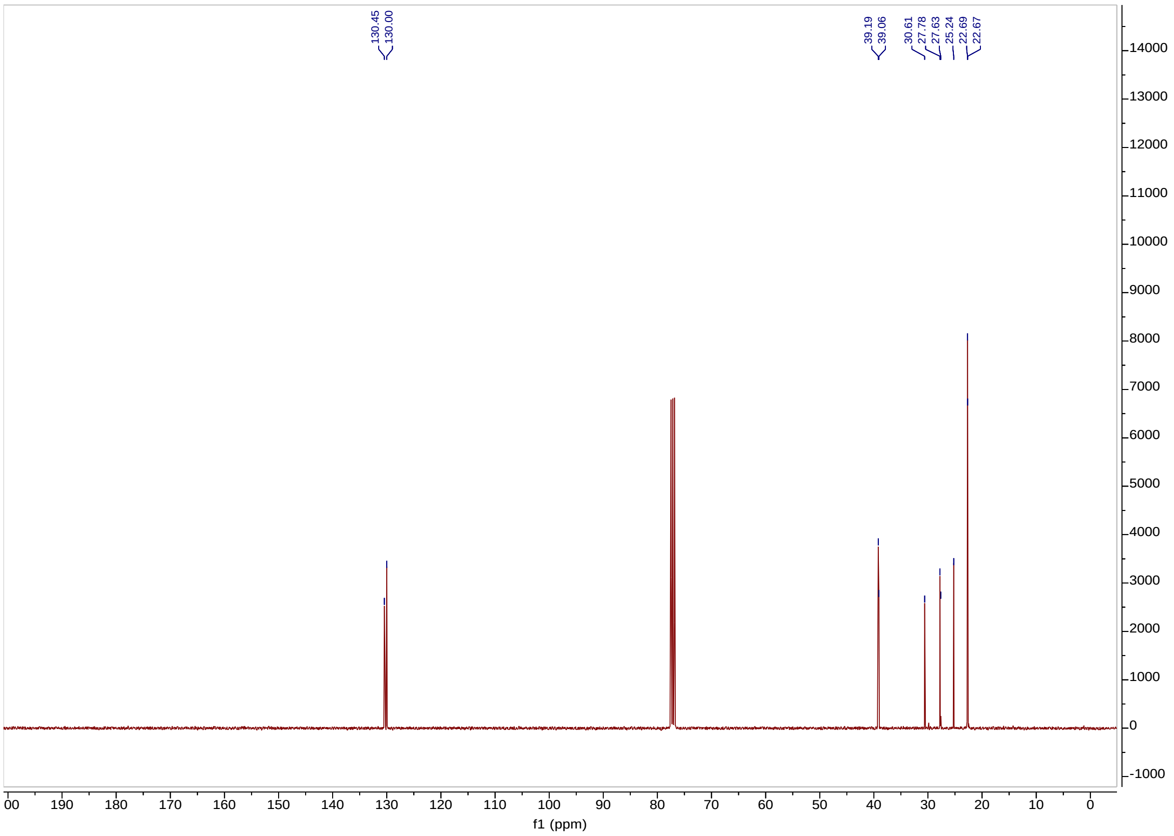

Figure S92. ^{13}C-NMR spectra of compound (**28b/c**).

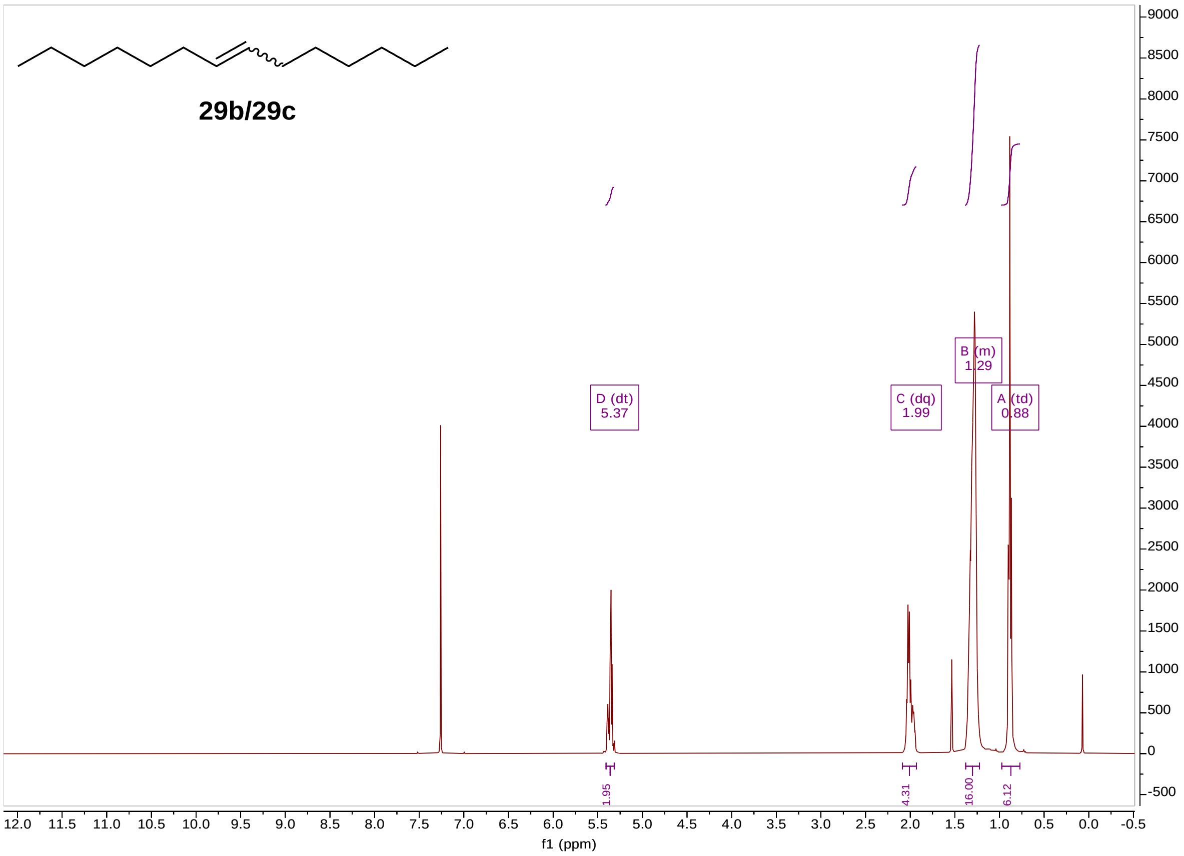

Figure S93. ^{1}H-NMR spectra of compound (**29b/c**).

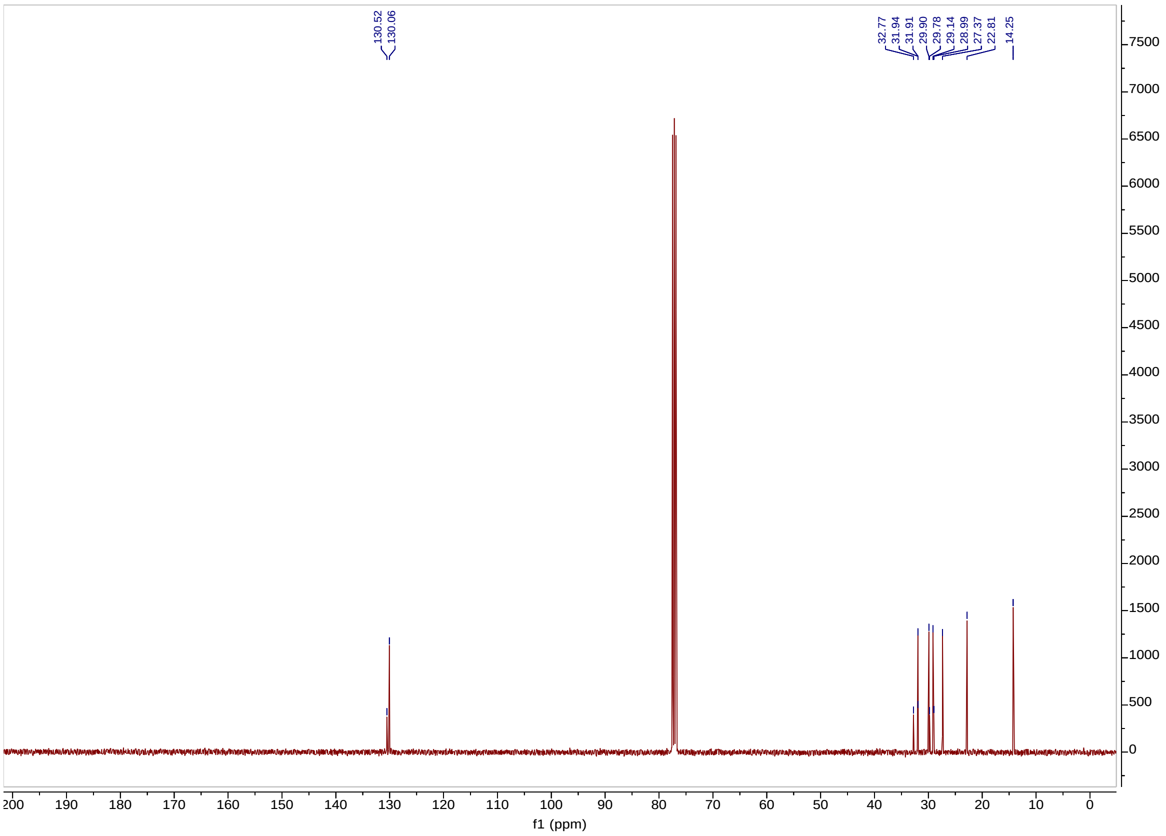

Figure S94. ^{13}C-NMR spectra of compound (**29b/c**).

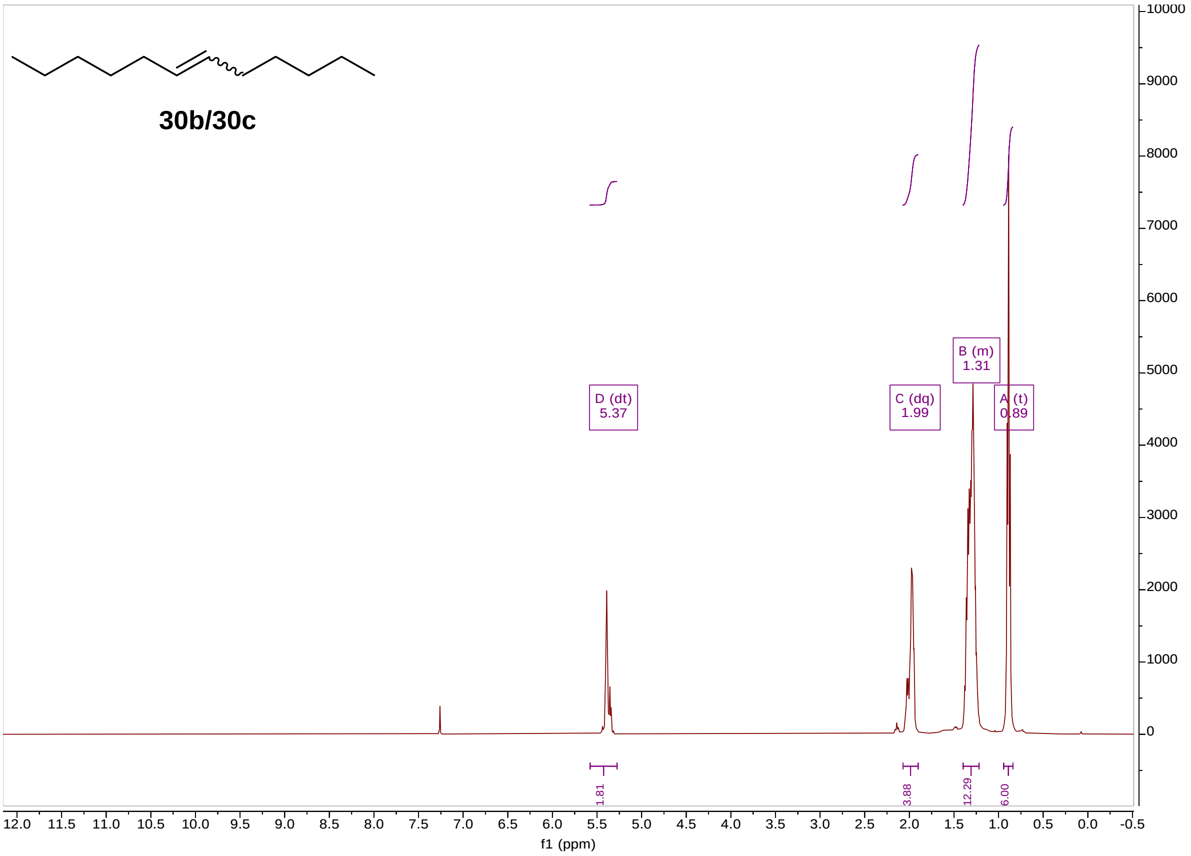

Figure S95. ^{1}H-NMR spectra of compound (**30b/c**).

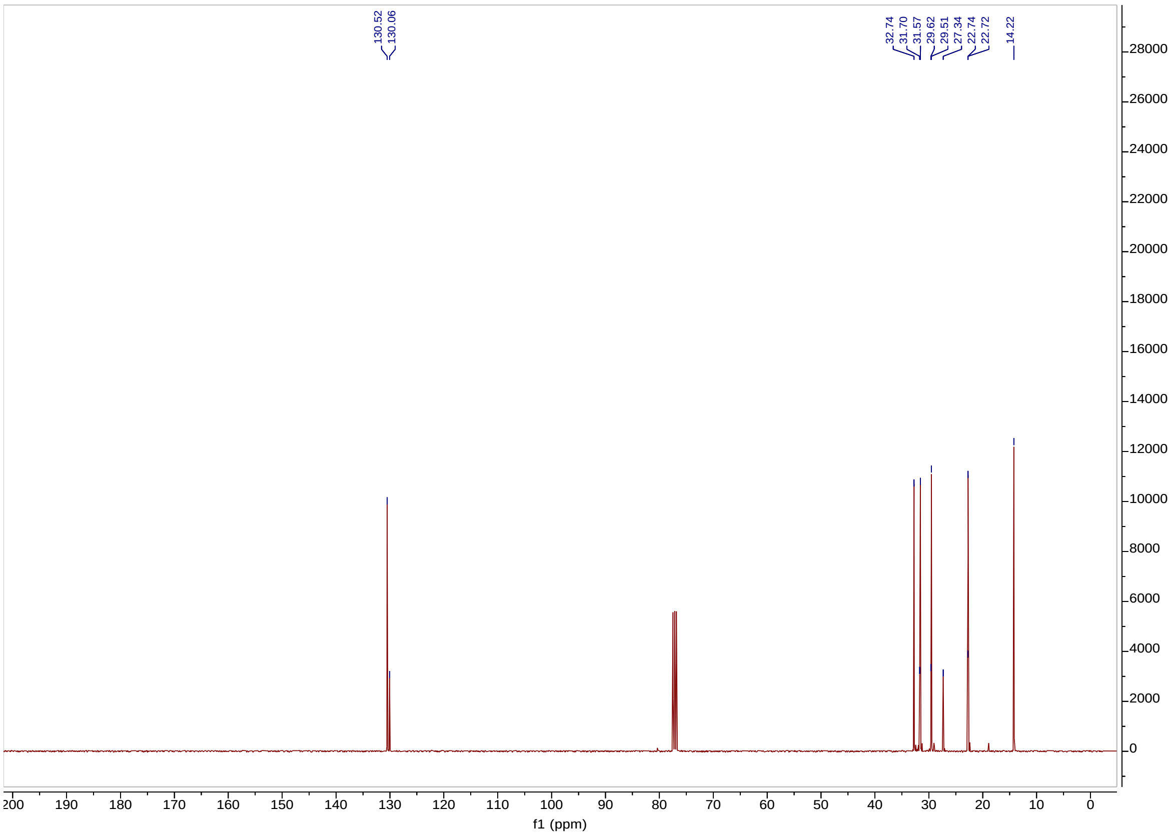

Figure S96. ^{13}C-NMR spectra of compound (**30b/c**).

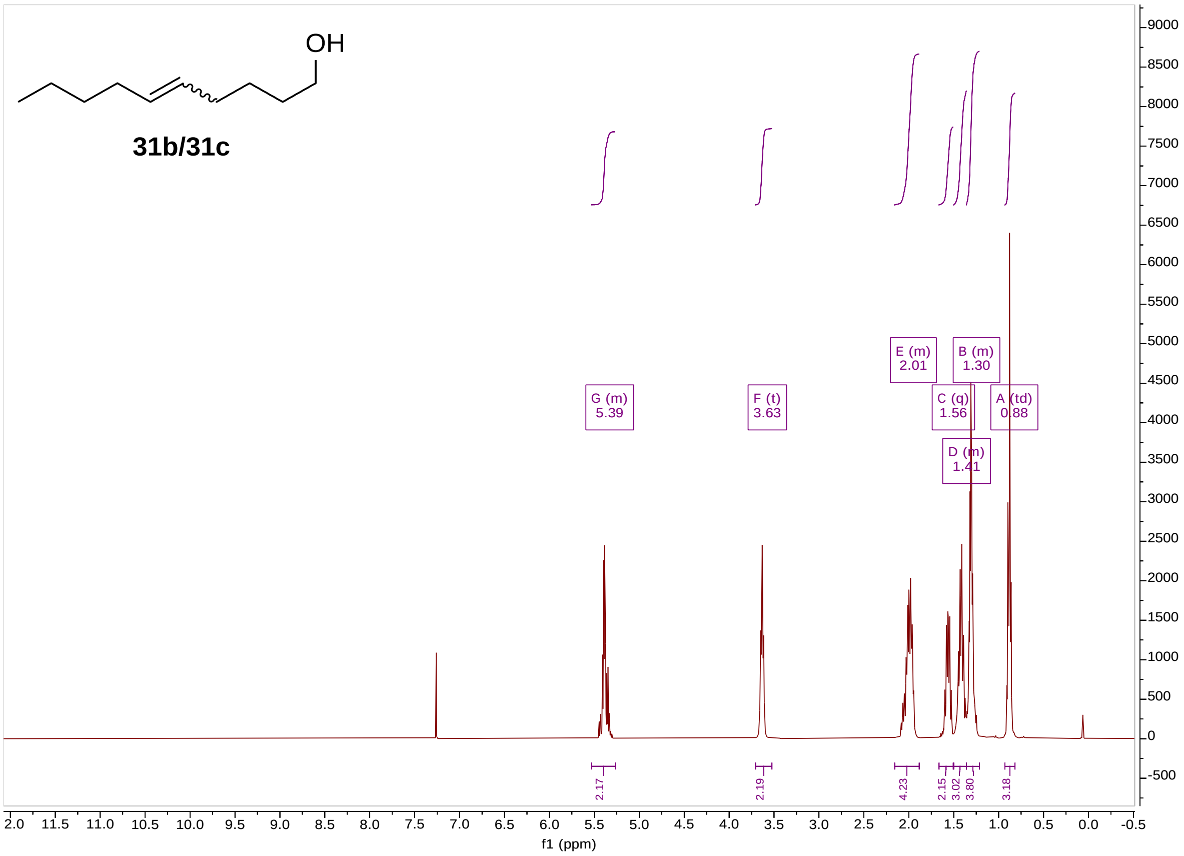

Figure S97. ^{1}H-NMR spectra of compound (**31b/c**).

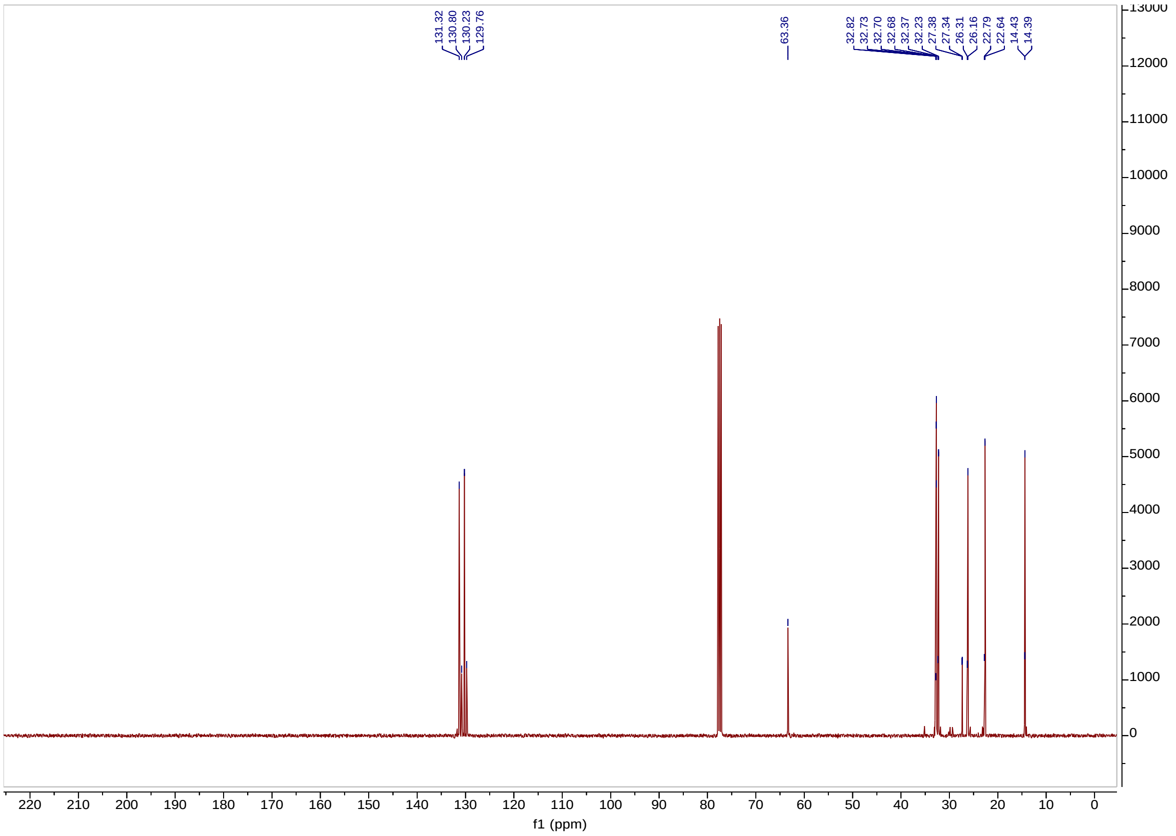

Figure S98. ^{13}C-NMR spectra of compound (**31b/c**).

S10. References

(1) (a) Sonogashira, K. Development of Pd-Cu catalyzed cross-coupling of terminal acetylenes with sp2-carbon halides. *J. Organomet. Chem.* **2002,** *653*, 46-49. (b) Schilz, M.; Plenio, H. A Guide to Sonogashira Cross-Coupling Reactions: The Influence of Substituents in Aryl Bromides, Acetylenes, and Phosphines. *J. Org. Chem.* **2012,** *77*, 2798-2807.

(2) Weber, S.; Stoeger, B.; Veiros, L. F.; Kirchner, K. Rethinking Basic Concepts-Hydrogenation of Alkenes Catalyzed by Bench-Stable Alkyl Mn(I) Complexes. *ACS Catal.* **2019,** *9*, 9715-9720.

(3) Gaussian 09, Revision **A.01**, Frisch, M. J.; Trucks, G. W.; Schlegel, H. B.; Scuseria, G. E.; Robb, M. A.; Cheeseman, J. R.; Scalmani, G.; Barone, V.; Mennucci, B.; Petersson, G. A.; Nakatsuji, H.; Caricato, M.; Li, X.; Hratchian, H. P.; Izmaylov, A. F.; Bloino, J.; Zheng, G.; Sonnenberg, J. L.; Hada, M.; Ehara, M.; Toyota, K.; Fukuda, R.; Hasegawa, J.; Ishida, M.; Nakajima, T.; Honda, Y.; Kitao, O.; Nakai, H.; Vreven, T.; Montgomery, Jr., J. A.; Peralta, J. E.; Ogliaro, F.; Bearpark, M.; Heyd, J. J.; Brothers, E.; Kudin, K. N.; Staroverov, V. N.; Kobayashi, R.; Normand, J.; Raghavachari, K.; Rendell, A.; Burant, J. C.; Iyengar, S. S.; Tomasi, J.; Cossi, M.; Rega, N.; Millam, J. M.; Klene, M.; Knox, J. E.; Cross, J. B.; Bakken, V.; Adamo, C.; Jaramillo, J.; Gomperts, R.; Stratmann, R. E.; Yazyev, O.; Austin, A. J.; Cammi, R.; Pomelli, C.; Ochterski, J. W.; Martin, R. L.; Morokuma, K.; Zakrzewski, V. G.; Voth, G. A.; Salvador, P.; Dannenberg, J. J.; Dapprich, S.; Daniels, A. D.; Farkas, Ö.; Foresman, J. B.; Ortiz, J. V.; Cioslowski, J.; Fox, D. J. Gaussian, Inc., Wallingford CT, **2009**.

(4) (a) Haeusermann, U.; Dolg, M.; Stoll, H.; Preuss, H.; Schwerdtfeger, P.; Pitzer, R. M. Accuracy of energy-adjusted quasirelativistic ab initio pseudopotentials *Mol. Phys.* **1993**, *78*, 1211-1224. (b) Kuechle, W.; Dolg, M.; Stoll, H.; Preuss, H. Energy-adjusted pseudopotentials for the actinides. Parameter sets and test calculations for thorium and thorium monoxide *J. Chem. Phys.* **1994**, *100*, 7535-7542. (c) Leininger, T.; Nicklass, A.; Stoll, H.; Dolg, M.; Schwerdtfeger, P. The accuracy of the pseudopotential approximation. II. A comparison of various core sizes for indium pseudopotentials in calculations for spectroscopic constants of InH, InF, and InCl *J. Chem. Phys.* **1996**, *105*, 1052-1059.

(5) (a) Ditchfield, R.; Hehre, W. J.; Pople, J. A. Self-Consistent Molecular-Orbital Methods. IX. An Extended Gaussian-Type Basis for Molecular-Orbital Studies of Organic Molecules *J. Chem. Phys.* **1971**, *54*, 724-728. (b) Hehre, W. J.; Ditchfield, R.; Pople, J. A. Self-Consistent Molecular Orbital Methods. 12. Further extensions of Gaussian-type basis sets for use in molecular-orbital studies of organic-molecules *J. Chem. Phys.* **1972**, *56*, 2257-2261. (c) Hariharan, P. C.; Pople, J. A. Accuracy of AH equilibrium geometries by single determinant molecular-orbital theory *Mol. Phys.* **1974**, *27*, 209-214. d) Gordon, M. S. The isomers of silacyclopropane *Chem. Phys. Lett.* **1980**, *76*, 163-168. e) Hariharan, P. C.; Pople, J. A. Influence of polarization functions on molecular-orbital hydrogenation energies *Theor. Chim. Acta* **1973**, *28*, 213-222.

(6) Hehre, W. J., Radom, L., Schleyer, P. v.R. & Pople, J. A. *Ab Initio Molecular Orbital Theory*, John Wiley & Sons, NY, 1986.

(7) Parr, R. G.; Yang, W. Density Functional Theory of Atoms and Molecules; Oxford University Press: New York, 1989.

(8) (a) Perdew, J. P.; Burke, K.; Ernzerhof, M. Generalized Gradient Approximation Made Simple *Phys. Rev. Lett.* **1996**, *77*, 3865-3868. (b) Perdew, J. P.; Burke, K.; Ernzerhof, M. Generalized Gradient Approximation Made Simple *Phys. Rev. Lett.* **1997**, *78*, 1396-1396. (c) Perdew, J. P. Density-functional approximation for the correlation energy of the inhomogeneous electron gas *Phys. Rev. B* **1986**, *33*, 8822-8824.

(9) (a) Peng, C.; Ayala, P. Y.; Schlegel, H. B.; Frisch, M. J. Using redundant internal coordinates to optimize equilibrium geometries and transition states *J. Comp. Chem.* **1996**, *17*, 49-56. (b) Peng, C.; Schlegel, H. B. Combining Synchronous Transit and Quasi-Newton Methods for Finding Transition States *Israel J. Chem.* **1993**, *33*, 449-454.

(10) (a) Cancès, M. T.; Mennucci, B.; Tomasi, J. A new integral equation formalism for the polarizable continuum model: Theoretical background and applications to isotropic and anistropic dielectrics *J. Chem. Phys.* **1997**, *107*, 3032-3041. (b) Cossi, M.; Barone, V.; Mennucci, B.; Tomasi, J. Ab initio study of ionic solutions by a polarizable continuum dielectric model *Chem. Phys. Lett.* **1998**, *286*, 253-260. (c) Mennucci, B.; Tomasi, J. Continuum solvation models: A new approach to the problem of solute's charge distribution and cavity boundaries *J. Chem. Phys.* **1997**, *106*, 5151-5158. (d) Tomasi, J.; Mennucci, B.; Cammi, R. Quantum mechanical continuum solvation models *Chem. Rev.* **2005**, *105*, 2999-3094.

(11) Marenich, A. V.; Cramer, C. J.; Truhlar, D. G. Universal solvation model based on solute electron density and a continuum model of the solvent defined by the bulk dielectric constant and atomic surface tensions *J. Phys. Chem. B*, **2009**, *113*, 6378-6396.

(12) Fu, S.; Chen, N.-Y.; Liu, X.; Shao, Z.; Luo, S.-P.; Liu, Q. Ligand-Controlled Cobalt-Catalyzed Transfer Hydrogenation of Alkynes: Stereodivergent Synthesis of Z- and E-Alkenes. *J. Am. Chem. Soc.* **2016,** *138*, 8588-8594.

(13) Civicos, J. F.; Alonso, D. A.; Najera, C. Microwave-promoted copper-free Sonogashira-Hagihara couplings of aryl imidazolylsulfonates in water. *Adv. Synth. Catal.* **2013,** *355*, 203-208.

(14) Saetan, T.; Lertvachirapaiboon, C.; Ekgasit, S.; Sukwattanasinitt, M.; Wacharasindhu, S. Palladium Nanoparticles Immobilized on Individual Calcium Carbonate Plates Derived from Mussel Shell Waste: An Ecofriendly Catalyst for the Copper-Free Sonogashira Coupling Reaction. *Chem. - Asian J.* **2017,** *12*, 2221-2230.

(15) Panda, B.; Sarkar, T. K. Gold and palladium combined for the Sonogashira-type cross-coupling of arenediazonium salts. *Chem. Commun.* **2010,** *46*, 3131-3133.

(16) Sagadevan, A.; Hwang, K. C. Photo-Induced Sonogashira C-C Coupling Reaction Catalyzed by Simple Copper(I) Chloride Salt at Room Temperature. *Adv. Synth. Catal.* **2012,** *354*, 3421-3427.

(17) Bhaskar, R.; Sharma, A. K.; Yadav, M. K.; Singh, A. K. Sonogashira (Cu and amine free) and Suzuki coupling in air catalyzed via nanoparticles formed in situ from Pd(II) complexes of chalcogenated Schiff bases of 1-naphthaldehyde and their reduced forms. *Dalton Trans.* **2017,** *46*, 15235-15248.

(18) Marzo, L.; Perez, I.; Yuste, F.; Aleman, J.; Garcia Ruano, J. L. A straightforward alkynylation of Li and Mg metalated heterocycles with sulfonylacetylenes. *Chem. Commun.* **2015,** *51*, 346-349.

(19) Hu, H.; Yang, F.; Wu, Y. Palladacycle-Catalyzed Deacetonative Sonogashira Coupling of Aryl Propargyl Alcohols with Aryl Chlorides. *J. Org. Chem.* **2013,** *78*, 10506-10511.

(20) Kakusawa, N.; Yamaguchi, K.; Kurita, J. Palladium-catalyzed cross-coupling reaction of ethynylstibines with organic halides. J. Organomet. Chem. 2005, 690, 2956-2966.

(21) Gregori, B. J.; Nowakowski, M.; Schoch, A.; Poellath, S.; Zweck, J.; Bauer, M.; Jacobi von Wangelin, A. Stereoselective Chromium-Catalyzed Semi-Hydrogenation of Alkynes. *ChemCatChem* **2020,** *12*, 5359-5363.

(22) Rami, F.; Baechtle, F.; Plietker, B. Hydroboration of internal alkynes catalyzed by $FeH(CO)(NO)(PPh_3)_2$: a case of boron-source controlled regioselectivity. *Catal. Sci. Technol.* **2020,** *10*, 1492-1497.

(23) Murugesan, K.; Bheeter, C. B.; Linnebank, P. R.; Spannenberg, A.; Reek, J. N. H.; Jagadeesh, R. V.; Beller, M. Nickel-catalyzed stereodivergent synthesis of E- and Z-alkenes by hydrogenation of alkynes. *ChemSusChem* **2019,** *12*, 3363-3369.

(24) Gong, D.; Hu, B.; Yang, W.; Kong, D.; Xia, H.; Chen, D. A Bidentate Ru(II)-NC Complex as a Catalyst for Semihydrogenation of Alkynes to (E)-Alkenes with Ethanol. *Organometallics* **2020,** *39*, 862-869.

(25) Bourne, S. L.; O'Brien, M.; Kasinathan, S.; Koos, P.; Tolstoy, P.; Hu, D. X.; Bates, R. W.; Martin, B.; Schenkel, B.; Ley, S. V. Flow Chemistry Syntheses of Styrenes, Unsymmetrical Stilbenes and Branched Aldehydes. *ChemCatChem* **2013,** *5*, 159-172.

(26) Cao, C.-T.; Yuan, H.; Zhu, Q.; Cao, C. Determining the excited-state substituent constants σCCoex of ortho-substituents from 2,4'-disubstituted stilbenes. *J. Phys. Org. Chem.* **2019,** *32*, e3962

(27) Park, B. Y.; Lim, T.; Han, M. S. A simple and efficient in situ generated copper nanocatalyst for stereoselective semihydrogenation of alkynes. *Chem. Commun. (Cambridge, U. K.)* **2021,** *57*, 6891-689.

(28) McNulty, J.; Das, P. Highly Stereoselective and General Synthesis of (E)-Stilbenes and Alkenes by Means of an Aqueous Wittig Reaction. *Eur. J. Org. Chem.* **2009**, 4031-4035.

(29) Yang, H.; Dong, W.; Wang, W.; Li, T.; Zhao, W. Stereoselective Rhodium-Catalyzed Isomerization of Stereoisomeric Mixtures of Arylalkenes. *Synthesis* **2020,** *52*, 2833-2840.

(30) Yadav, S.; Dutta, I.; Saha, S.; Das, S.; Pati, S. K.; Choudhury, J.; Bera, J. K. An Annelated Mesoionic Carbene (MIC) Based Ru(II) Catalyst for Chemo- and Stereoselective Semihydrogenation of Internal and Terminal Alkynes. *Organometallics* **2020,** *39*, 3212-3223.

(31) Ekebergh, A.; Begon, R.; Kann, N. Ruthenium-Catalyzed E-Selective Alkyne Semihydrogenation with Alcohols as Hydrogen Donors. *J. Org. Chem.* **2020,** *85*, 2966-2975.

(32) Yu, X.; Zhao, H.; Li, P.; Koh, M. J. Iron-Catalyzed Tunable and Site-Selective Olefin Transposition. *J. Am. Chem. Soc.* **2020,** *142*, 18223-18230.

(33) Maazaoui, R.; Abderrahim, R.; Chemla, F.; Ferreira, F.; Perez-Luna, A.; Jackowski, O. Catalytic Chemoselective and Stereoselective Semihydrogenation of Alkynes to E-Alkenes Using the Combination of Pd Catalyst and ZnI_2. *Org. Lett.* **2018,** *20*, 7544-7549.

(34) Semba, K.; Fujihara, T.; Xu, T.; Terao, J.; Tsuji, Y. Copper-Catalyzed Highly Selective Semihydrogenation of Non-Polar Carbon-Carbon Multiple Bonds using a Silane and an Alcohol. *Adv. Synth. Catal.* **2012,** *354*, 1542-1550.

(35) Werner, E. W.; Sigman, M. S. Operationally Simple and Highly (E)-Styrenyl-Selective Heck Reactions of Electronically Nonbiased Olefins. *J. Am. Chem. Soc.* **2011,** *133*, 9692-9695.

(36) Ma, X.; Hazelden, I. R.; Langer, T.; Munday, R. H.; Bower, J. F. Enantioselective Aza-Heck Cyclizations of N-(Tosyloxy)carbamates: Synthesis of Pyrrolidines and Piperidines. *J. Am. Chem. Soc.* **2019,** *141*, 3356-3360.

(37) Hazelden, I. R.; Carmona, R. C.; Langer, T.; Pringle, P. G.; Bower, J. F. Pyrrolidines and Piperidines by Ligand-Enabled Aza-Heck Cyclizations and Cascades of N-(Pentafluorobenzoyloxy)carbamates. *Angew. Chem., Int. Ed.* **2018,** *57*, 5124-5128.
